# The Effect of Nanoparticle Structure on the Thermodynamics and Kinetics of Proton-Coupled Electron Transfer Reactions to V_2_O_5_

**DOI:** 10.1021/jacs.6c08557

**Published:** 2026-07-14

**Authors:** Osman Bunjaku, Mustafa Turan, Hannes V. Beyertt, Wael Barakat, Michael Dyballa, Bertold Rasche, Deven P. Estes

**Affiliations:** Faculty of Chemistry, 9149 University of Stuttgart, Pfaffenwaldring 55, Stuttgart 70569, Germany

## Abstract

Proton-coupled electron transfers (PCETs) to metal oxides are key reactions for sustainable catalytic and energy storage processes. The factors that control PCET reactions on metal oxides are, however, not well understood, with the effect of particle morphology/surface faceting on the thermodynamics and kinetics of PCET being essentially untested. We measured the thermodynamics and kinetics of PCET from CpCr­(CO)_3_H to five V_2_O_5_ samples of varying morphologies/surface faceting. Their nanostructures were assessed by Rietveld refinement accounting for preferred crystallite orientation, giving a semiquantitative measurement of surface faceting. PCET to V_2_O_5_ occurs via proton insertion-coupled electron transfer (PICET), where H· diffuses into the bulk of the particle. The thermodynamics of PICET are controlled by particle structure, with nonequilibrium morphologies requiring structural rearrangement during PICET. The kinetics of PICET are controlled by the rate of H· diffusion into the bulk, with diffusion along the interconnected V_2_O_5_ layers being 10x faster than between the layers. Samples with hindered H· diffusion also show low activity in the oxidation of methanol, demonstrating that diffusion of H· into the bulk occurs during catalysis. This work demonstrates that particle morphology is critical for PCET reactions to anisotropic metal oxides and must be considered when examining their reactivity.

## Graphical abstract

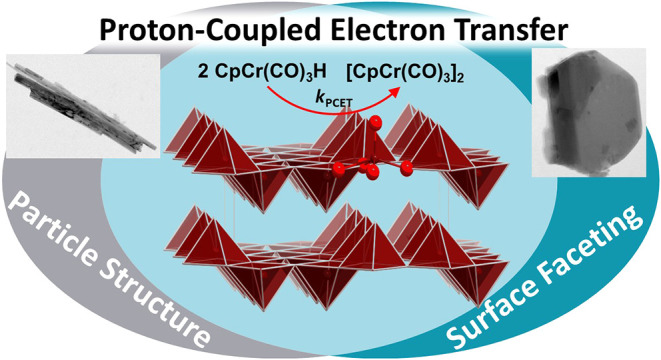

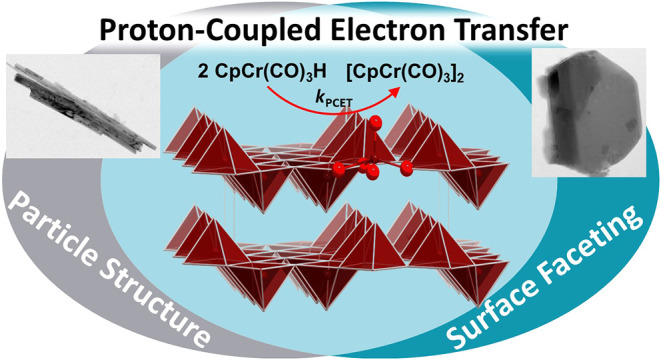

## Introduction

Proton-coupled electron transfer (PCET) to and from metal oxides (producing new OH groups and reduced metal centers) is a critical step in a number of key technologies for a sustainable society. For example, PCET to metal oxides are responsible for creating the catalytically active sites for alkane hydrocracking^1–3^ and Cu/ZnO/Al_2_O_3_ catalyzed methanol synthesis from CO_2_ (where the ZnO is reduced by PCET from H on the Cu surface).^4,5^ PCET is also often a key or even rate-determining step in various catalytic reactions such as selective hydrocarbon oxidations,^6–9^ numerous electrocatalytic reactions,^10–24^ and production of olefins from alkanes by oxidative dehydrogenation.^25–28^ PCET is also the direct mechanism of energy storage in the promising field of aqueous metal oxide batteries.^29^ Therefore, optimizing the amount of PCET that occurs to interesting metal oxides is a critical goal for modern materials chemists. Despite the immense importance of many of these applications, we still know relatively little about the factors that control PCET to individual metal oxide samples. The effect of variations in the structure of metal oxide nanoparticles (in particular, variation of particle morphology and surface faceting) on the thermodynamics and kinetics of PCET to metal oxides has been particularly challenging to understand.

Over the last two decades chemists have begun studying the underlying thermodynamics and kinetics of PCET to various metal oxides. Matson and co-workers have studied the thermodynamics and kinetics of PCET to polyoxometalate clusters of many different elements (as models of metal oxides) and found that the BDFE­(OH) goes down at higher H· loading/degree of reduction.^30–33^ Mayer and Gamelin found that colloidal CeO_2_, TiO_2_, NiO, and ZnO nanoparticles are proton–electron donors and acceptors.^34–37^ Recently they were able to correlate the sizes of CeO_2_ particles to their PCET reactivity, with smaller particles being better H· acceptors than larger particles at the same degree of reduction.^36^ Augustyn, Ballard, Matson and others have demonstrated that PCET reactions in some metal oxides result in transport of the H· into the bulk phase in a reaction called proton-insertion coupled electron transfer (PICET).^29,38^ PICET reactions are distinct from PCET reactions on surfaces in that they have the additional step of diffusion of H· throughout the material that can in some cases be rate-limiting.^39^ Augustyn and co-workers recently demonstrated that factors such as heteroatom doping in WO_3_ can affect PICET reactions.^40^ However, the relationship between metal oxide morphology and their PCET reactivity is still not understood.

A promising strategy for studying the effects of subtle variations in nanoparticle structure on the thermodynamics and kinetics of PCET to metal oxides is via model reaction with transition-metal hydrides. The Estes group showed that transition metal hydrides with weak M-H bonds (such as CpCr­(CO)_3_H, Cr–H) can be used as H· donors, allowing us to measure the thermodynamics and kinetics of PCET to metal oxides.^41^ Similar to the studies of Mayer et al. on CeO_2_,^36^ we showed that Cr–H reacts with CeO_2_ by PCET to reduce 60% of the surface Ce atoms, at which H· coverage the bond dissociation free energy (BDFE) of the Ce–OH groups was measured to be 57 kcal mol^–1^.^42^ Here, we carry out a systematic study on how variation of materials properties affects the PCET reactivity of vanadium pentoxide (V_2_O_5_) samples with varying surface areas, morphologies, and faceting (Figure 1). We used Rietveld refinement with modeling of preferred crystallite orientations to characterize the samples, which tells us not only the average particle size and structure, but also gives a semiquantitative measurement of the surface faceting/morphology of the nanoparticles. We measured the thermodynamics and kinetics of PCET from CrH to V_2_O_5_ using adsorption titrations and ReactIR measurements, respectively, both of which show PICET reactivity for V_2_O_5_. The extent of PCET on the V_2_O_5_ samples is dependent on morphology, with samples having a nonequilibrium surface faceting being less heavily reduced than thermodynamically equilibrated particles. One sample (V_2_O_5_-sheets) also undergoes PICET 10 times more slowly than other samples, due to the much lower proportion of [100] or [010] facets (along whose directions H· diffuses more quickly into the bulk material). These samples were then finally tested for their reactivity in the oxidation of methanol to formaldehyde. The sample exhibiting slow PICET also shows the lowest catalytic activity, suggesting that PICET or proton diffusion may be a relevant step in the catalytic cycle. This study demonstrates that the structure of nanoparticles (in addition to the particle sizes) must also be characterized in order to fully understand PCET reactivity in metal oxides.

**Figure 1:**
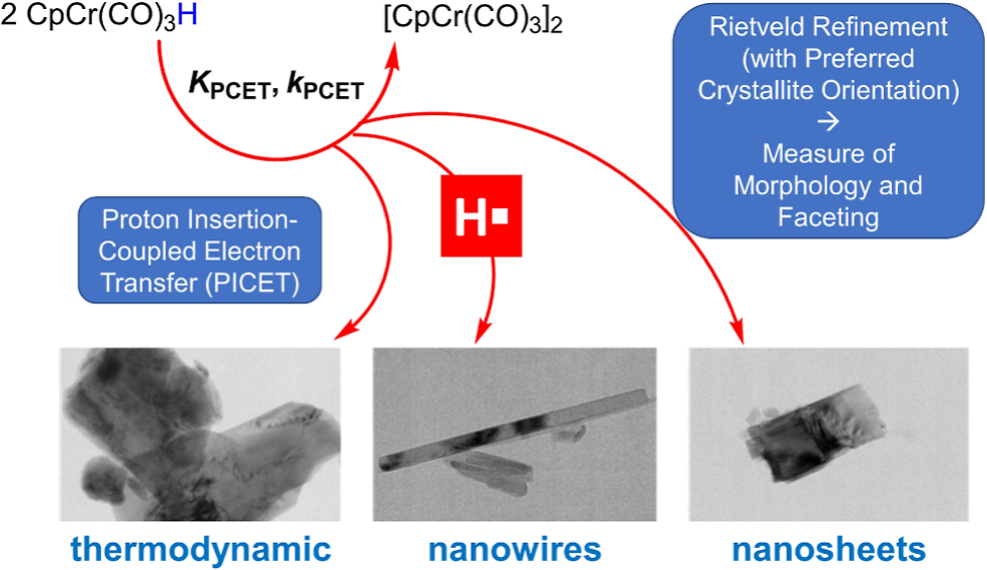
PCET from Cr–H to V_2_O_5_ samples with varying particle sizes, morphologies, and faceting.

## Results and Discussion

### Synthesis and Characterization of V_2_O_5_ Samples

We began by synthesizing V_2_O_5_ samples with various morphologies. The BET surface areas and average crystallite sizes, as extracted by Rietveld refinement of the powder X-ray diffractograms (PXRD, Figures S1 and S2 and Table S1) are given in Table 1 (vide infra). The crystallite sizes can be taken as representative of the particle sizes, but are usually not exactly the same, as a particle might consist of several crystallites. In addition, the particle sizes in these bulk samples tend to have a broad distribution, and as such should be taken as the average of a broad distribution. The HRTEM and SEM images of the synthesized samples are shown in Figures 2 and S4–S7. We used a commercial V_2_O_5_ sample with high crystallinity as a reference material (V_2_O_5_-comm), which showed a small surface area of 4.6(5) m^2^ g^–1^. Rietveld refinement of the PXRD showed that these nanoparticles have an average crystallite size of 85(1) nm and no preferred orientation (Figure S3a), suggesting that their surface faceting is most likely thermodynamically equilibrated to that of the Wulff construction for orthorhombic V_2_O_5_.^43^ We also increased the surface area (A_BET_) of V_2_O_5_-comm by ball milling.^44^ The powder was dry milled in a ball to powder mass ratio of 10:1 for 5 min giving a powder (V_5_O_5_-BM) with a surface area of 10.0 m^2^g^–1^ and a particle size of 98(1) nm. The slightly increased crystallite size is most likely the result of the amorphization of smaller particles in the ball mill. Again, we do not find any evidence for a preferred orientation suggesting thermodynamically equilibrated particles (Figure S3b).

**Table 1:** Structural, Thermodynamic, and Kinetic Measurements of PCET from Cr–H to V_2_O_5_

sample	A_BET_ (m^2^ g^–1^)	*d*_avg_ (nm)	*k*_PCET_ ( $g_{V_{2}O_{5}}^{-1}$ s^–1^)	PO (−)	*q*_H_ (mmol H g^–1^)	BDFE(OH)_min_ (kcal mol^–1^)	ΔBDFE(OH) (kcal mol^–1^)	*g* (a.u.)
V^_2_^O_5_-comm	4.6	85(1)	0.052(5)	none	11(2)	55.7(3)	0	–3.3(2)
V_2_O_5_-BM	10.0	98(1)	0.04(1)	none	10(2)	56.0(2)	0.3	0.84(7)
V_2_O_5_-coll	6.6	117(2)	0.05(1)	none	8.7(2)	57.9(1)	2.2	–1.3(2)
V_2_O_5_-sheets	15.0	77(1)	0.005(3)	(001)	6.6(9)	>61.9(3)	6.2	–6.0(8)
V_2_O_5_-wires	42.2	27(1)	0.05(1)	(h0l)	6.4(1.1)	>63.3(2)	7.6	–9.6(1)

**Figure 2:**
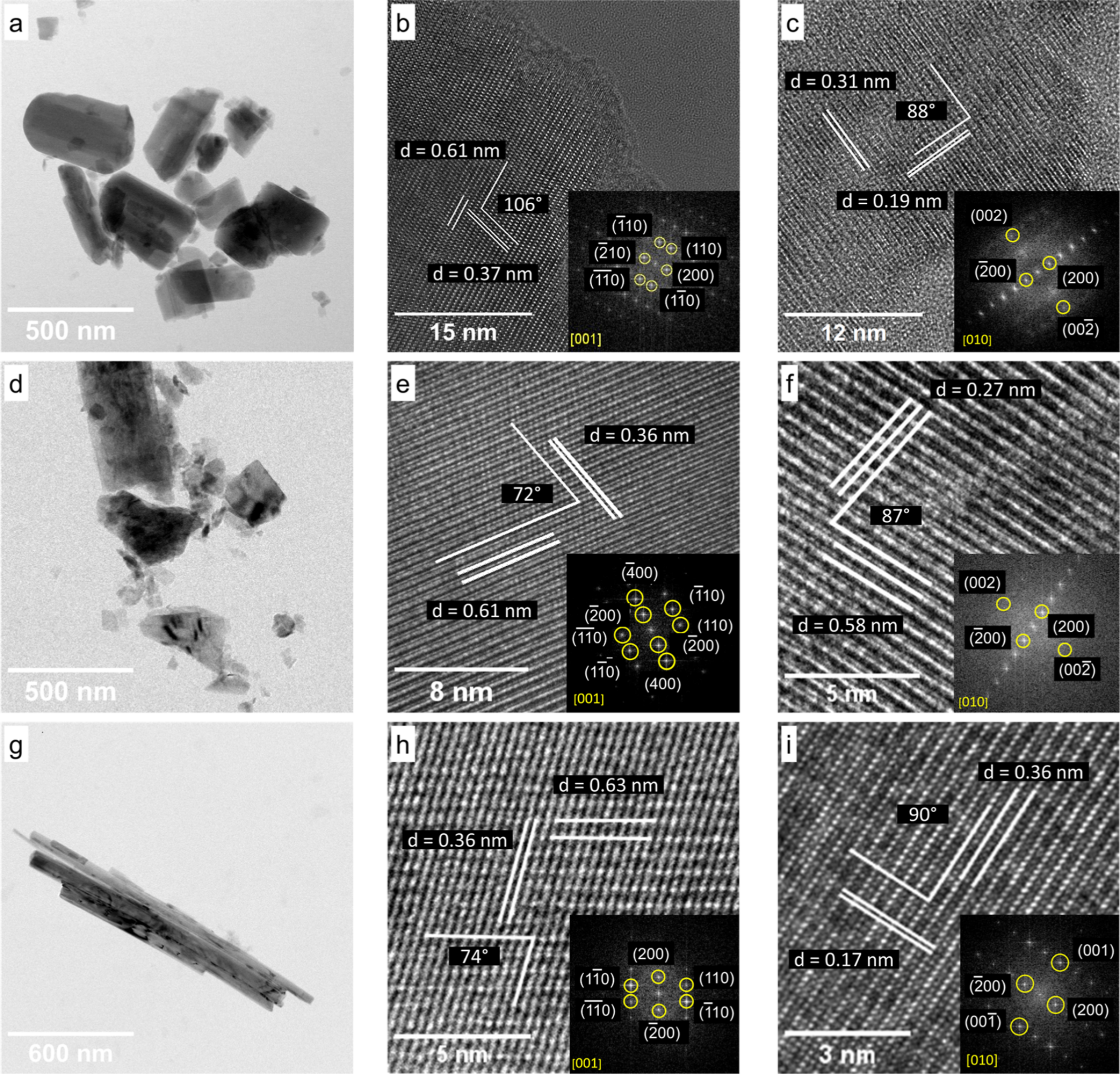
HRTEM images and fast Fourier transform patterns of (a) V_5_O_5_-coll with (b) [001] and (c) [010] crystal facets, (d) V_2_O_5_-sheets with (e) [001] and (f) [010] crystal facets and (g) V_2_O_5_-wires with (h) [001] and (i) [010] crystal facets.

We also synthesized colloidal V_2_O_5_ particles (V_2_O_5_-coll) by the procedure of Dhachapally^45^ via the precipitation of NH_4_VO_3_ with oxalic acid followed by drying and high temperature (500 °C) calcination. This resulted in particles of V_2_O_5_ with an average crystallite size of 117(2) nm and a BET surface area of 6(1) m^2^ g^–1^. This was confirmed by both SEM (Figure S4) and HRTEM (Figures S5 and 2a–c), which showed the presence of both [001] and [010] crystal facets. We also synthesized nanosheets of V_2_O_5_ (V_2_O_5_-sheets) using the method of Ghosh et al.^46^ V_2_O_5_ was reacted with H_2_O_2_ at 60 °C for 2.5 h, dried, then calcined at 400 °C for 2h. This yielded particles with the expected sheet like morphology (Figures 2d–f and S6), an average crystallite size of 77(1) nm, and a BET surface area of 15(2) m^2^ g^–1^. The sheet like morphology is also reflected in the preferred orientation along (001) observed in the XRD, meaning that the main face of the V_2_O_5_-sheets is perpendicular to the *c*-axis and that the [001] surface makes up much more of the surface than in other samples (Figures 2 and S3d). We finally synthesized V_2_O_5_ nanowires (V_2_O_5_-wires) according to a hydrothermal procedure also from Ghosh et al.^46^ This gave particles with the reported wire-like morphology (Figures 2g–i and S7), having a higher surface area of 42 m^2^g^–1^ and an average crystallite size of 27(1) nm, significantly smaller than the others. Again, the wire-like morphology is reflected in the preferred orientation observed in the XRD, where all orientations (h0l) show a preference, with the (100) and the (101) being particularly strong (Figures 2 and S3e). This implies a growth direction of the V_2_O_5_-wires along the *b*-axis and a slight faceting in the (100) and (101) directions. The observed preferred orientation for V_2_O_5_-sheets and V_2_O_5_-wires suggests that these particles may be more strained due to their nonthermodynamic distribution of surface facets.

### Characterization of PCET from Cr–H to V_2_O_5_

We next reacted these vanadium oxide samples with the strong hydrogen atom donor CpCr­(CO)_3_H and characterized the reduced solid. The goal in this section was to confirm that PCET is happening by identifying new OH groups and reduced V species in the samples after reduction. The reaction of Cr–H with V_2_O_5_ in acetonitrile causes the solid to change color from yellow to dark blue, indicative of the reduction of V^5+^ to V^4+^. This change is accompanied by the conversion of Cr–H into [CpCr­(CO)_3_]_2_, which can be observed by *in situ* IR spectroscopy (ReactIR) of the slurry (Figure 5). If we do this reaction under 13 bar H_2_ at room temperature, the PCET to V_2_O_5_ becomes catalytic in Cr–H (eq 1). This is convenient as it allows reduction of V_2_O_5_ via the activation of H_2_ by [CpCr­(CO)_3_]_2_ to reform the corresponding Cr–H.^47,48^ However, it is difficult to control as the extent of the PCET reaction (degree of reduction) will likely be dependent on the hydrogen pressure in the vessel.

1 $${xH}_{2}+V_{2}O_{5}\overset{{CH}_{3}CN,r.t.}{\underset{Cr-H}{\to }}V_{2}O_{5-2x}{\left(OH\right)}_{2x}$$

**Figure 3:**
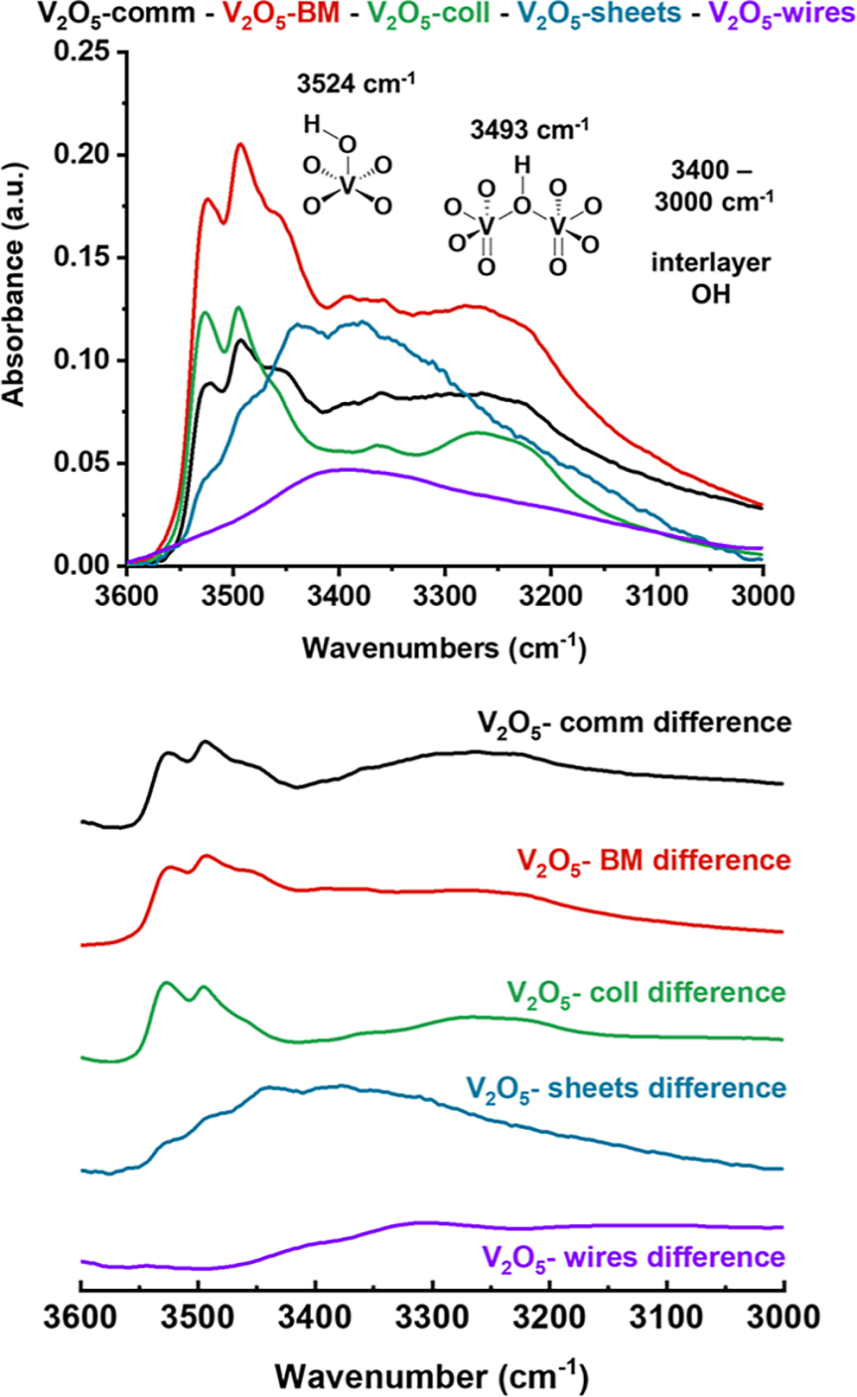
FTIR spectra of different V_2_O_5_ samples after reduction with Cr–H (top) as well as difference spectra (after–before)­(bottom) showing newly formed OH groups at 3524 cm^–1^, 3493 cm^–1^ and a hydrogen bonded interlayer OH band 3400–3000 cm^–1^.^51^ IR spectra before PCET are shown in Figure S8.

**Figure 4:**
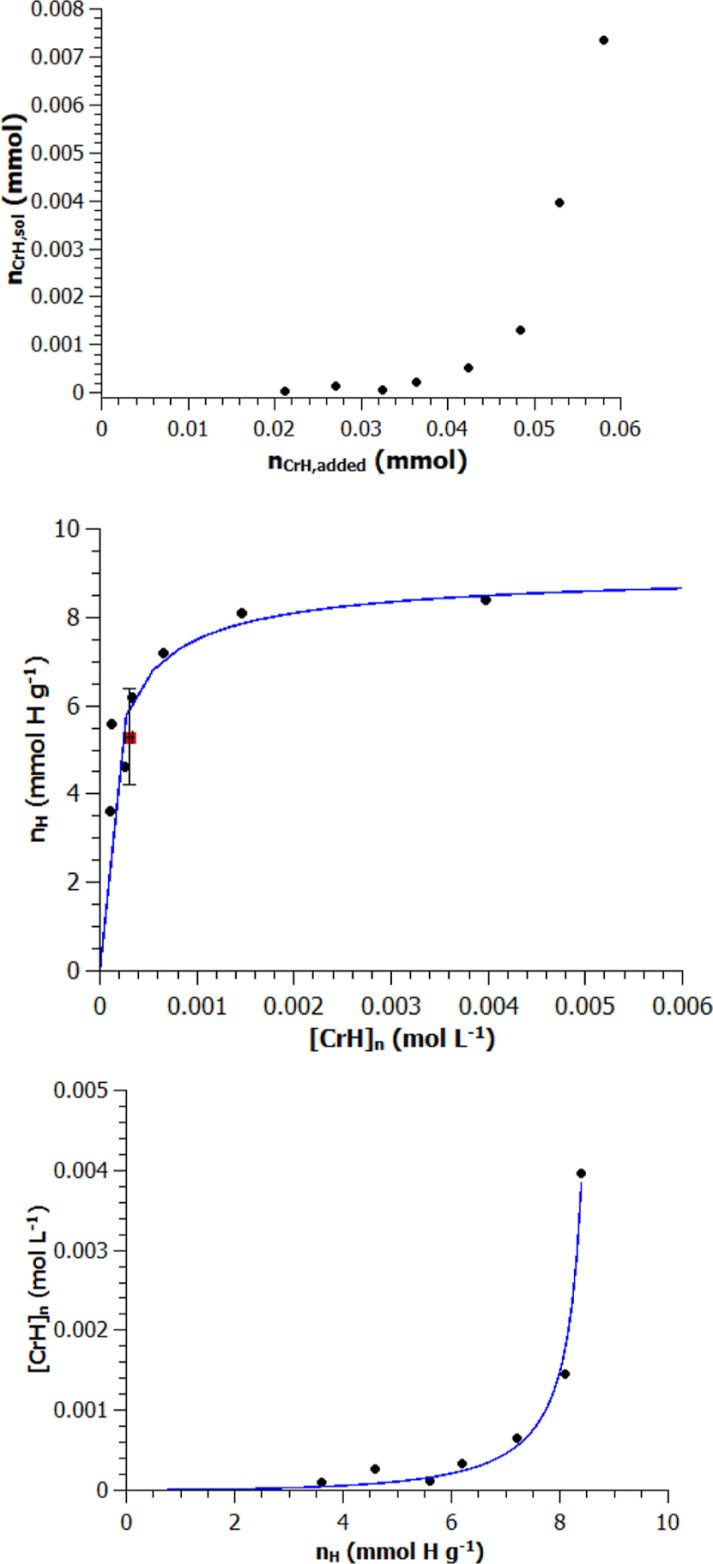
Amount of CrH remaining in solution after reaction with V_2_O_5_-coll vs total amount of CrH added (top) Concentration profile for the titration of V_2_O_5_-coll with Cr–H fitted to both the Langmuir–Freundlich (middle) and Frumkin isotherm (bottom). Red point in the middle graph represents the equilibrium achieved during the reverse PCET reaction from V_2_O_5_ to [CpCr­(CO)_3_]_2_. Blue lines are the respective isotherm fits.

**Figure 5:**
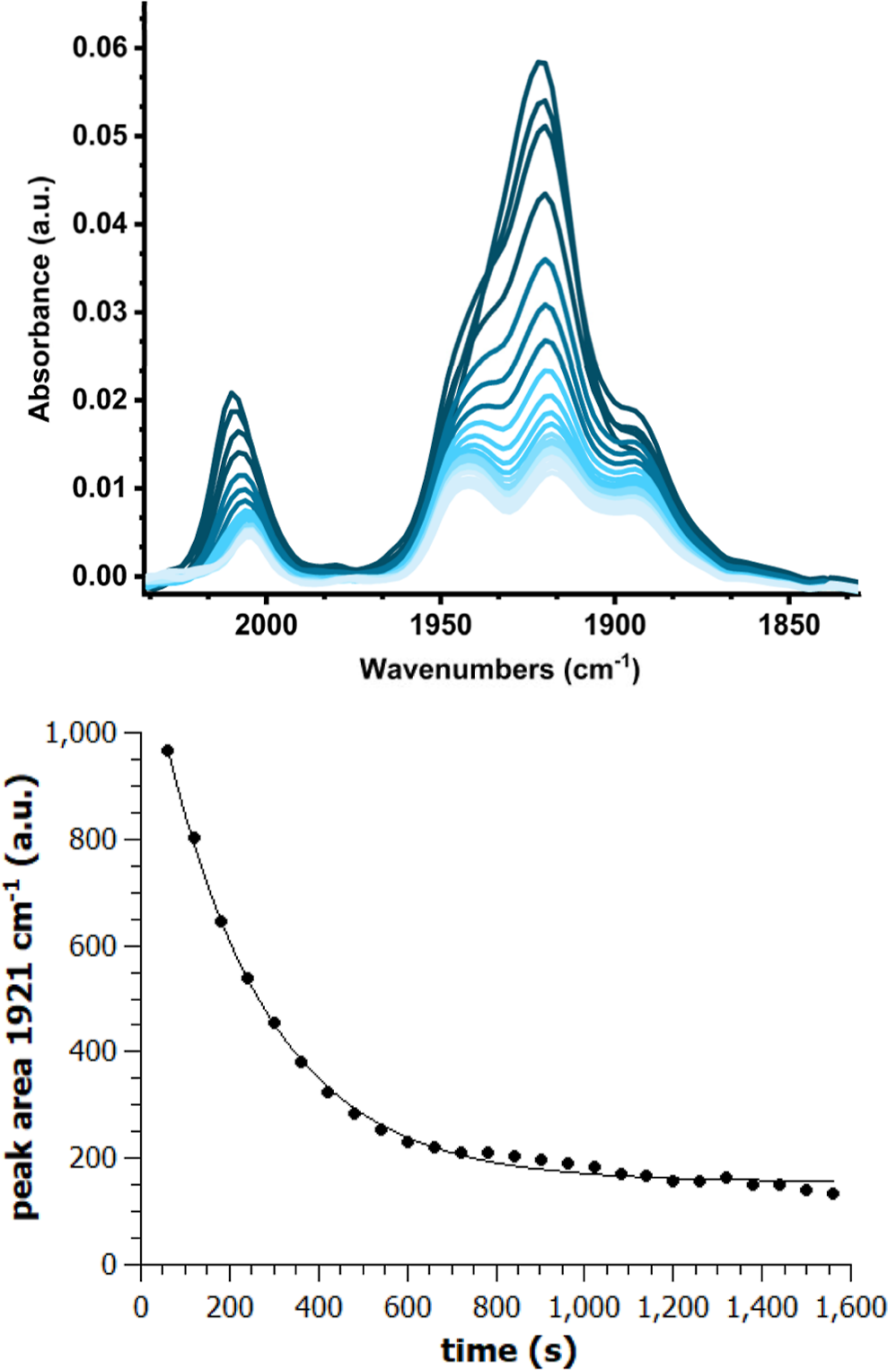
Representative reactIR spectra (top) and concentration profiles of the carbonyl region of Cr–H vs time during the PICET to V_2_O_5_-coll with 1st order fit (bottom).

These reduced V_2_O_5_ samples contain both new OH groups and V^4+^ centers as confirmed by IR, XRD, and X-ray absorption spectroscopies. The IR spectra of both the as-synthesized and reduced V_2_O_5_-comm samples are shown below in Figure 3. After reduction, three new OH bands appear at 3524, 3493, and one broad signal at 3400–3000 cm^–1^. This confirms that new OH groups are formed upon reaction of Cr–H with V_2_O_5_. In addition to the new OH peaks, we also observe weak bands due to CpCr­(CO)_3_ fragments physisorbed on the surface, which we also observed during metal hydride reductions of both MoO_3_ and CeO_2_ previously.^41,42^ The observation of V–OH groups on V_2_O_5_ surfaces in the literature is relatively rare, due to facile dehydroxylation/water loss. However, by comparison of the newly formed V–OH bands to literature reports, we can assign these peaks to plausible OH groups. DFT calculations showed that hydrogen binds to V_2_O_5_ [001] (the most abundant surface) at both the vanadyl oxygen and at a μ^2^ bridging oxygen position with nearly the same energy, with the terminal V–OH being ca. 30 cm^–1^ higher than bridging V–OH–V.^49^ This matches our observed bands at 3524 cm^–1^ and 3493 cm^–1^, meaning these could be due to both terminal and bridging OH groups on the [001] surface, respectively. Irradiation of V_2_O_5_ samples in the presence of ethanol results in PCET to form a new OH peak at ∼3300 cm^–1^,^50^ consistent with the broad band at 3400–3000 cm^–1^ in our spectrum. This broad peak indeed accounts for most of the new OH groups on the surface and can either be attributed to chemisorbed water or hydrogen bonded V–OH groups in the bulk oxide (interstitial OH groups). These materials were both stored under Ar and in several cases harshly calcined (V_2_O_5_-comm and V_2_O_5_-sheets at 500 °C), suggesting that the as synthesized materials had very little water in the interlayers. However, physisorbed water could either be formed during the PCET reactions or small amounts leached out of the acetonitrile. To check for trace water being absorbed into the V_2_O_5_ samples, we stirred V_2_O_5_-wires in acetonitrile for 1h (without CrH or H_2_) and found almost no difference in the IR spectrum (Figure S8). This would suggest that any water in the interlayer is most likely formed during PCET.

However, this broad OH band may also be due to interstitial OH groups that hydrogen bond with each other in the interlayer space, leading to a broad O–H stretching band at lower frequency. Similar effects have been observed for hydrogen bonded Si–OH bands of defective (siliceous) zeolites^52,53^ and in layered silicates.^54^ In reality, both interstitial OH groups and physisorbed water are most likely present inside the bulk material. We confirmed this by measuring XRD of V_2_O_5_-comm and V_2_O_5_-wires of the reduced samples after reduction (Figure S9). Both samples show the original reflexes due to V_2_O_5_ (albeit with much broader and slightly shifted peaks, due to the variation of V–O bond lengths upon reduction). In addition, both samples show new phases corresponding to water formation and incorporation in the lattice. In the case of V_2_O_5_-comm, this takes the form of a phase (H_3_O)_2_V_3_O_8_ consisting of layers of VO_5_ pyramids (33% V^4+^) separated by a layer of hydrogen-bonded interstitial H_3_O^+^ groups.^55^ On the other hand, for V_2_O_5_-wires the peaks are much broader than in V_2_O_5_-comm and the only identifiable new phase is a V_2_O_5_ xerogel hydrate,^56^ suggesting that some of the OH groups have formed water. Both of these results confirm our suspicions from IR spectroscopy, that some of the hydrogen atoms incorporated into V_2_O_5_ are present as intralayer OH groups while some form H_2_O molecules that fill the interlayer spaces of V_2_O_5_.

We also observed the formation of reduced V centers both qualitatively and quantitatively using V K-edge X-ray absorption spectroscopy (spectra shown in Figure S9). The results of the XANES analysis are shown in Table S2. Comparing the edge energies of both the unreduced and reduced samples to oxidation state standards for V^5+^ (high purity V_2_O_5_), V^4+^ (VO­(SO_4_)·4H_2_O), and V^3+^ (V_2_O_3_) demonstrates that the V centers in V_2_O_5_ are partially reduced upon treatment with Cr–H. The edge energies of the fully reduced samples (defined here as the energy corresponding to half the step-edge intensity) vary between 5480.6 and 5478.2 eV. The presence of V^4+^ centers can be observed and quantified based on an increased intensity at the top of the absorption edge at 5488 eV, the normalized absorption of which increases linearly upon reduction from V^5+^ to V^3+^ (Figure S10).^57^ Quantification of the average V-oxidation state for the samples reduced catalytically with Cr–H under 13 bar H_2_ for 24 h at room temperature shows that all of the samples have average oxidation states between 4.7 and 4.4, with V_2_O_5_-sheets being the least reduced after this reaction time and V_2_O_5_-comm/V_2_O_5_–BM both having the lowest average oxidation state. The average V-oxidation state achieved during these reductions under H_2_ is likely related to H_2_ pressure in a rather complex way, making these results more qualitatively useful than quantitative. Despite this warning, we can say that, given their low surface areas (between 4 and 42 m^2^ g^–1^) and that the most abundant [001] surface contains 5.76 V atoms nm^–2^, only between 0.4 and 4% of the total V atoms are on the surfaces of these samples. Thus, most of the H· must be transferred to the bulk of the material via a PICET reaction. This matches the observation of hydrogen bonded interlayer OH groups in the IR spectra. This is also similar to what we previously determined for MoO_3_, in which the hydrogen atoms can diffuse into the bulk along the MoO_3_ layers via hydrogen bonding pathways.^58^ This is also similar to the transfer of surface H atoms into the bulk, which was recently directly observed for WO_3_ by operando APXPS.^59^ Thus, we could prove that the reaction of CrH with V_2_O_5_ results in PICET to give new V^4+^–OH groups both on the surface and in the bulk of the material.

### Titration of V_2_O_5_ Samples with CpCr­(CO)_3_H

We next titrated the V_2_O_5_ materials with CpCr­(CO)_3_H in order to measure both the amount of PICET that occurs to each sample and, where possible, measure the thermodynamics of PICET from CrH to V_2_O_5_ (eq 2), similar to our previous work with CeO_2_.^42^ The results are shown in Figure 4 and Table 1 (full results in Figures S12–S19). This was done by adding aliquots of Cr–H (*n*_CrH, added_) to a known mass of V_2_O_5_ and measuring the amount of Cr–H left in solution after the reaction reached a stable end point (*n*_CrH, sol_) by ^1^H NMR (example spectra in Figures S12 and S13). The difference between *n*_CrH, added_ and *n*_CrH, sol_ divided by the mass of V_2_O_5_ represents the amount of PICET that has occurred to V_2_O_5_ per gram of material after each addition (denoted *n*_H_ with units of mmol H g_V2O5_^–1^). The results are shown in Figure 4 as well as in Figures S15–S19, displayed as *n*_CrH, sol_ vs *n*_CrH, added_. After addition of the first aliquots of CrH, *n*_CrH, sol_ is small but measurable, indicating that most but not all of the CrH in the first aliquots reacts with V_2_O_5_. However, as more PCET occurs to V_2_O_5_, more CrH is left in solution, indicating that the reaction eventually reaches a saturation point. This saturation point (*q*_H_) represents the maximum amount of PCET possible from CrH to each V_2_O_5_ sample at high [CrH], and is not necessarily stoichiometric (*q*_H_ = 11 mmol H g^–1^ corresponds to reduction of all V atoms to V^4+^). This saturation behavior can been visualized by plotting *n*_H_ vs the concentration of CrH in the solution at the end point of each aliquot addition (denoted [CrH]_*n*_).

These curves show a steep rise at low [CrH]_*n*_, followed by a flattening that asymptotically approaches *q*_H_ at infinitely high [CrH]_*n*_, characteristic of chemisorption reactions. We tested the reversibility of these reactions by treating prereduced V_2_O_5_ samples with [CpCr­(CO)_3_]_2_ (the reverse of eq 2). This resulted in back transfer of H· from reduced V_2_O_5_ to the Cr atom, thereby regenerating the Cr–H, which was quantified by ^1^H NMR (Figures S14 and Table S3). With the exception of V_2_O_5_-wires, the values of [CrH]_n_ and *n*_H_ achieved during the reverse PCET reactions (denoted in Figure 4 by a red data point) were within error of those measured for the forward reaction, suggesting that thermodynamic equilibrium had been achieved in these cases. The disagreement between the forward and reverse PCET reactions for V_2_O_5_-wires suggests that this is not the case, and any estimates of the thermodynamics of PCET for this sample represent lower limits.

We modeled titration data using adsorption isotherms to estimate both the thermodynamics and *q*_H_ for each sample. Unsurprisingly, none of the adsorption data followed a simple Langmuir isotherm, which was expected since the thermodynamics of PCET to metal oxides are known to vary as more PCET occurs.^36^ Therefore, we instead modeled this data using two isotherms that account for variable adsorption enthalpy as a function of coverage: namely the Langmuir–Freundlich and Frumkin isotherms (eqs 6 and 7). (For the purposes of our work, we modified these two isotherms to account for both the formation of CpCr­(CO)_3_ radical (eq 2) and its known dimerization equilibrium (eq 3).)^60^ The Frumkin isotherm especially has been shown to model both PCET reactions as well as intercalation reactions of ions in metal oxides well.^29,36,61^ We were able to fit the measured titration data to both isotherms with the same level of accuracy, giving estimates of both *q*_H_ and the thermodynamics of the PCET reaction (modeled as a *K*_eq_ and an inhomogeneity parameter). Both of these isotherms have complementary strengths and weaknesses. The Langmuir–Freundlich isotherm gives more accurate maximum loadings while the Frumkin isotherm provides a more straightforward interpretation of the reaction thermodynamics.^62^ The fitted parameters for each titration are listed in Tables 1 and S4.

Both isotherms gave the same *q*_H_ values (within error). The *q*_H_ values varied from 6.4(1.1) to 11(2) mmol H g^–1^ for V_2_O_5_-wires and V_2_O_5_-comm, respectively. This corresponds to reduction of between 58% and 100% of the V centers at high [CrH]. The *q*_H_ correlates with the inhomogeneity parameter g from the Frumkin isotherm, with samples with a lower *q*_H_ having a more negative *g* value. Aurbach and co-workers showed that for electrochemical intercalation reactions of layered metal oxides such as V_2_O_5_, the g value of the Frumkin isotherm was a function of how much structural rearrangement was necessary for continued intercalation of cations in metal oxides at higher degrees of reduction.^61^ They found that *g* > −4 indicated intercalation reactions involving minimal structural rearrangement, while g < −4 often required significant structural rearrangement (potentially even phase transitions) in order to be further reduced past a certain point. Since our reactions occur at room temperature and such structural changes are often accompanied by a large thermal barrier, samples with a more negative g value could end kinetically at the point where a phase transition would be necessary. In our case, the fact that V_2_O_5_-wires has the lowest *q*_H_, a *g* value < −4, and that it failed the reversibility test may mean that PICET to V_2_O_5_-wires is kinetically limited due to a structural transition that cannot occur at room temperature. The same could be true of V_2_O_5_-sheets, which also had a low *q*_H_ and *g* < −4, suggesting that PICET to V_2_O_5_-sheets may only be quasi-reversible due to a similar necessary structural rearrangement. Interestingly, the two samples with the lowest g-values are also the two that have a preferred crystallite orientation. This nonequilibrium particle morphology may be related to the need for or difficulty of structural rearrangements during PCET. What exactly these structural variations are is not known at this time, but could be related to the xerogel formation that we saw in the XRD of the reduced V_2_O_5_-wires above.

$$1/x\left[\begin{array}{l} V_{2}O_{5}\\ +\\ xCpCr{\left(CO\right)}_{3}H_{\left(ac\right)} \end{array}\overset{\Delta G_{eq}}{⇌}\begin{array}{l} V_{2}O_{5-x}({OH)}_{x}\\ +\\ \frac{x}{2}{\left[CpCr{\left(CO\right)}_{3}\right]}_{2(ac)} \end{array}\right]$$ 2

$$1/2{\left[CpCr{\left(CO\right)}_{3}\right]}_{2\left(ac\right)}\overset{-1/2\Delta G_{\dim}}{⇌}CpCr{\left(CO\right)}_{3\left(ac\right)}$$ 3

$$CpCr{\left(CO\right)}_{3\left(ac\right)}+H_{\left(ac\right)}\overset{-BDFE\left(CrH\right)}{⇌}CpCr{\left(CO\right)}_{3}H_{\left(ac\right)}$$ 4

$$1/xV_{2}O_{5}+H_{\left(ac\right)}\overset{-BDFE\left(OH\right)}{⇌}1/xV_{2}O_{5-x}{\left(OH\right)}_{x}$$ 5

6 $$n_{H}=\frac{q_{H}a^{b}{\left[CrH\right]}^{b}}{\sqrt{\frac{{\left[Cr\right]}_{tot}}{2K_{Cr}}}+a^{b}{\left[CrH\right]}^{b}}$$

7 $${\left[CrH\right]}_{n}=\frac{n_{H}\sqrt{\frac{{\left[Cr\right]}_{tot}}{2K_{Cr}}}}{K_{eq}\left(q_{H}-n_{H}\right)}e^{-gn_{H}/q_{H}}$$

We can calculate the free energy of reaction 2 using the *K*_eq_ values from the Frumkin isotherm fitting. By scaling this free energy to the number of H atoms transferred and subtracting the free energies of eqs 3 and 4 (CpCr­(CO)_3_ dimerization and BDFE of the Cr–H bond), we can calculate the BDFE of the V^4+^–OH groups in the material at the point of saturation (at *q*_H_). These BDFE values and the differences between samples (ΔBDFE) are shown in Table 1. The values range from as low as 55.7 up to >63.3 kcal mol^–1^, with less negative *g* values corresponding to lower BDFE­(OH). V_2_O_5_-BM having the lowest BDFE and V_2_O_5_-wires having the highest BDFE. This further supports our conclusion from above that PICET to V_2_O_5_-wires and potentially V_2_O_5_-sheets are limited by a structural transition, since Cr–H (BDFE = 57 kcal mol^–1^)^63^ should be capable of reducing V_2_O_5_ even further, in the absence of kinetic limitations.

Our results compare well with previous thermodynamic measurements from the literature. In particular, we see that as *q*_H_ increases, BDFE­(OH) also goes down. Dickens and co-workers measured the free energies of hydrogenation of V_2_O_5_ via calorimetry as a function of the degree of reduction (*x*_avg_).^64^ Using their data, we could estimate that the BDFE­(OH) values (Table S5) go from 68(3) kcal mol^–1^ at *x*_avg_ = 0.055 down to as low as 56(4) kcal mol^–1^ at *x*_avg_ = 1.82 (which was accompanied by a phase change, as we predicted would be the case for V_2_O_5_-wires and V_2_O_5_-sheets above). Matson and co-workers measured V^4+^–OH BDFEs for small vanadium polyoxometalate (POM) clusters at different loadings and also found that the BDFE­(OH) values fall from 65.3 kcal mol^–1^ down to as low as 61 kcal mol^–1^ as more H· is transferred to the POM.^30,33^ Ponnusamy and co-workers estimated that the first PCET reactions to unreduced vanadium centers may have BDFEs as high as 72.6 kcal mol^–1^ during C–H-oxidation reactions using tungsten POMs with dimeric vanadate sites.^65^ The comparison of our work with others shows that the thermodynamics of PCET do vary with *n*_H_, with BDFEs varying at least 8 kcal mol^–1^ and potentially as much as 17 kcal mol^–1^. Our results also suggest that PICET to particles with structures that deviate from the expected thermodynamic morphology could also require structural rearrangements during PICET.

### Effect of Particle Morphology on the Kinetics of PCET

We next measured the kinetics of PICET from Cr–H to V_2_O_5_ by ReactIR of the carbonyl region of the reaction slurry in CH_3_CN. Kinetics were measured using a small amount of CrH amounting to reduction of <10% of V centers (below *n*_H_ = 1.1 mmol g^–1^) in order to minimize variations in the reaction thermodynamics over the course of the reaction. We measured the reaction rates by spectral deconvolution of the overlapping peaks of CpCr­(CO)_3_H and [CpCr­(CO)_3_]_2_, using the iC IR software package. The reaction progress could be monitored most accurately by following the deconvoluted peak areas of the CrH peak at 1921 cm^–1^ as a function of time. Example curves and spectra are shown in Figure 5 while the full data are shown in S20–S36. The reaction profiles showed a first oder dependence on CrH and were also sensitive to the mass of V_2_O_5_ used in each run (for example, increasing the mass of V_2_O_5_-sheets resulted in a corresponding increase in the reaction rate). The rate constants for all the samples were within error of each other at 0.04–0.05 g^–1^ s^–1^, with the exception of V_2_O_5_-sheets, which was about an order of magnitude slower at 0.005(3) g^–1^ s^–1^. There is no indication that the kinetics are dependent on the surface area, with four of the five samples having the same PICET rate constant but very different surface areas (4–42 m^2^ g^–1^). In addition, the only material whose rate is different is V_2_O_5_-sheets, which reacts slower than the other samples despite having the second highest surface area.

PICET reactions consist of several different steps that make them distinct from other PCET mechanisms, one of which is the diffusion of H· into the bulk material.^29,39^ It is possible that the rate of PICET from CrH to V_2_O_5_ is controlled not by the rate of PCET at the surface (eq 8) but instead by this diffusion of H· away from the surface (eq 9). This would indeed explain why four of the five materials undergo PICET at very similar rates. This situation can be described kinetically by the mechanism shown in eqs 8 and 9 consisting of a reversible PCET from CrH to the V_2_O_5_ surface followed by transport of the H· into the bulk. Assuming that the rate of H· diffusion is much slower than that of the transfer of H· back to CpCr­(CO)_3_ gives a rate law that is first order in CrH but is not dependent on the surface area of V_2_O_5_. (see the derivation of the rate law equation S3 in the Supporting Information). This suggests a situation in which the surface is saturated with H· such that a subsequent PCET event cannot occur until the previous proton–electron pair has been transported into the bulk of the material, thereby freeing up a surface site for further PCET. This would make it possible for the initial transfer to the surface to be reversible before the rate determining surface-to-bulk PCET. Such a mechanism has also been proposed for PCET to MoO_3_.^66^

8 $$Cr-H+V_{2}O_{5}\underset{k_{-1}}{\overset{k_{1}}{⇌}}H^{*}/V_{2}O_{5}+1/2_{2}{Cr}_{2}$$

9 $$H^{*}/V_{2}O_{5}\overset{k_{diff}}{\to }V_{2}O_{5-2x}{\left(OH\right)}_{2x}$$

There are a variety of potential explanations for the difference in the rate of H· diffusion between V_2_O_5_-sheets and the other samples. These include (1) varying particle morphologies/surface facets, (2) structural vacancies, or (3) varying amounts of grain boundaries/crystal defects. There is no obvious connection between the rate of PICET and the proportion of defect sites such as oxygen vacancies, as V_2_O_5_-comm and V_2_O_5_-sheets both have similar proportions of oxygen vacancies (Table S1) but a 10-fold difference in rate. We also have no indication from HRTEM that individual samples have especially different amounts of grain boundaries/crystal defects (although we cannot explicitly rule this out). Surface faceting/particle morphology could, however, play a role in the variation of rates of the PICET reactions. H· diffusion into the bulk of layered materials, such as MoO_3_ and V_2_O_5_, is often anisotropic (significantly faster along the material layers than perpendicularly across the interlayer space).^67–69^ The layers of V_2_O_5_ follow strands travelling along the (010) direction, where the VO_5_ square pyramids connect via corners to form a well-bonded layer in the [110] plane. These layers are only weakly connected along the (001) direction, yielding the laminar structure seen in Figure 6. Consequently, diffusion between VO_5_ units along the layers (i.e., along the (100) direction) is fast while diffusion perpendicular to the layers along (001) and indeed any direction other than (100) and (010) is slow. This would mean that adsorption on the [001] surface would result in only surface OH groups but that hydrogen atoms adsorbed on any [hk0] facet and especially the [100] and [010] facets can diffuse into the bulk easily, even at room temperature. Therefore, vanadium oxide samples with a higher proportion of these latter surfaces exposed to the reaction medium should have higher rates for their PICET reactions. Based on the results of our Rietveld refinement, all of these samples should have relatively similar amounts of the [hk0] facets exposed, except for V_2_O_5_-sheets, which almost exclusively exposes the [001] surface. This leaves fewer entrances for the added hydrogen atoms to diffuse in between the layers, and consequently shows the slowest rate of PICET. V_2_O_5_-wires also has a preferred orientation of [h0l] facets. However, despite the presence of [001] and [101] surfaces, the large portion of [100] facets are able to facilitate rapid PICET in V_2_O_5_-wires. The latter facet still allows fast diffusion of the protons into the bulk, even though the [010] facet, along which direction the V_2_O_5_-wires grow, is underrepresented. Therefore, V_2_O_5_ samples with surfaces consisting almost entirely of the [001] facet can be expected to undergo slower PICET than nanoparticles with no preferred crystallite orientation or high proportions of either [100] or [010] surfaces.

**Figure 6:**
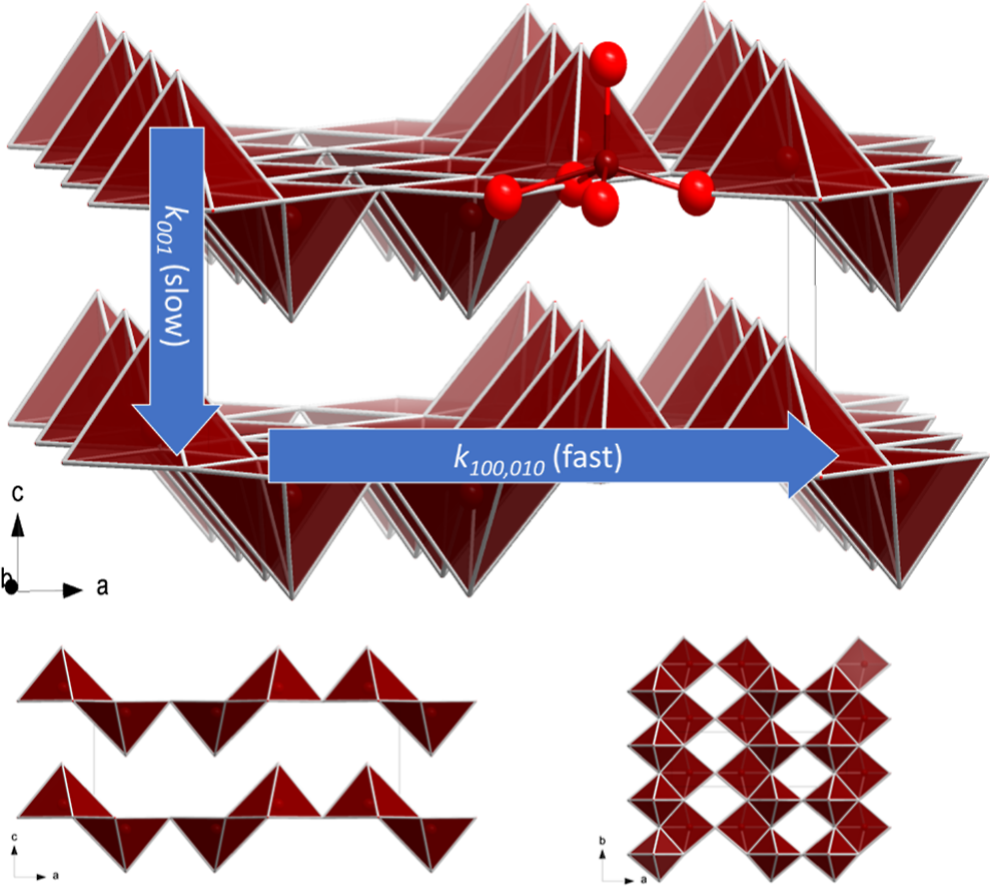
Anisotropic diffusion of H· through the crystal structure of V_2_O_5_ with one polyhedra being removed to illustrate the pyramidal coordination of vanadium (burgundy) by oxygen (red). The view along [010] (left) and [001] (right) (the *b*- and *c*-axes, respectively) are shown at the bottom.

### Relevance of PICET to Oxidation Catalysis with V_2_O_5_

We can use the variations in the PICET reactivity of the samples measured here to understand differences in V_2_O_5_-catalyzed oxidation reactions that involve PCET. Here, we investigated the V_2_O_5_-catalyzed oxidation of methanol to formaldehyde in a gas-phase flow reactor at 300 °C and 20 mL min^–1^ flow rate of a mixture of methanol vapor in excess synthetic air. The conversions and selectivities at 300 °C as well as the selectivities at 50% conversion are shown in Table 2. Conversions and formaldehyde selectivities at 300 °C are plotted in Figure 7 as a function of the surface area of the catalyst. Indeed, the conversion of methanol increases logarithmically as a function of the V_2_O_5_ surface area across the samples, accompanied by the expected lower formaldehyde selectivity at higher conversions. This is what would be expected for a structure insensitive reaction (where the reaction rate of methanol conversion is the same on all surface facets). The only exception to this is V_2_O_5_-sheets, which shows very low conversion of only 11% (rather than the 98% conversion expected from its surface area). In comparison, V_2_O_5_-comm reaches 50% conversion at the same conditions but has a surface area that is ca. three-times smaller than V_2_O_5_-sheets. The catalytic activity of the various samples therefore follows the same trend as seen for the kinetics of PICET, suggesting that H· from methanol diffuses into the bulk V_2_O_5_ during catalysis. In support of this, during the catalytic reaction the V_2_O_5_ samples all changed color from orange to dark blue, indicating that bulk V atoms are reduced during catalysis.

**Table 2:** Catalytic Activity and Selectivity of the V_2_O_5_ Catalyzed Selective Oxidation of Methanol to Formaldehyde

sample	% conv. at 300 °C	% sel._CH2O_ at 300 °C	*T*_50%_ (°C)^a^	% sel._CH2O_ b
V_2_O_5_-comm	51	96	300	96
V_2_O_5_-BM	98	71	250	88
V_2_O_5_-coll	75	81	270	84
V_2_O_5_-sheets	11	94	330	89
V_2_O_5_-wires	98	60	245	85

a Temperature at which 50% conversion was achieved.

b Formaldehyde selectivity at 50% conversion.

**Figure 7:**
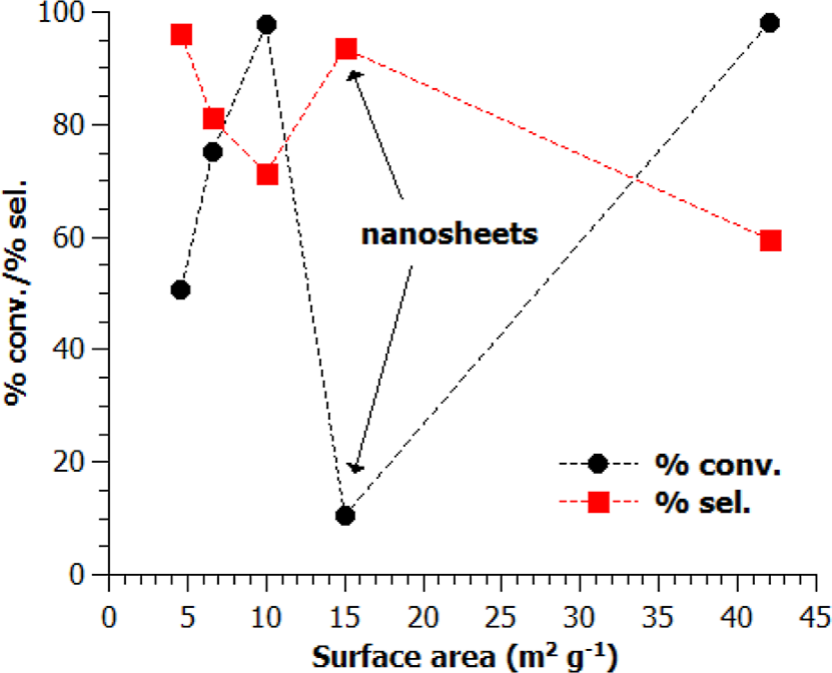
Relationship between the % conversion and % formaldehyde selectivity of methanol oxidation at 300 °C and the V_2_O_5_ surface area demonstrating the low performance of V_2_O_5_ nanosheets. (catalysis measured at 20 mLn min^–1^ flow rate of a synthetic air/methanol mixture).

The discrepancy between the catalytic activity of V_2_O_5_-sheets and the other samples is potentially due to the involvement of PICET in the catalytic mechanism. This is supported not only by our own observations but by other reports of V_2_O_5_ catalyzed alcohol oxidation in the literature. Gasior and Machej^70^ showed that nonsurface V atoms in the bulk of V_2_O_5_ particles are reduced to V^4+^ during the oxidation of o-xylene to phthalic anhydride, suggesting PICET is happening in these cases. Baiker and Monti also studied the effect of particle morphology on the V_2_O_5_ catalyzed partial oxidation of methanol to formaldehyde and observed a similar particle morphology dependence as we do.^71^ Their experiments compared two samples, one with a plate-like structure exposing mostly the [001] surface and one consisting of needle-like particles with significant contributions from the [110] surface. From the conversion data they present, it can be estimated that the needle-like sample containing more [110] surface converts methanol to formaldehyde ca. three times faster than the plate-like nanoparticles, once one accounts for the different surface areas of the two samples (which were seemingly not considered in the original paper). However, Somorjai and co-workers showed that the rate of ethanol oxidation only depends on the surface area (i.e., is structure insensitive) in cases when such morphological/surface faceting differences are not present and only the V_2_O_5_ particle sizes vary.^7^ This is analogous to our observations, where V_2_O_5_-sheets have unusually low activity but all other samples have catalytic rates proportional to surface area.

Both our catalytic results and the literature on V_2_O_5_-catalyzed alcohol oxidation reactions suggest the adapted Mars-van-Krevelen mechanism in Figure 8. In this mechanism, methanol first binds to the surface followed by transfer of H from methoxy to bulk V_2_O_5_ as the rate determining step. This would then be followed by formaldehyde desorption, H_2_O loss to create an oxygen vacancy, then oxidation of this vacancy by O_2_ to reform the active site. This transfer could be a PICET reaction. The rate determining hydrogen transfer from bound methoxy to V_2_O_5_ may happen by one of several potential mechanisms. If this reaction were to occur by PCET from the methanol C–H bond (BDFE = 91 kcal mol^–1^)^72^ to V_2_O_5_ (BDFE­(V–OH) < 72.6 kcal mol^–1^ in the literature) it would be unfavorable by >18.4 kcal mol^–1^. While such a large activation barrier is accessible at these reaction temperatures (>250 °C), the rate of the back reaction (PCET from V^4+^–OH back to the bound formaldehyde) will likely be very fast. However, if the hydrogen atom is transported away from the surface into the bulk via a PICET reaction, then the back transfer becomes much slower, giving formaldehyde time to desorb from the surface before back transfer can occur. Other researchers have proposed that instead of by PCET, the selective oxidative of alcohols on V-based catalysts occurs via a β-H elimination from the alkoxy intermediate.^73,74^ In such a β-H elimination mechanism, V^4+^–OH groups are also created on the surface (after tautomerization of an intermediate V–H group)^75,76^ and could also potentially transfer the hydrogen back to bound formaldehyde before its desorption. Therefore, both mechanisms could potentially benefit from diffusion of the hydrogen atoms away from the V_2_O_5_ surface. The idea of hydrogen diffusion into the bulk being important for C–H-oxidation on a heterogeneous catalyst is in some ways reminiscent of the well-known role of solvent cage escape during radical, biocatalytic, and photochemical reactions in solution.^77–79^

**Figure 8:**
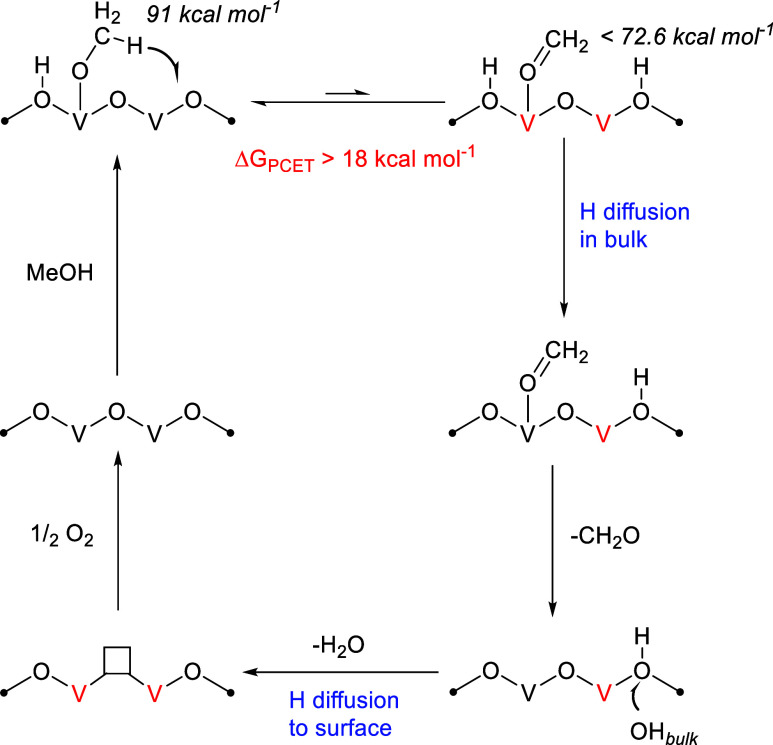
Potential mechanism of methanol oxidation by V_2_O_5_ involving PCET from methanol followed diffusion of H·into the bulk of the material. This same mechanism can be adapted for other surface terminations.

## Conclusions

Here, we carried out a systematic investigation of the effect of nanoparticle shape on the PCET reactivity of V_2_O_5_ by reacting the well-defined H· donor CpCr­(CO)_3_H with samples of V_2_O_5_ of varying morphology. The particle size and the surface facet distribution were measured using Rietveld refinement accounting for a preferred crystallite orientation. The reaction of CpCr­(CO)_3_H with V_2_O_5_ results in PICET to form new V^4+^ and OH sites that diffuse into the bulk of the metal oxide, as demonstrated by IR and V K-edge XANES. The thermodynamics of the PICET reaction were measured by titration of the various V_2_O_5_ samples with Cr–H. We fit these titration curves to chemisorption isotherms that account for adsorption enthalpies that vary with the extent of reduction. Fitting to the Frumkin isotherm demonstrated that it was possible to reduce some samples down to 100% V^4+^, while samples that had a nonequilibrium nanoparticle structure were kinetically limited by the need for structural rearrangements of V_2_O_5_ at higher *n*_H_. Calculation of the OH BDFE values of the H· loaded V_2_O_5_ samples showed that the BDFE­(OH) decreases at higher *n*_H_ values (down to a minimum value of 55.7 kcal mol^–1^). The kinetics of PICET to V_2_O_5_ are also dependent on nanoparticle structure. While most samples have the same rate constant of PICET, V_2_O_5_-sheets (whose surface consists almost exclusively of the [001] surface) undergoes PICET 10x more slowly. This is likely due to the fact that the diffusion of hydrogen atoms into the bulk of the material is much faster along the (hk0) directions (along the VO_5_ layers) than along the (001) direction (between the layers).

We measured the activity of these V_2_O_5_ samples for methanol oxidation to formaldehyde. The results showed that while the activity of most samples was structure insensitive (only proportional to the surface area), V_2_O_5_-sheets also had a much lower activity than expected based on surface area (11% conversion instead of the expected 98%). This coupled with the observation that V atoms in the bulk of the material are reduced to V^4+^ during catalytic oxidation suggests that PICET is involved in the mechanism of methanol oxidation. We proposed a mechanism in which H· diffusion into the bulk prevents the back transfer of H· to the intermediate hydroxymethyl radical, allowing them to continue reacting to form formaldehyde. Such a mechanism not only explains why catalysts with high proportions of [001] surface facets are much less active than expected but also explains how V^4+^ ions in the bulk material can be formed during an oxidation reaction. This mechanism is also reminiscent of other reactions from homogeneous and biocatalysis in which diffusion from solvent cages is an important step in directing the reactivity of radicals. This work demonstrates how bulk transformations such as PICET can still be involved in heterogeneous catalytic reactions that at face value only utilize metal sites on the nanoparticle surface. This work also demonstrates how much the thermodynamics and kinetics of reactions of metal oxides depend on the nanoparticle morphology, which is often not thoroughly characterized.

## Experimental Section

All reduction experiments with Cr–H were performed using standard Schlenk and glovebox technique under an Ar atmosphere (Ar 4.8, Westfalen AG). All kinetic and thermodynamic measurements were reproduced multiple times on multiple synthetic batches of material by multiple researchers, to ensure reproducibility. Solvents were purified by passage through activated alumina columns and sparging with Ar using an MBraun SPS. Anhydrous tetrahydrofuran (THF) was purchased from Sigma-Aldrich and stored under an Ar atmosphere. Deuterated acetonitrile was purified by freeze–pump–thaw degassing and dried over 4 Å molecular sieves. The commercial V_2_O_5_ (99.6+%) was purchased by Sigma-Aldrich (*S*_BET_ = 4.6 m^2^ g^–1^) and used as received. Precursors such as VO­(SO_4_)_2_ (99.9%) and NH_4_VO_3_ (+99.0%) were purchased from thermo scientific and Merck, respectively and used as received. Oxidizing agent KBrO_3_ (+99.0%) and reducing agent oxalic acid dihydrate (+99%) were purchased from VWR and used as received. Silica gel 60 M was purchased from Merck and used as received. All hydrothermal reactions were carried out in PFTE-lined 100 mL stainless-steel autoclave using bidistilled water. All catalytic reduction of V_2_O_5_ samples were carried out in a stainless-steel autoclave in a glass insert containing a stirring bar. Pressurized H_2_ (5.0) for reduction reaction was purchased from Westfalen AG.

Powder X-ray diffraction was measured on a Bruker D8 Advance powder diffractometer outfitted using a Cu Kα source and employing a Si-single crystal zero background sample holder. SEM measurements were carried out on a Tescan VEGA3 using samples sputtered with carbon. Ex situ liquid IR spectra were measured on a Nicolet 6700 spectrometer in transmission mode applying a liquid cell with CaF_2_ windows and 0.1 mm path length. IR spectra of solid samples were measured on a Bruker Alpha II IR spectrometer in an Ar glovebox in transmission mode using KBr pellets of the desired sample (ca. 1:10 weight ratio). Liquid-state ^1^H NMR spectra were measured on a 400 MHz Bruker Avance III spectrometer with a delay time of 30 s to ensure accurate integrals. ICP-OES of V was measured using a PerkinElmer Avio 200 Optical Emission Spectrometer calibrated to a standard V solution. BET surface areas were measured by N_2_ physisorption at 77 K using a Quantachrome Autosorb iQ gas manometry system. Samples were outgassed at 200 °C for 4–8 h prior to surface area measurements. Kinetic measurements of PICET reactions were measured using a Mettler-Toledo ReactIR 701L system with an IR fiberoptic connected to a diamond ATR dip-probe. Initial spectra were taken of the Cr–H solution before addition of V_2_O_5_ sample and then the probe replaced after addition of the solid followed by a ca. 6 min stabilization period after which spectra were measured every 1 min with a total of 157 scans. XANES measurement of all samples were conducted on an easyXAFS 300+ X-ray adsorption spectrometer at the V K-edge (5465 eV) using an X-ray source at with a Ag anode, a spherically bent Si(331) crystal analyzer, and a silicon drift detector from KETEK GmbH. Samples were pressed under argon into 13 mm pellets of V_2_O_5_ (ca. 5–10 mg) diluted in methyl cellulose (to a total mass of 50 mg) and XANES spectra measured using a step size of 0.25 eV and an exposure time per data point of 30 s. The edge energies were defined as the energy at which the intensity of the edge reaches 50%. XANES spectra were normalized in the same way for each spectrum for both the pre-edge and post edge corrections (pre-edge linear correction between 40 and 20 eV below *E*_0_, postedge correction by a cubic function between 30 and 500 eV above *E*_0_). Oxidation states were analyzed by comparison of the normalized intensity of the spectrum at 5488 eV versus that of known oxidation state standards for V^5+^ (high purity V_2_O_5_), V^4+^ (VO­(SO_4_)·4H_2_O), and V^3+^ (V_2_O_3_) as was previously reported by Lay and co-workers.^57^ Normalization and quantification of the spectra were carried out using the Athena program of the Demeter software package.^80^ All spectra were referenced against a standard V foil. HRTEM images were recorded using a ThermoFisher Spectra 300 at the SRF AMICA (Stuttgart Research Focus Advanced Materials Innovation and Characterization) center at the University of Stuttgart. The system was operated in STEM mode, at an acceleration voltage of 300 kV, a beam convergence angle of 10 or 22 rad, a camera length of 115 or 145 mm, and a beam current of 50–150 pA.

### Improved Synthesis of Cr–H

Cr–H was synthesized by protonation of Na­[CpCr­(CO)_3_][Bibr ref81] in THF via modification of a known literature procedure.^82^ Na­[CpCr­(CO)_3_] (2.1 mmol, 500 mg) was dissolved in THF (30 mL) and cooled in an ice bath. To this cooled mixture concentrated H_3_PO_4_ (0.5 mL) was added dropwise over 10 min, ensuring that the temperature of the solution did not rise enough to cause significant H_2_ loss. After the addition, the reaction was stirred for a further 45 min, then dried in vacuo in an ice bath (to prevent loss of the product in the vacuum line) and purified by sublimation onto an acetonitrile/dry ice cooled coldfinger. The purity was determined via ^1^H NMR spectroscopy versus mesitylene as internal standard (the residual unsublimed solid was confirmed by IR spectroscopy to be the dimeric complex [CpCr­(CO)_3_]_2_). Cr–H prepared by this method is a yellow solid (as opposed to the typical emerald green color indicative of impurities of [CpCr­(CO)_3_]_2_) and shows an unusually narrow Cp signal in the ^1^H NMR indicative of the very low amount of residual [CpCr­(CO)_3_]_2_ present in the sample. This was confirmed by solution phase IR spectroscopy, which showed that the sample contains essentially no detectable [CpCr­(CO)_3_]_2_.

### Preparation of V_2_O_5_-BM

The ball milling of V_2_O_5_-comm was conducted in a planetary ball mill (model PULVERISETTE 7 Fritsch GmbH). Neat V_2_O_5_-comm (1.6 g) and 10 mm zirconia grinding balls (16 g) were added to a zirconia milling bowl (45 mL) with BPMR = 10:1. The ball milling was performed at room temperature in an enclosed air atmosphere with a disk speed of 300 rpm. The powder was dry milled 1 min and then paused for 1 min to reduce heat accumulation in the system. This procedure was repeated five times, leading to a total milling time of 5 min. The orange powder was stored in a glovebox under Ar atmosphere for further use and characterization.

### Synthesis of V_2_O_5_-Coll

Pale-yellow NH_4_VO_3_ (55 mmol, 6.44g) was added to 300 mL of bidistilled water while stirring at room temperature until it turned red. Due to poor solvation of the metavanadate, the mixture was further stirred until no change was observed, then oxalic acid dihydrate (87 mmol, 11.0 g) was added slowly until all residue was dissolved, resulting in a yellow solution. The solution was then heated to 80 °C and kept at the same temperature for 60 min while constantly stirring during which time the vanadium solution turned a dark blue color. The mixture was transferred to an oven at 95 °C for 48 h until the water was evaporated and the dark solid fully dried. The resulting product was crushed using a mortar and pestle, then the resulting powder was finally calcined at 500 °C for 12 h (heating ramp 2 °C min^–1^) with a synthetic air flow of 100 L min^–1^. The orange V_2_O_5_ powder (4.8 g) was stored in a glovebox under Ar atmosphere for further use and characterization.

### Synthesis of V_2_O_5_-Sheets

A suspension of V_2_O_5_ (27.5 mmol, 5.0 g) in 300 mL bidistilled water was stirred for 20 min at room temperature. 200 mL of 30% aqueous H_2_O_2_ solution (2.56 M) was then added to this mixture dropwise. The color of the suspension changed from yellow to a clear orange accompanied by gas formation and then finally turned dark brown after stirring for 2.5 h at room temperature. The mixture was stirred and heated to 60 °C for 3 h, dried in an oven at 95 °C for 48 h, and subsequently crushed into a powder and calcined at 400 °C for 2 h (heating ramp 1 °C min^–1^) with a synthetic air flow of 50 L min^–1^. The orange-brown product (4.5 g) was crushed again, characterized, and stored in a glovebox under Ar atmosphere for further use.

### Synthesis of V_2_O_5_-Wires

V_2_O_5_-wires were synthesized by hydrothermal synthesis in 100 mL PTFE lined stainless steel autoclaves. Since the literature procedure only yielded low amounts of product, we quadrupled the scale. Stock solutions of VO­(SO_4_)_2_ (73.6 mmol, 12.0 g) in 80 mL bidistilled water and KBrO_3_ (40.0 mmol, 6.7 g) in 160 mL didistilled water were prepared. The KBrO_3_ solution was added dropwise to the blue VO­(SO_4_)_2_ solution under stirring at room temperature. The mixture was stirred for an additional 45 min resulting in a color change from blue to green and finally orange, forming small amounts of an orange precipitate. Concentrated HNO_3_ (36 mL) was then added dropwise (over 30 min) to dissolve the orange precipitate, resulting in a transparent yellow solution. This reaction mixture was transferred into four separate 100 mL PTFE-lined stainless-steel autoclaves and heated to 190 °C for 24 h in an oven and then cooled down to room temperature. The resulting mixtures were combined, filtered, and washed with three aliquots of bidistilled water (20 mL) and three aliquots of ethanol (20 mL). The orange solid (1 g) was dried at 80 °C for 16 h, characterized, and stored in a glovebox under Ar atmosphere for further use.

### General Procedure for PCET from Cr–H to V_2_O_5_

Five mL of a 0.02 mM solution of Cr–H in CH_3_CN was added under Ar to 199 mg of V_2_O_5_ and stirred vigorously for 3 h. During the reduction of V_2_O_5_, the orange powder turned dark blue. Initial samples (ca. 0.2 mL) of the solution were taken after 5, 30, 60, and 180 min and measured via IR (Figure S14). Upon PCET to V_2_O_5_ the characteristic CO signals of Cr–H at 2011 cm^–1^ and 1920 cm^–1^ decrease and disappear with concomitant appearance of the CO bands 1945 cm^–1^ and 1893 cm^–1^ indicative of the dimeric [CpCr­(CO)_3_]_2_. After the reaction, the powder was filtered and washed with 4 × 3 mL of acetonitrile, dried under vacuum and stored under Ar.

### Catalytic PCET from Cr–H to V_2_O_5_ under H_2_

A stainless-steel autoclave was charged with 2.0 mL of acetonitrile, V_2_O_5_ (0.55 mmol, 100 mg), and 10 mol % Cr–H (0.055 mmol, 11.1 mg) in an Ar glovebox. The autoclave was then pressurized at room temperature with 13 bar H_2_ and the mixture stirred (300 rpm) at room temperature for 24 h. After this time, the autoclave was depressurized and flushed three times with N_2_ to remove excess H_2_ in the system and then transferred into an Ar glovebox. The dark green reaction solution was stored for further characterization and the darkly colored reduced V_2_O_5_ samples were washed 5 to 7 times with *n*-pentane until the liquid was colorless. The resulting solid was allowed to dry overnight in vacuo and stored under Ar.

### Titration of V_2_O_5_-Comm with Cr–H

Titration of V_2_O_5_ samples with Cr–H was carried out as follows. First, a stock solution of Cr–H (0.01 mmol, 20.0 mg) in CD_3_CN (2 mL) was made and then the exact concentration was calculated by ^1^H NMR by transferring 0.4 mL of the stock solution into a J Young NMR tube under argon along with mesitylene as an internal standard. Then the V_2_O_5_ (0.03 mmol, ca. 5.0 mg) was added, and the mixture was kept at room temperature (ca. 20 °C) until a stationary concentration was reached (typically occurred in 3 days −1 week but was measured several times after this to ensure that a stable end concentration was reached). Then, a further 0.2 mL of stock solution was added and the amount of CrH measured periodically until a stable end point was reached. If no Cr–H was observable after equilibration, more was added until a Cr–H could be measured. The NMR measurements were carried out with a relaxation time of 30 s, 16 scans, and a spectral width of 30 ppm. Stacked NMR spectra of the titration of V_2_O_5_-comm are shown in Figure S13. The equilibrium concentration of Cr–H was obtained by integration of the hydride peak at −5.6 ppm vs the internal standard. The total adsorbed H· was obtained indirectly by subtraction of the amount of Cr–H remaining in solution at the stationary point from the total added Cr–H.

### Reversed PCET from V_2_O_5_ Morphologies to [CpCr­(CO)_3_]

Reversibility of PCET from Cr–H to V_2_O_5_ was tested by reacting the prereduced V_2_O_5_ samples (0.003–0.005 mmol, 5–8 mg) with 0.5 mL of a stock solution of [CpCr­(CO)_3_]_2_ (0.17 mmol, 75 mg) in CD_3_CN (3 mL) in a sealed NMR tube with mesitylene as an internal standard. The mixture was kept at room temperature (20 °C) for 12 h to ensure that enough time had passed for the reversed PCET to occur. Stacked NMR spectra of the reversibility experiments are shown in Figure S14.

### ReactIR Kinetics of PCET from Cr–H to V_2_O_5_

Measurement of the kinetics of PCET from Cr–H to various V_2_O_5_ samples was done using reactIR via a diamond-crystal dip-probe fitted into a Schlenk-flask filled with an appropriate amount of Cr–H solution (0.11 mmol, 22.2 mg) in 3 mL CH_3_CN. Additional Schlenk-flasks containing V_2_O_5_ (0.55 mmol, 100 mg) and 5 mL of CH_3_CN under Ar atmosphere were prepared and connected to a Schlenk-line. All flasks were held at 30 °C and stirred (200 rpm) to control the reaction conditions. The dip-probe was sealed in each flask with a PTFE-adapter fitted to a size 14 ground glass joint, inserted into the flask containing CH_3_CN, and sealed, to measure the solvent background. The dip-probe was then immersed into the Cr–H-solution and several IR spectra were measured until a stable spectrum was obtained. Then the dip-probe was cleaned with acetone, attached to the Schlenk-flask containing the V_2_O_5_ sample, and the Cr–H-solution was transferred into the Schlenk-flask. An IR spectrum of the reaction mixture was repeatedly measured (every 1–2 min, resolution of 4 cm^–1^ and 157 scans for 2–3 h). After the reactions the reduced sample was filtered, washed with 4 × 3 mL CH_3_CN, dried in vacuum and stored in Ar atmosphere for further characterization.

### Rietveld Refinement of Vanadium Oxide Samples

Rietveld refinement was performed with TOPAS Academic Version 7. The background was refined with 11 Chebychev polynomials. To account for the contribution of the machine optics in the peak shapes, crystalline silicon was refined prior to the experimental analysis. The peak shape was described by a simplified model for the axial divergence and the Thomson-Cox-Hastings and pseudo-Voigt functions. For all vanadium oxide samples, the corresponding parameters were fixed and further broadening was accounted for exclusively by the LVol function from TOPAS, which employs Gaussian and Lorentzian functions to extract the crystallite size. This allows a direct comparison of the latter between all samples. Besides the zero shift, all lattice parameters and the isotropic displacement parameters, all positional parameters of the atoms allowed by symmetry and the oxygen occupancies were refined. Preferred orientation was described by a set of spherical harmonics compatible with the V_2_O_5_ structure symmetry.

### V_2_O_5_-Catalyzed Oxidation of Methanol

Methanol oxidation catalyzed by V_2_O_5_ was carried out in a quartz flow reactor at 300 °C. Synthetic air was bubbled through liquid methanol at 20 mLn min^–1^ and allowed to flow over 300 mg of V_2_O_5_ catalyst at 300 °C with a weight hourly space velocity of 1.0 g_MeOH_ g_cat_^–1^ h^–1^. The selectivity and activity of the reaction were then sampled automatically every 20 min using an online GC (Figure S37) and averaged over multiple data points (in some cases up to 20 separate measurements over many hours). Deactivation was not observed for any catalyst over the observed reaction time (ca. 36 h on stream) as demonstrated by the plots of conversion vs temperature and conversion vs the inverse of the flow rate, both of which were measured randomly over the course of these 36 h (Figure S38). The temperature was then adjusted appropriately to achieve 50% MeOH conversion in order to compare the difference in selectivity between catalysts at the same conversion. The selectivity for formaldehyde at 50% conversion only showed very minor variations between catalysts. The reaction results for the various catalysts are given above in Table 2.

## Supplementary Material



## Notes

PXRD, SEM and STEM, NMR, IR, UV–vis, XANES, and ReactIR data, derivation of the rate law for PICET to V_2_O_5_, estimation of the BDFE­(OH) of V_2_O_5_ according to literature data. This material is available free of charge via at. The original raw data sets are all available for download free of charge from the Data Repository of the University of Stuttgart (DARUS) via the following DOI: 10.18419/DARUS-5136 (PDF)
