# Supplementary material for: The Effect of Nanoparticle Structure on the Thermodynamics and Kinetics of Proton-Coupled Electron Transfer Reactions to V2O5
Source: J Am Chem Soc. 2026 Jul 14;148(29):31302–15. doi: 10.1021/jacs.6c08557 (PMC13426264; doi:10.1021/jacs.6c08557)
Supplement: Supplementary file 1 [file ja6c08557_si_001.pdf]

The Effect of Nanoparticle Structure on the Thermodynamics and Kinetics of Proton-Coupled Electron Transfer Reactions to V_2O_5

Osman Bunjaku,† Mustafa Turan,† Hannes V. Beyertt, Wael Barakat, Michael Dyballa, Bertold Rasche,* Deven P. Estes*

Faculty of Chemistry, University of Stuttgart, Pfaffenwaldring 55, 70569 Stuttgart, Germany.
** deven.estes@itc.uni-stuttgart.de / bertold.rasche@itc.uni-stuttgart.de*

Supporting Information

Table of Contents

Characterization data of as synthesized V_2O_5	S2
Characterization data of reduced V_2O_5	S11
Monitoring of PCET Reactions by solution-phase NMR and IR	S14
Derivation of Rate Law for PICET from $CpCr(CO)_3H$ to V_2O_5	S39
Estimation of the H atom loading dependence of the BDFE(OH) of V_2O_5 as measured by Dickens and coworkers	S40
Catalytic Oxidation of Methanol to Formaldehyde	S41
References	S42

The raw data from this study are available for download free of charge via the Data Repository of the University of Stuttgart (DARUS) via the DOI: https://doi.org/10.18419/DARUS-5136

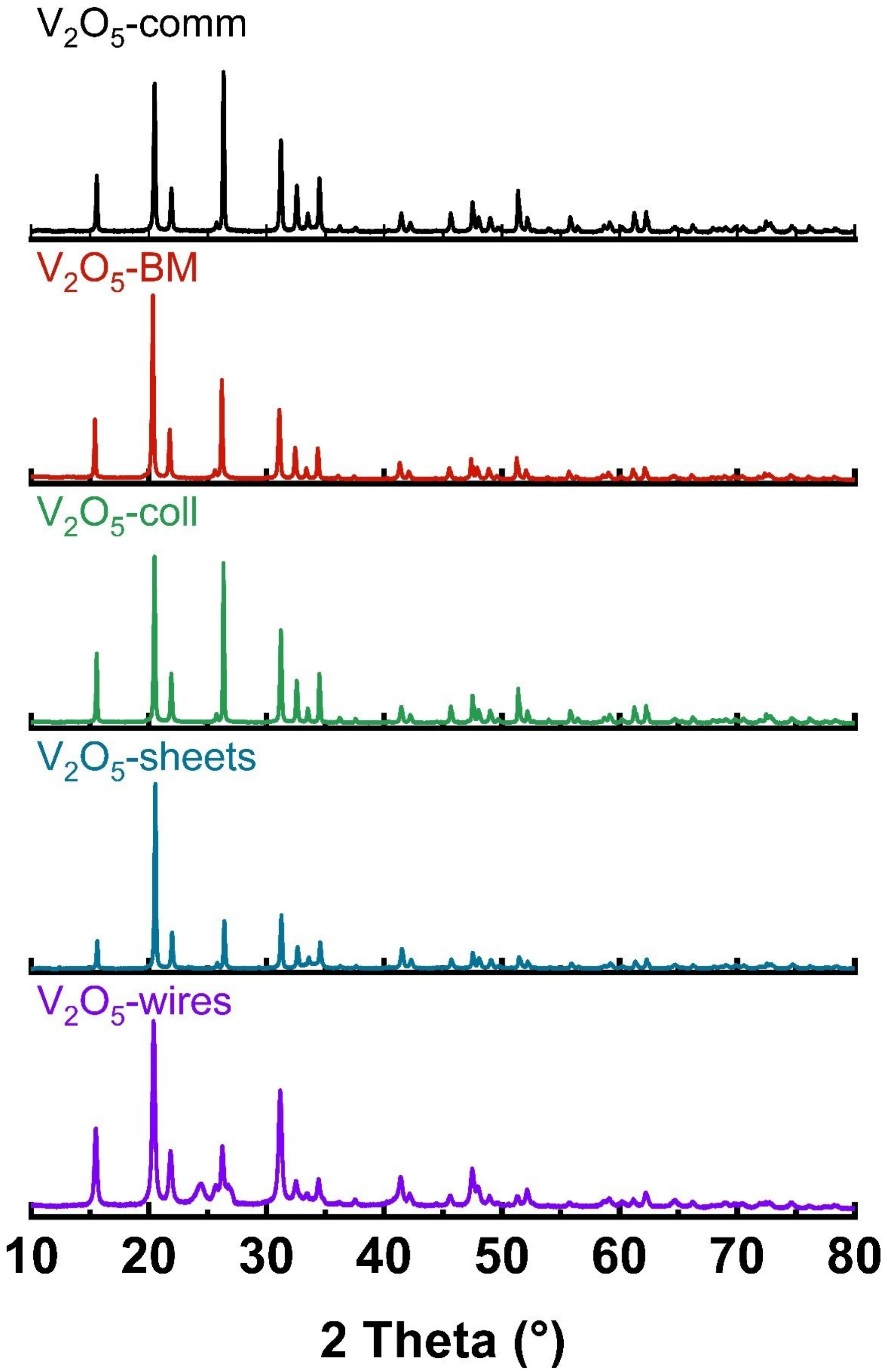

Figure S1. Powder XRD diffractograms of all morphologies with **V_2O_5-comm** (black), **V_2O_5-BM** (red), **V_2O_5-coll** (green), **V_2O_5-sheets** (blue) and **V_2O_5-wires** (violet).

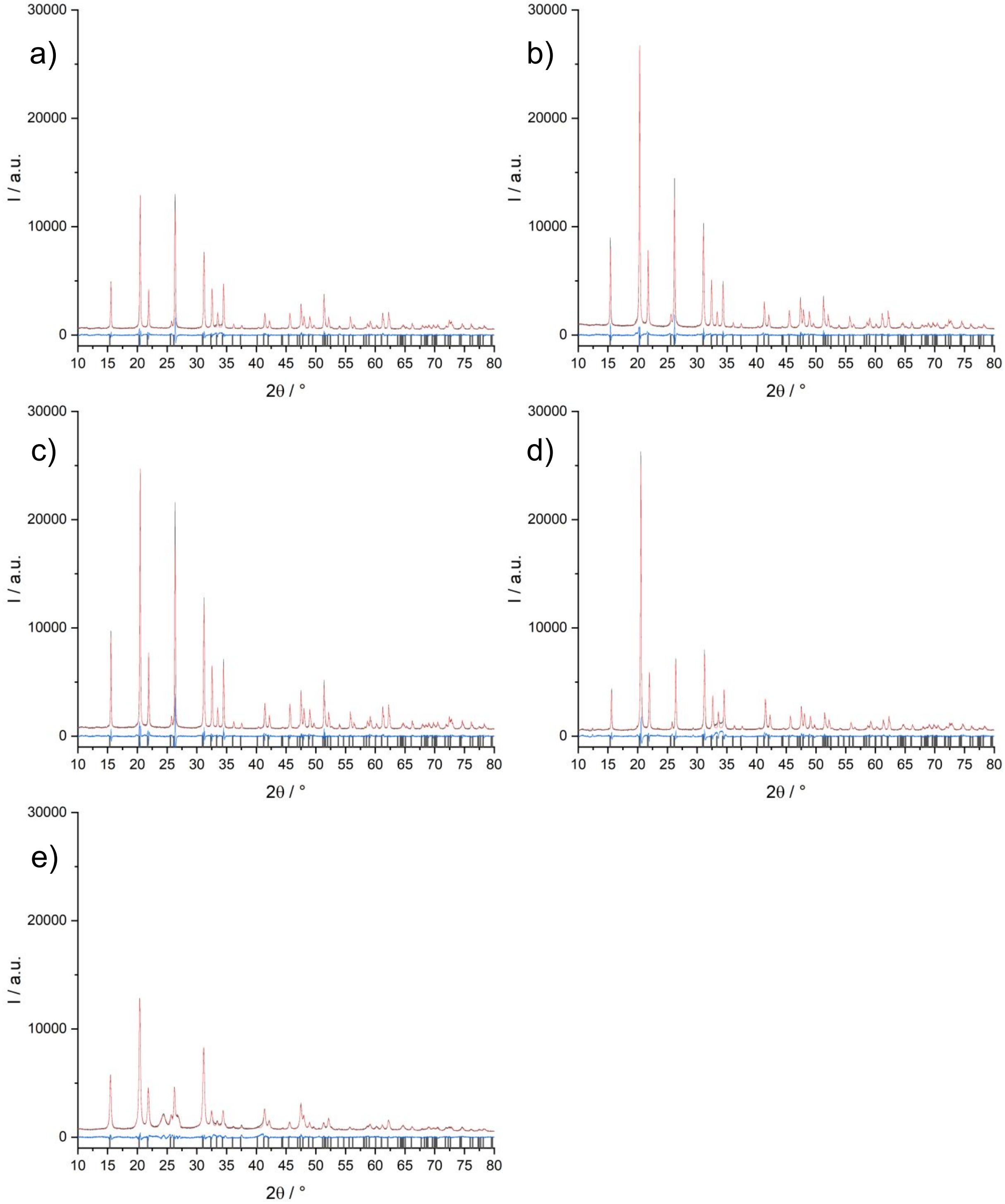

Figure S2. Rietveld refinements of the Powder XRD diffractograms of all morphologies (a) **V_2O_5-comm**, b) **V_2O_5-BM**, c) **V_2O_5-coll**, d) **V_2O_5-sheets** and e) **V_2O_5-wires**) showing the measured XRD (black), the fitted data from Rietveld (red) and the difference curve (blue). Tics indicate possible reflections for *Pmmn*. For the wires, two additional peaks were observed, possibly resulting from an amorphous side product.

Table S1. Parameters for all samples from the Rietveld refinement in Topas academic 7. Differences in the zero shift follow from the measurement setup, where the machine does not allow for an individual height adjustment. The particle size is calculated by the LVol function in TOPAS based on Gaussian and Lorentzian functions. In all cases the Simple_Axial parameter was fixed at 6.7 and the TCHZ parameters U, V, W, Z, Y and X were fixed at -0.015, 0.017, -0.0014, 0, 0.063, and 0.026 as refined for the silicon standard on the same machine and with the same setup.

V_2O_{5-x}		V_2O_5-BM		V_2O_5-coll		V_2O_5-comm		V_2O_5-sheets		V_2O_5-wires	
**zero shift**		0.0497	(3)	0.2055	(3)	0.2114	(4)	0.2695	(5)	0.1239	(8)
***a* / Å**		11.5117	(2)	11.5195	(1)	11.5218	(2)	11.5299	(2)	11.5120	(3)
***b* / Å**		3.5639	(1)	3.5658	(1)	3.5668	(1)	3.5646	(1)	3.5688	(1)
***c* / Å**		4.3725	(1)	4.3758	(1)	4.3762	(1)	4.3759	(1)	4.3734	(1)
**V1**	***x***	0.1010	(2)	0.1013	(2)	0.1014	(2)	0.1016	(2)	0.1004	(2)
Wyckoff 4f	***y***	0.2500	( )	0.2500	( )	0.2500	( )	0.2500	( )	0.2500	( )
	z	0.8925	(3)	0.8921	(3)	0.8930	(3)	0.8955	(3)	0.8986	(3)
**O1**	***x***	0.1089	(3)	0.1087	(3)	0.1100	(4)	0.1117	(5)	0.1196	(4)
Wyckoff 4f	***y***	0.2500	( )	0.2500	( )	0.2500	( )	0.2500	( )	0.2500	( )
	z	0.5400	(8)	0.5376	(9)	0.5355	(9)	0.5293	(9)	0.5275	(8)
**O2**	***x***	-0.0707	(3)	-0.0716	(4)	-0.0696	(4)	-0.0658	(6)	-0.0614	(5)
Wyckoff 4f	***y***	0.2500	( )	0.2500	( )	0.2500	( )	0.2500	( )	0.2500	( )
	z	0.0006	(14)	-0.0011	(16)	-0.0034	(17)	-0.0081	(22)	-0.0104	(21)
**O3**	***x***	0.2500	( )	0.2500	( )	0.2500	( )	0.2500	( )	0.2500	( )
Wyckoff 2a	***y***	0.2500	( )	0.2500	( )	0.2500	( )	0.2500	( )	0.2500	( )
	z	-0.0126	(9)	-0.0105	(11)	-0.0090	(11)	-0.0071	(11)	-0.0170	(11)
**occupancy**	**O1**	0.81	(1)	0.83	(1)	0.76	(1)	0.75	(1)	0.88	(2)
	**O2**	0.99	(1)	0.94	(1)	0.77	(1)	0.71	(2)	0.82	(2)
	**O3**	1.00	(1)	1.00	(2)	0.93	(2)	1.01	(2)	1.00	(2)
***x* (in V_2O_{5-x})**		0.41	(3)	0.47	(7)	1.01	(7)	1.07	(8)	0.59	(9)
**d / nm (from LVol)**		98.1	(5)	117.1	(23)	84.8	(13)	76.8	(13)	27.4	(3)
**beq**	**V**	1.8	(1)	1.3	(1)	0.5	(1)	1.1	(1)	1.8	(1)
	**O**	1.3	(1)	0.9	(2)	-1.0	(2)	-0.8	(2)	-0.1	(2)

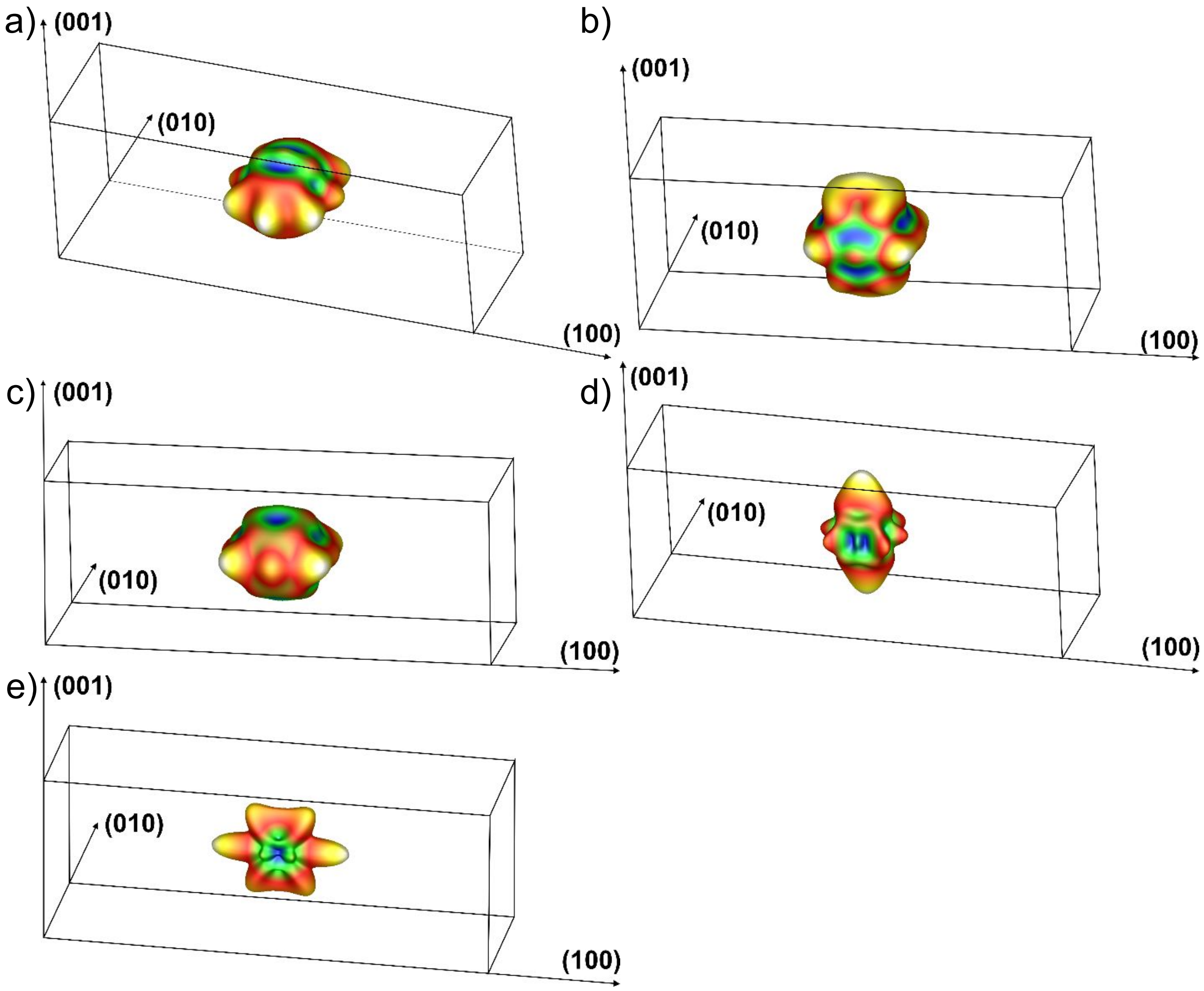

Figure S3. Graphical representation of the preferred orientation as calculated by Rietveld refinement from the XRD experiments for different morphologies: a) **V_2O_5-comm**, b) **V_2O_5-BM**, c) **V_2O_5-coll**, d) **V_2O_5-sheets** and e) **V_2O_5-wires**. Shown is the isosurface of the spherical harmonics representing the preferred orientation and the unit cell with (100), (010) and (001) referring to the a-, b- and c-axis.

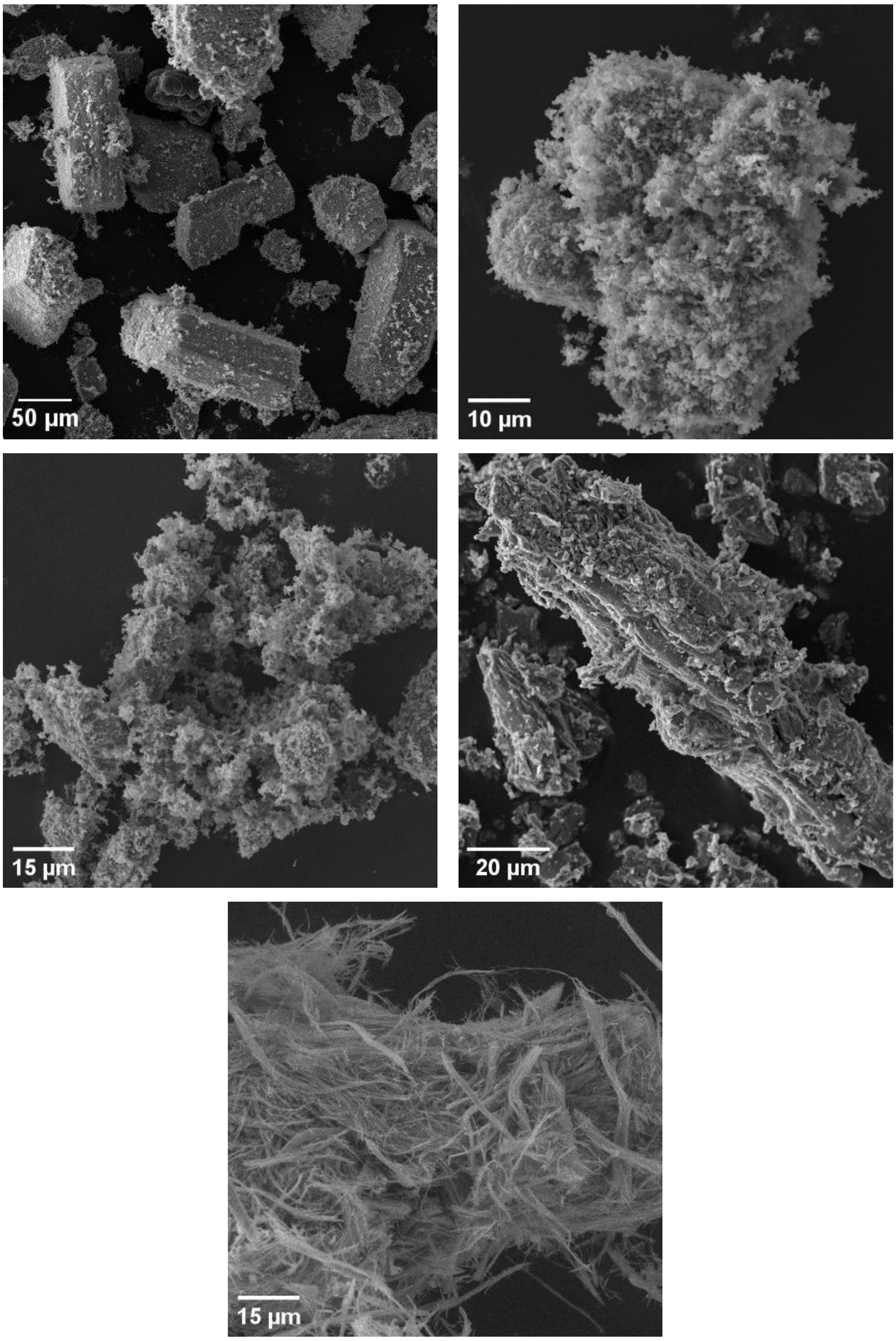

Figure S4. SEM images of a) **V_2O_5-comm**, b) **V_2O_5-BM**, c) **V_2O_5-coll**, d) **V_2O_5-sheets**, e) **V_2O_5-wires**.

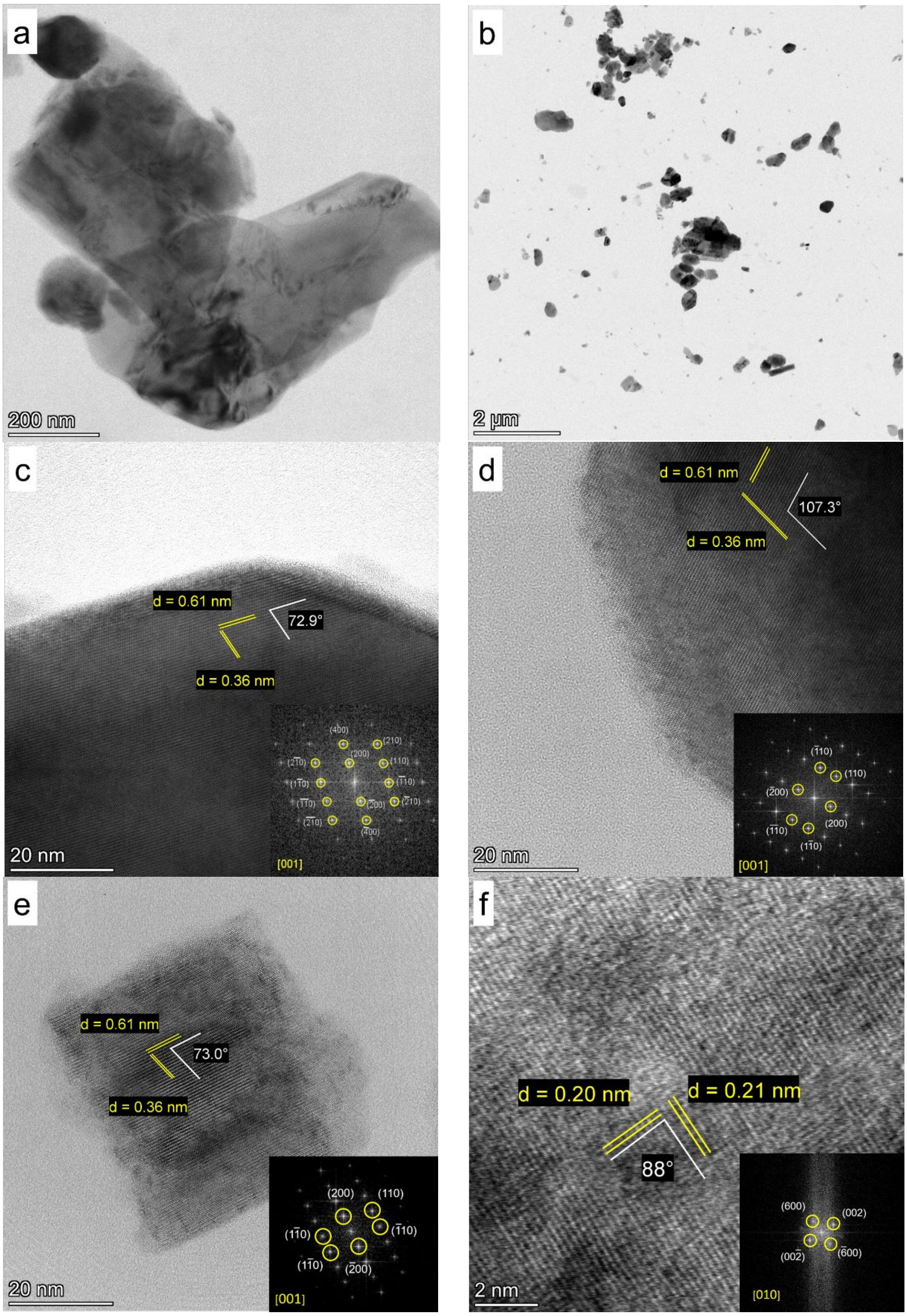

Figure S5. HRTEM images of **V_2O_5-coll** a-b) and Fast Fourier Transform pattern as insert with c-d) [001] crystal facet and f) [010] crystal facet.

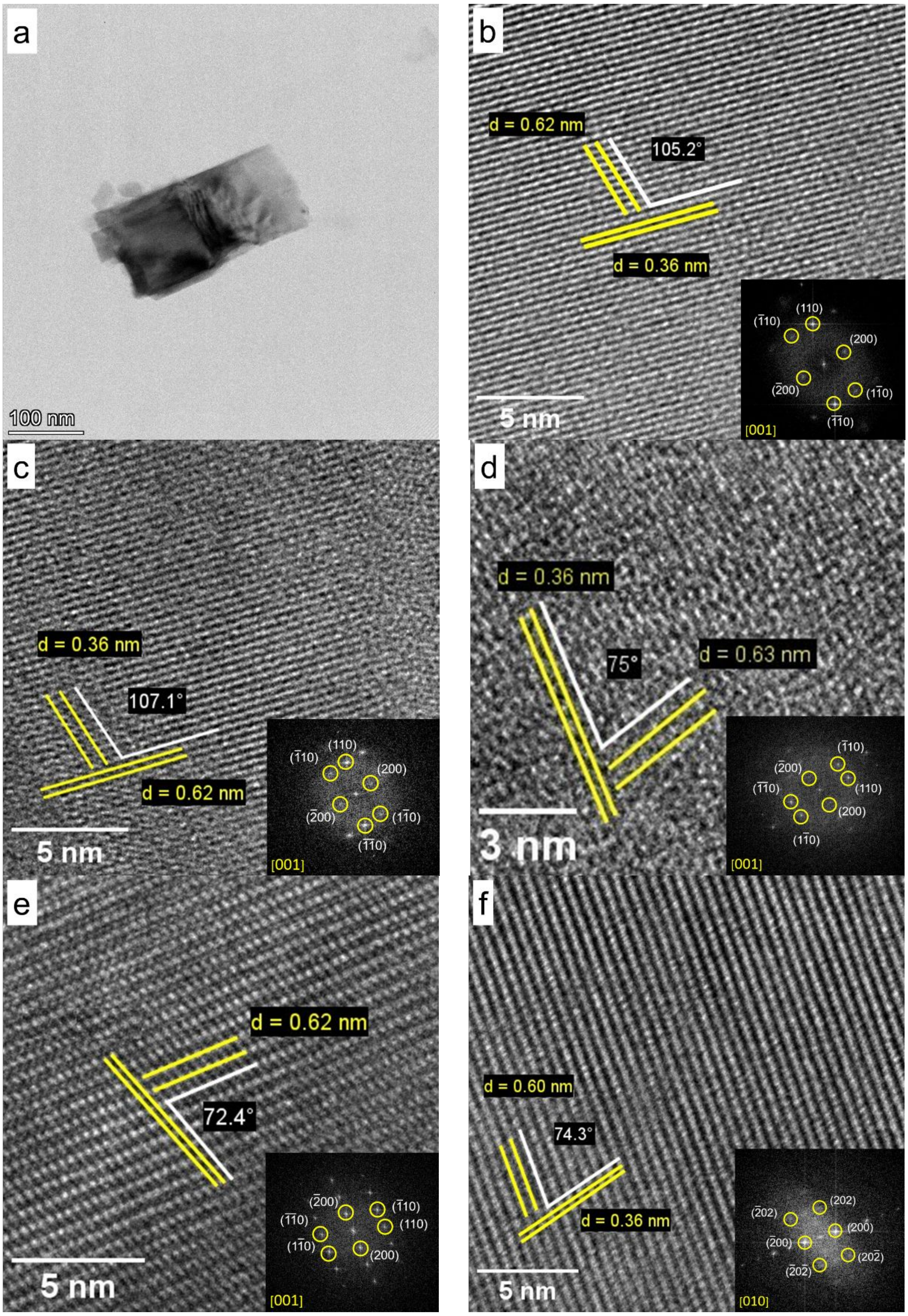

Figure S6. HRTEM images of **V_2O_5-sheets** a) and Fast Fourier Transform pattern as insert with b-e) [001] crystal facet and f) [010] crystal facet.

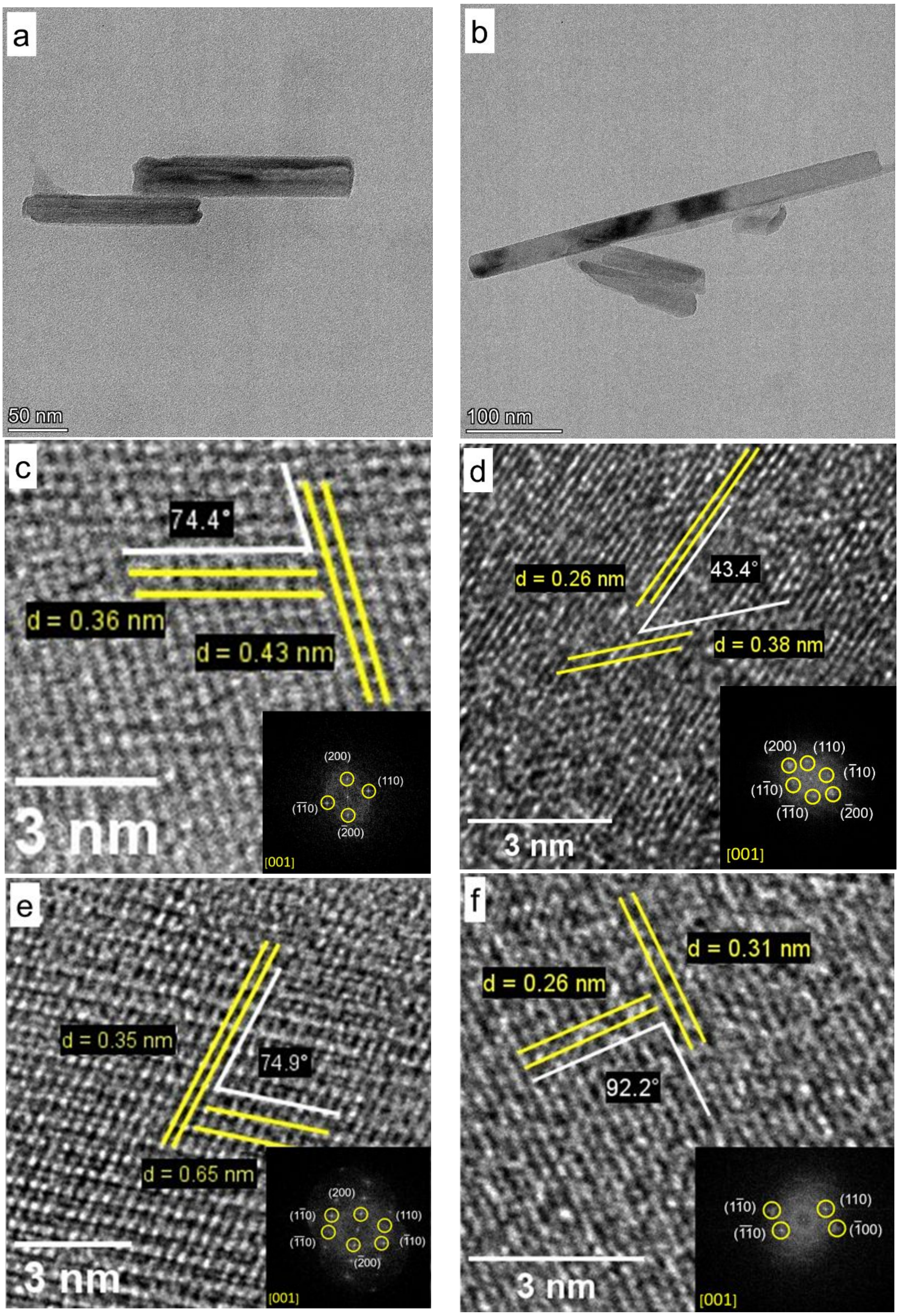

Figure S7. HRTEM images of **V_2O_5-wires** a-b) and Fast Fourier Transform pattern as insert with c-f) [001] crystal facet.

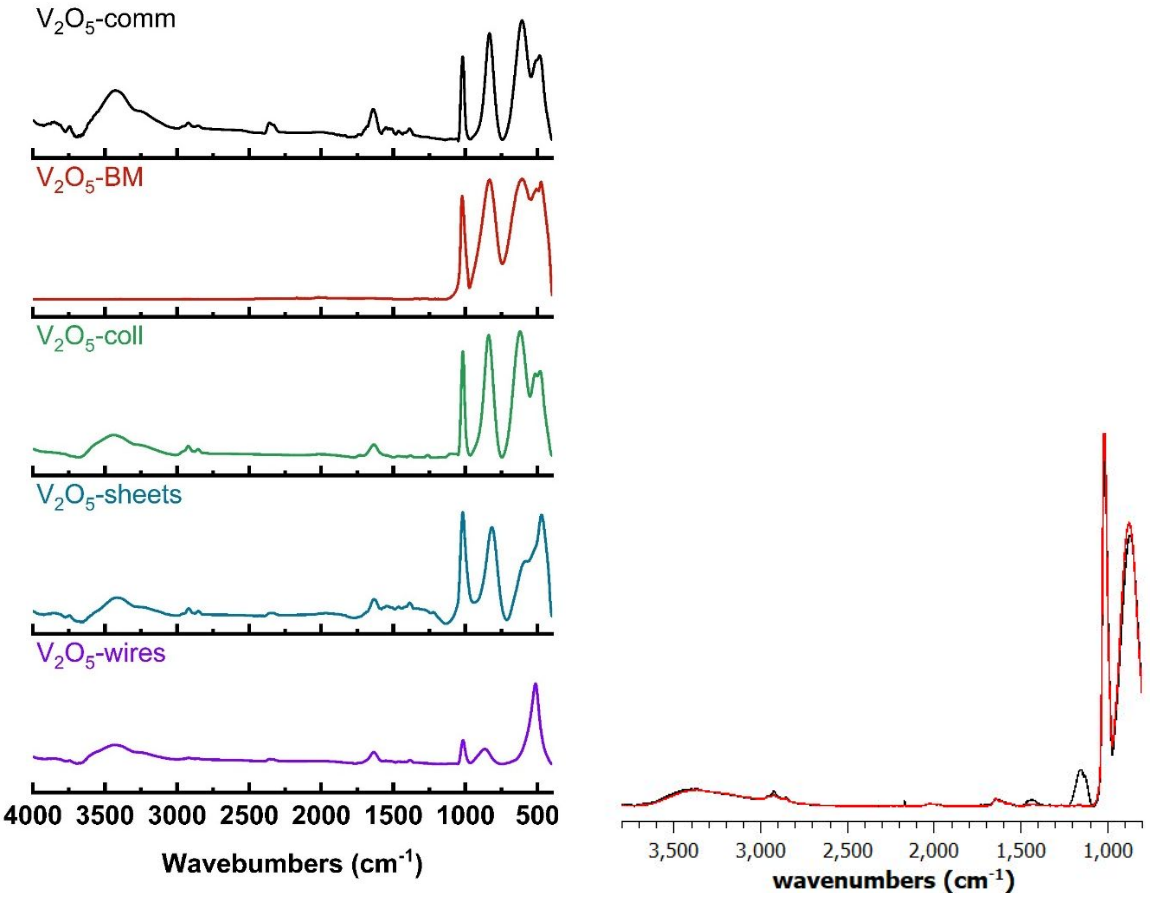

Figure S8. (left) IR spectra of all V_2O_5 materials with **V_2O_5-comm** (black), **V_2O_5-BM** (red), **V_2O_5-coll** (green), **V_2O_5-sheets** (blue) and **V_2O_5-wires** (violet). (right) **V_2O_5-wires** as synthesized and after stirring in acetonitrile to check for water incorporation.

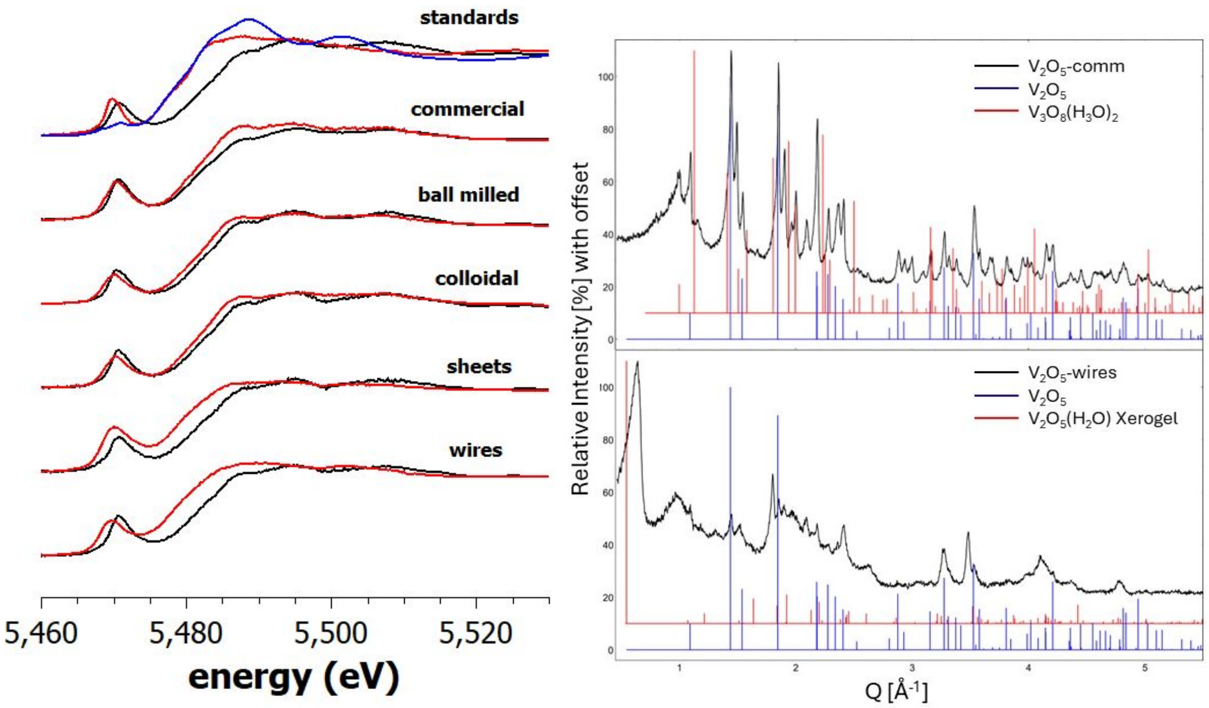

Figure S9. (Left) V K-edge XANES spectra of the as synthesized (black) V_2O_5 samples as well as the samples after reduction using $CpCr(CO)_3H$ under 13 bar H_2 (red) where the reduction is demonstrated by both lowering of the edge energy as well as the increased intensity of the edge peak at 5488 eV. (right) XRD patterns of V_2O_5-comm and V_2O_5-wires reduced by treatment with $CpCr(CO)_3H$ under 13 bar H_2 for 24 h.

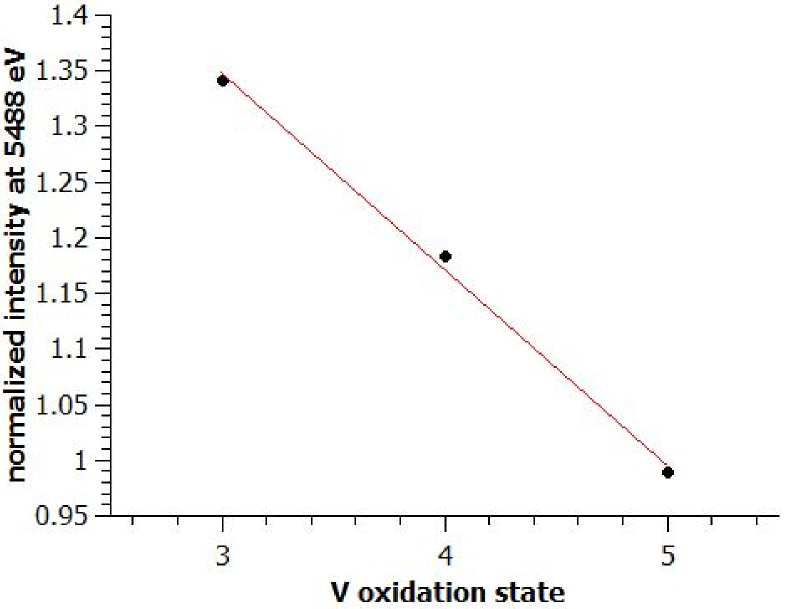

Figure S10. Plot of the normalized intensity of the V K-edge XANES spectrum at 5488 eV vs the V oxidation state. The standards and values are listed below in table S2.

Table S2. Analysis of the V K-edge XANES spectra of both as-synthesized and fully reduced V_2O_5 samples.

Sample	Edge energy	pre-edge energy	height at 5488 eV	average V oxidation state
	eV[a]	eV	*a.u.*	*a.u.*
V_2O_5	5481.6	5470.6	0.989	5
$(VO)SO_4 \cdot 4H_2O$	5477.9	5469.7	1.184	4
V_2O_3	5477.8	5471.0<br>5468.1	1.341	3
V_2O_5-comm	5481.2	5470.5	0.974	5.1
V_2O_5-BM	5481.7	5470.3	0.975	5.1
V_2O_5-coll	5481.5	5470.6	1.00	5.0
V_2O_5-sheets	5481.2	5470.7	0.996	5.0
V_2O_5-wires	5481.3	5470.5	0.984	5.1
V_2O_5-$comm_{red}$	5480.4	5469.8	1.10	4.4
V_2O_5-BM_{red}	5480.5	5469.3	1.10	4.4
V_2O_5-$coll_{red}$	5480.6	5469.8	1.08	4.5
V_2O_5-$sheets_{red}$	5480.4	5470.0	1.055	4.7
V_2O_5-$wires_{red}$	5478.2	5469.5	1.09	4.5

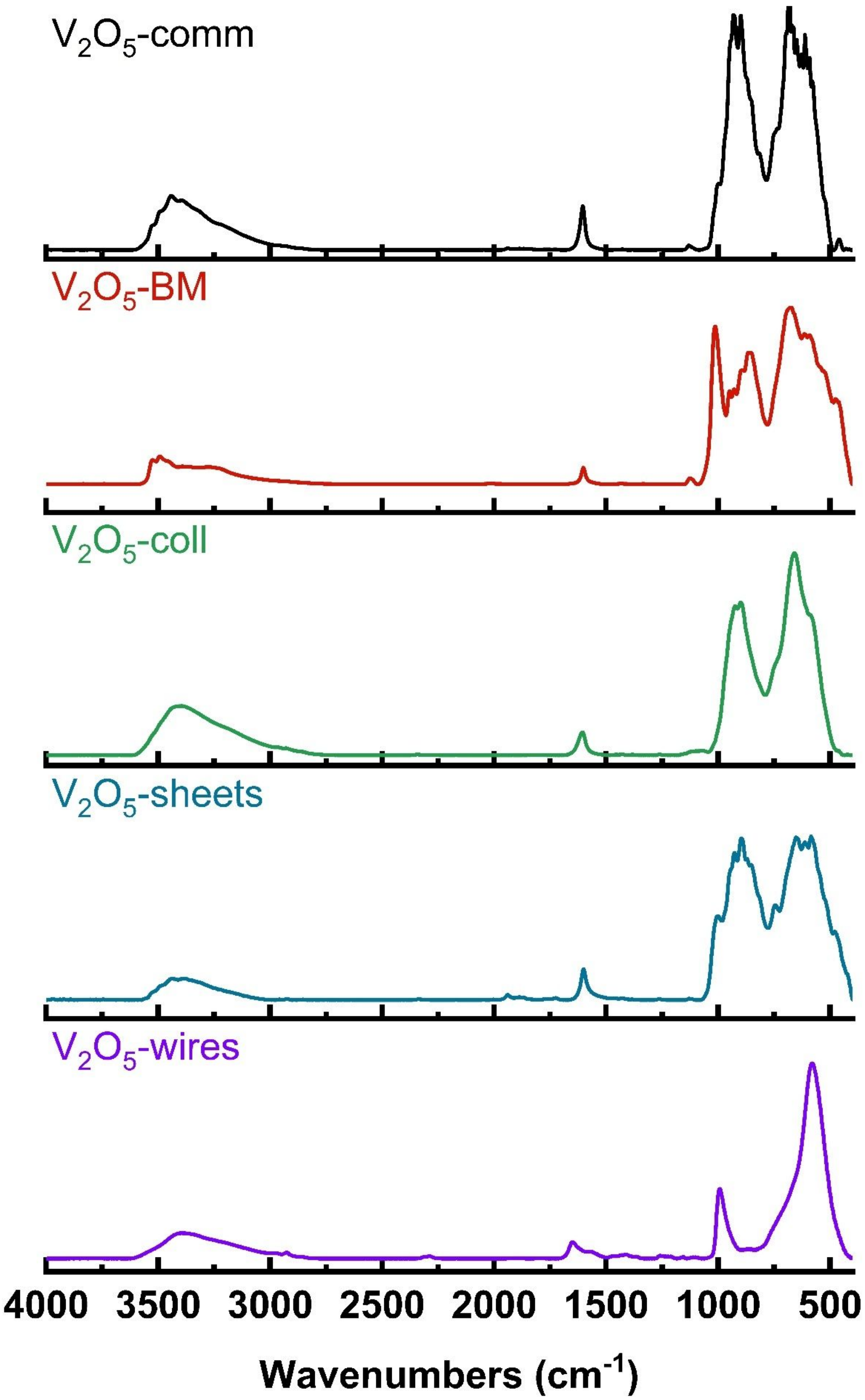

Figure S11. IR spectra of all reduced V_2O_5 materials with **V_2O_5-comm$_{red}$** (black), **V_2O_5-BM$_{red}$** (red), **V_2O_5-coll$_{red}$** (green), **V_2O_5-sheets$_{red}$** (blue) and **V_2O_5-wires$_{red}$** (violet).

Monitoring of PICET reactions via IR and NMR

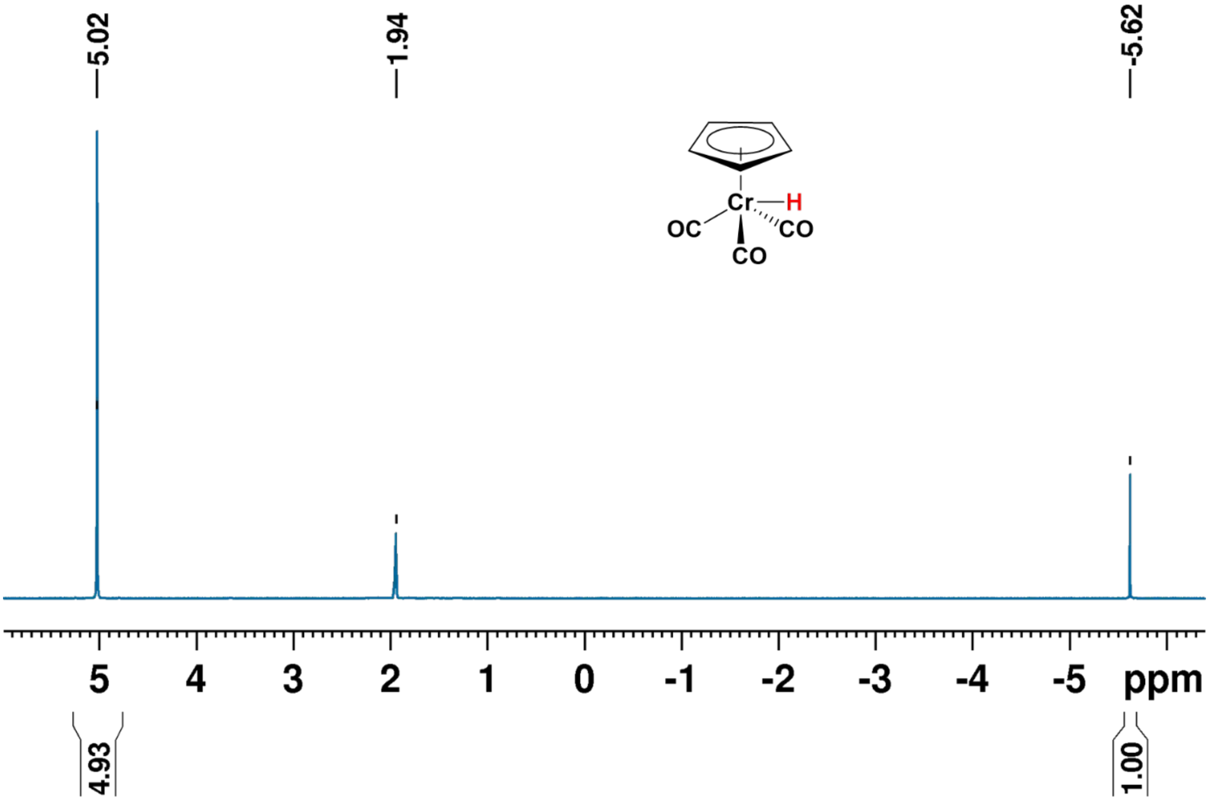

Figure S12. ^{1}H NMR spectra of $CpCr(CO)_3H$ in CD_3CN (1.94 ppm) with characteristic hydride peak at -5.62 ppm and Cp peak at 5.02 ppm

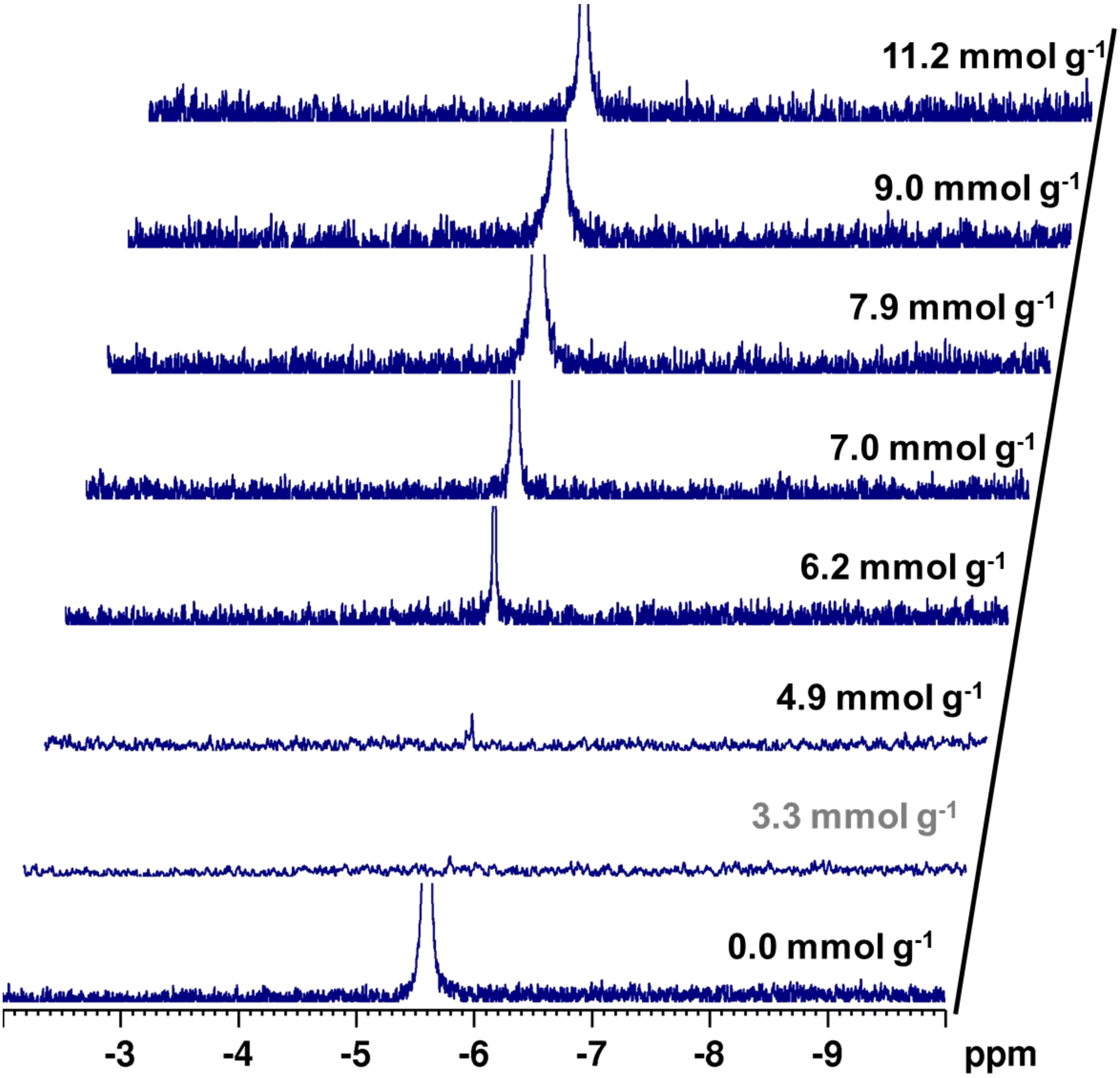

Figure S13. ^{1}H NMR stacked spectra of titration of **V_2O_5-comm** with $CpCr(CO)_3H$ (Cr-H peak at -5.6 ppm) and increasing hydrogen adsorption $n(Cr\text{-}H)_{added} - n(Cr\text{-}H)_{sol}$ from bottom to top. Grayed out numbers indicate cases in which CrH could not be measured accurately by NMR.

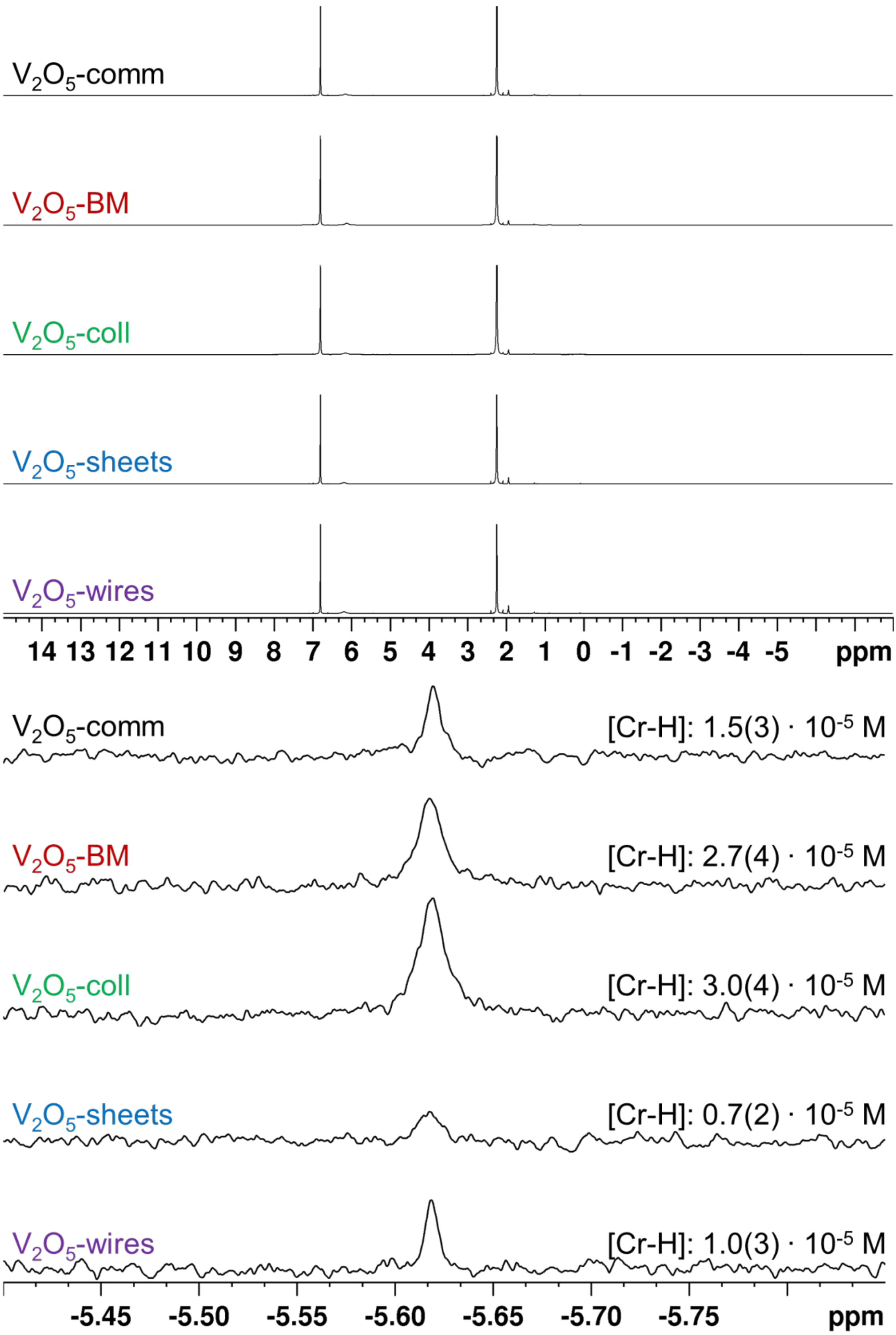

Figure S14. ^{1}H NMR stacked spectra of [CpCr(CO)$_3$] solution with fully reduced **V_2O_5** morphologies in CD_3CN vs. mesitylene (2.24 and 6.80 ppm) (top). The peak at -5.62 ppm in the bottom stacked spectra is the hydride resonance of CpCr(CO)$_3$H formed by reverse proton-electron transfer from reduced V_2O_5 to CpCr(CO)$_3$ radicals.

Table S3. Equilibrium concentrations [Cr-H]$_{eq.}$ of all fully reduced **V_2O_5** morphologies of the reversed PICET to $[CpCr(CO)_3]_2$ in CD_3CN and masses of V_2O_5 samples. The error of [CrH]$_{eq.}$ was determined by propagation of uncertainty (signal to noise of ΔI = 0.0001, mass uncertainty Δm = 0.5 mg and volume uncertainty ΔV = 0.02 mL). The H atom loading of the materials (n_H) was determined by V K-edge XANES spectroscopy.

Sample	[Cr-H]$_{eq.}$ (10^{-4} M)	m (V_2O_5) (10^{-3} g)	m (mesitylene) (10^{-3} g)	n_H (mmol H g^{-1})
**V_2O_5-comm**	1.5(3)	4.9(5)	11.2(5)	6.5(1.1)
**V_2O_5-BM**	2.7(4)	5.2(5)	10.1(5)	6.5(1.1)
**V_2O_5-coll**	3.0(4)	5.4(5)	9.1(5)	5.3(1.1)
**V_2O_5-sheets**	0.7(2)	8.5(5)	10.9(5)	3.7(1.1)
**V_2O_5-wires**	1.0(3)	7.1(5)	10.1(5)	5.9(1.1)

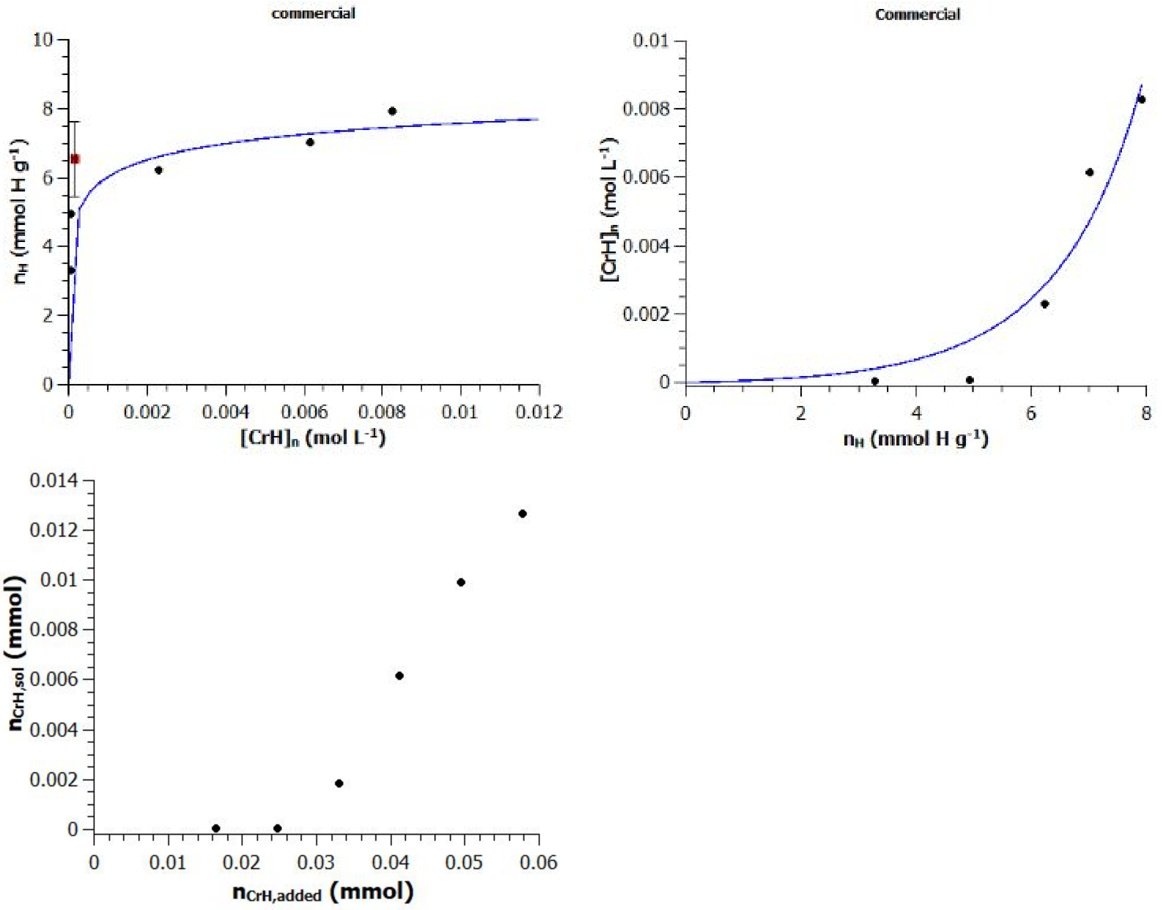

Figure S15. V_2O_5-comm titrated with $CpCr(CO)_3H$ fitted to both the Langmuir-Freundlich (left) and Frumkin-Fowler-Guggenheim (right) isotherms. The red point is the test of reversibility of the PCET reaction by treatment of the pre-reduced sample with $[CpCr(CO)_3]_2$ (table S3). (bottom) Amount of CrH remaining in solution after reaction with **V_2O_5-comm** vs. total amount of CrH added.

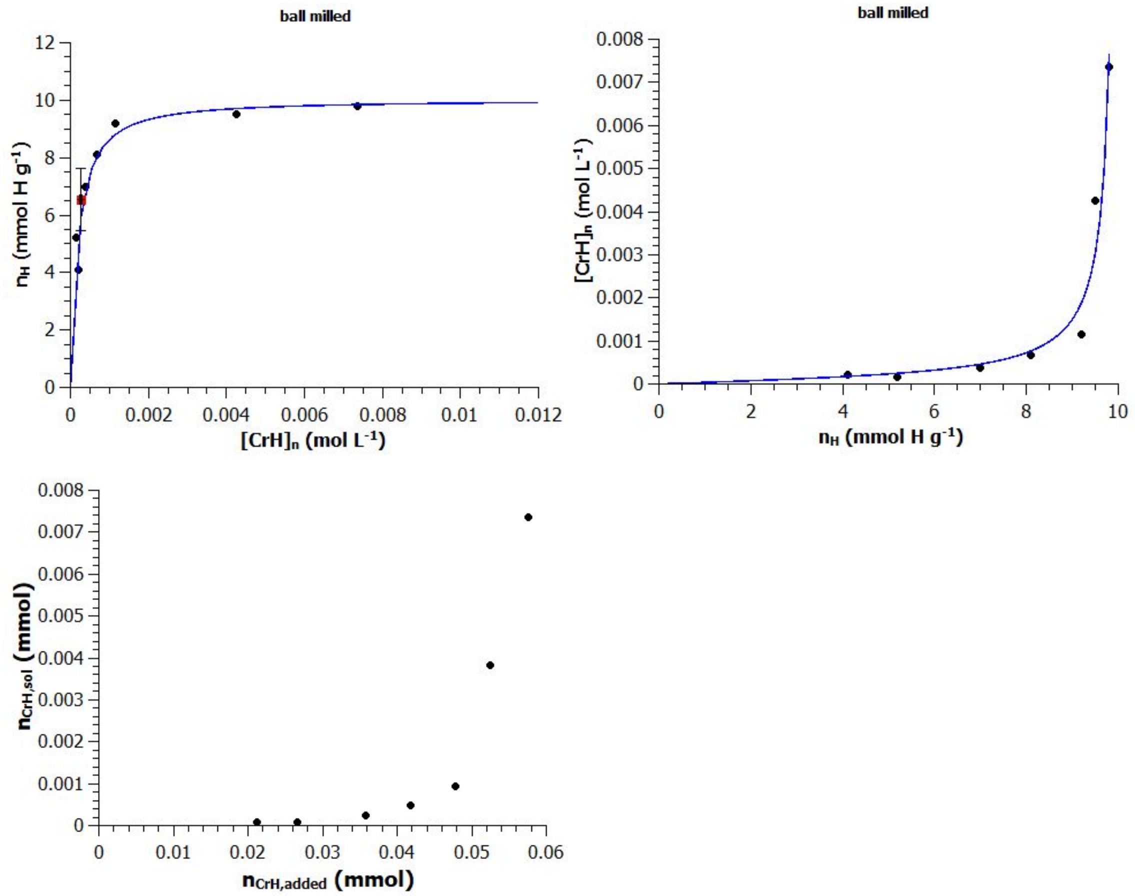

Figure S16. V_2O_5-BM titrated with $CpCr(CO)_3H$ fitted to both the Langmuir-Freundlich (left) and Frumkin-Fowler-Guggenheim (right) isotherms. The red point is the test of reversibility of the PCET reaction by treatment of the pre-reduced sample with $[CpCr(CO)_3]_2$ (table S3). (bottom) Amount of CrH remaining in solution after reaction with **V_2O_5-BM** vs. total amount of CrH added.

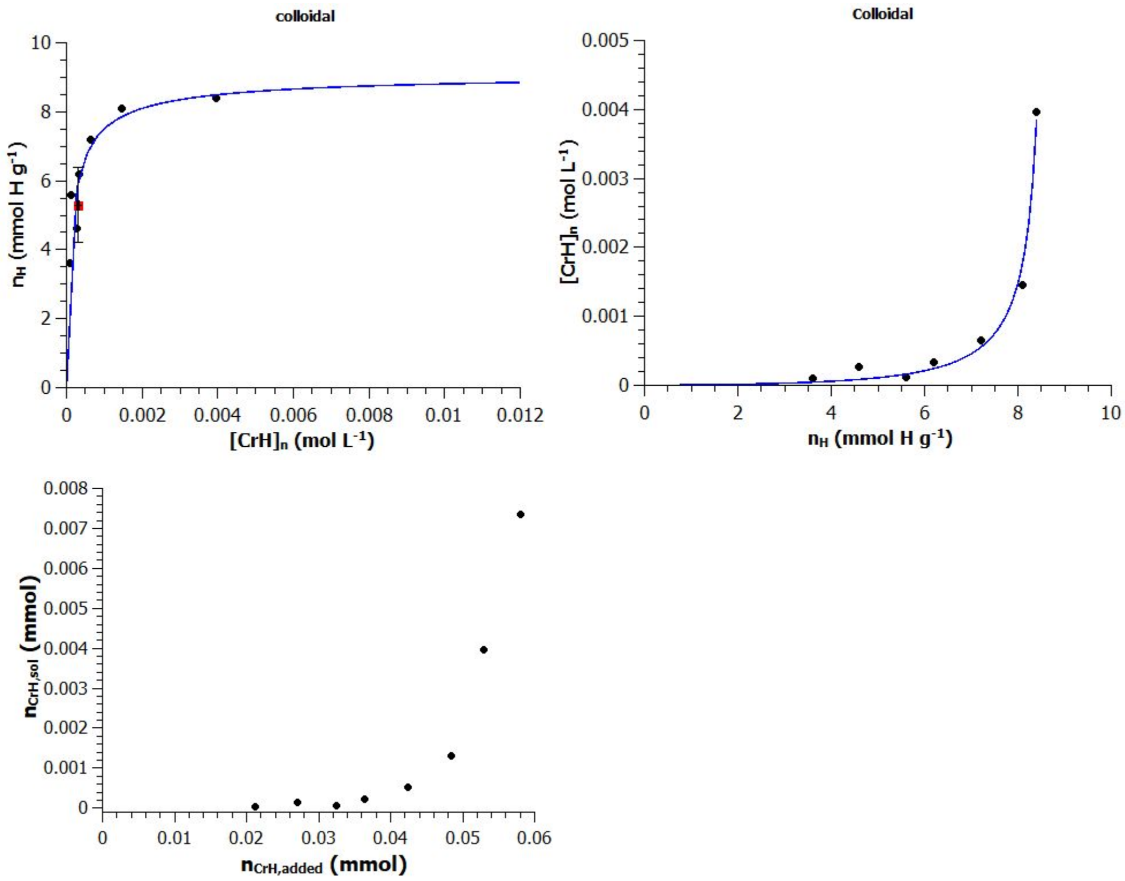

Figure S17. V_2O_5-coll titrated with $CpCr(CO)_3H$ fitted to both the Langmuir-Freundlich (left) and Frumkin-Fowler-Guggenheim (right) isotherms. The red point is the test of reversibility of the PCET reaction by treatment of the pre-reduced sample with $[CpCr(CO)_3]_2$ (table S3). (bottom) Amount of CrH remaining in solution after reaction with **V_2O_5-coll** vs. total amount of CrH added.

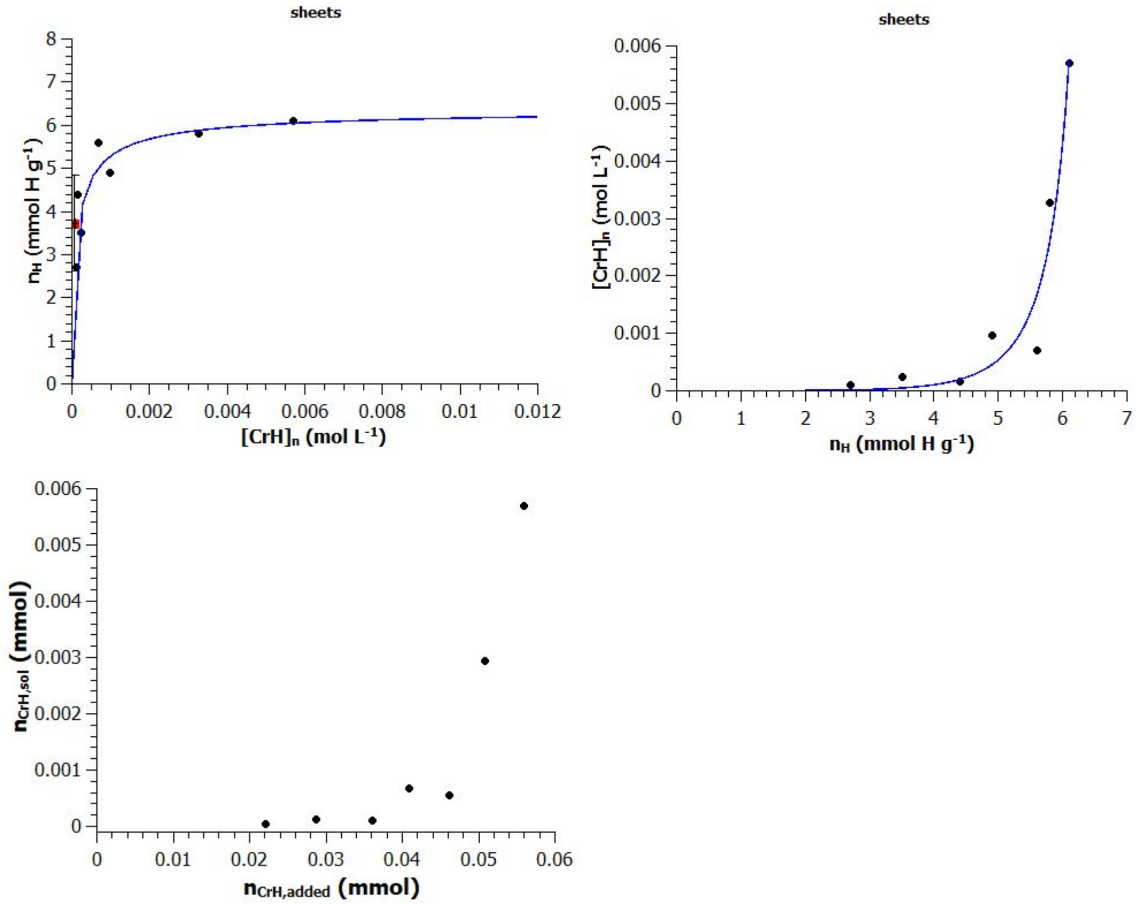

Figure S18. V_2O_5-sheets titrated with $CpCr(CO)_3H$ fitted to both the Langmuir-Freundlich (left) and Frumkin-Fowler-Guggenheim (right) isotherms. The red point is the test of reversibility of the PCET reaction by treatment of the pre-reduced sample with $[CpCr(CO)_3]_2$ (table S3). (bottom) Amount of CrH remaining in solution after reaction with **V_2O_5-sheets** vs. total amount of CrH added.

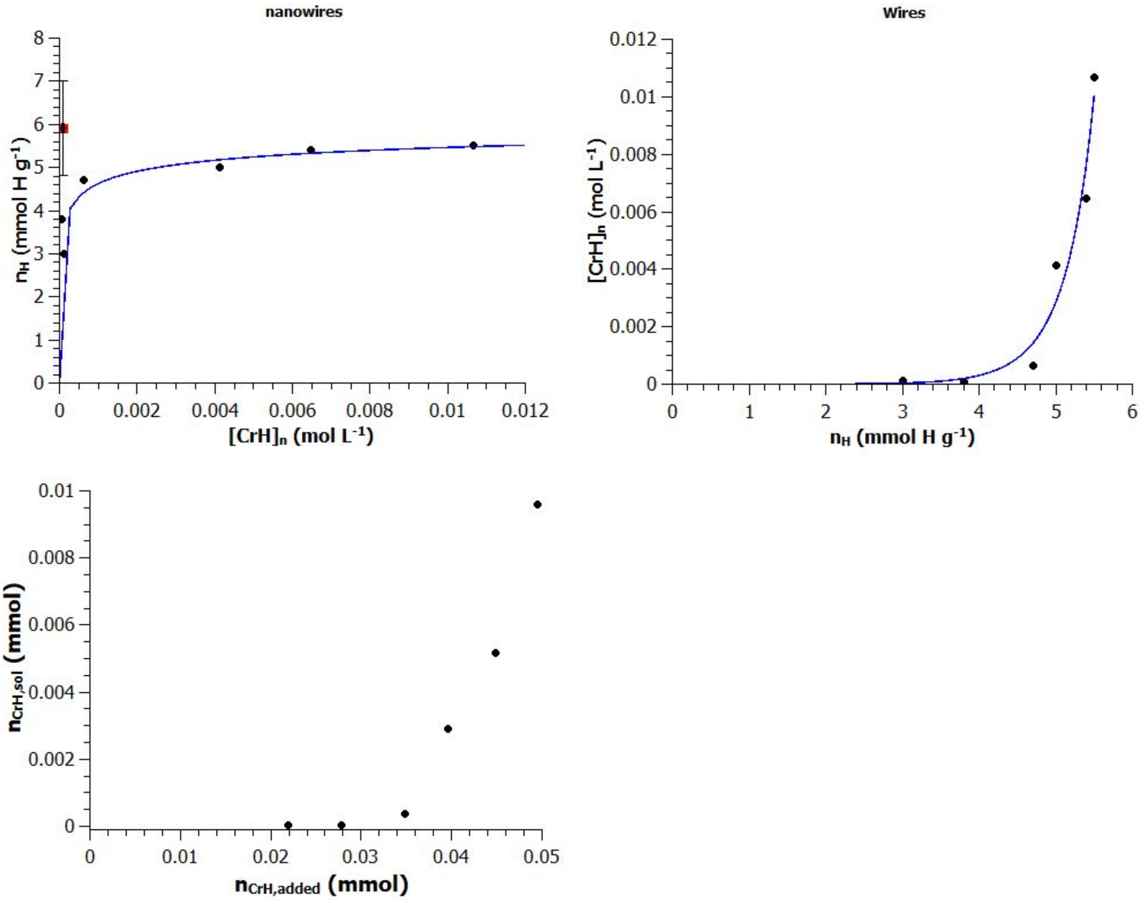

Figure S19. $\mathbf{V_2O_5}$**-wires** titrated with $CpCr(CO)_3H$ fitted to both the Langmuir-Freundlich (left) and Frumkin-Fowler-Guggenheim (right) isotherms. The red point is the test of reversibility of the PCET reaction by treatment of the pre-reduced sample with $[CpCr(CO)_3]_2$ (table S3). (bottom) Amount of CrH remaining in solution after reaction with $\mathbf{V_2O_5}$**-wires** vs. total amount of CrH added.

Table S4. Fitting parameters optimized from fitting the titrations of the V_2O_5 samples with $CpCr(CO)_3H$ to both a modified Langmuir-Freundlich and a modified Frumkin-Fowler-Guggenheim (FFG) isotherm.

sample	Langmuir-Freundlich			Frumkin-Fowler-Guggenheim		
	q_H	*a*	*b*	q_H	K_{eq}	*g*
V_2O_5-comm	11(2)	0.016(7)	0.26(3)	11(2)	6(3)	-3.3(2)
V_2O_5-BM	10.0(5)	40(9)	1.1(3)	10(2)	8(3)	0.84(7)
V_2O_5-coll	9.1(8)	2.3(7)	0.76(3)	8.7(2)	75(8)	-1.3(2)
V_2O_5-sheets	6.4(5)	1.9(6)	0.70(2)	6.6(9)	1600(800)	-6.0(8)
V_2O_5-wires	6.7(6)	0.05(2)	0.30(6)	6.4(1.1)	5600(2000)	-9.6(1)

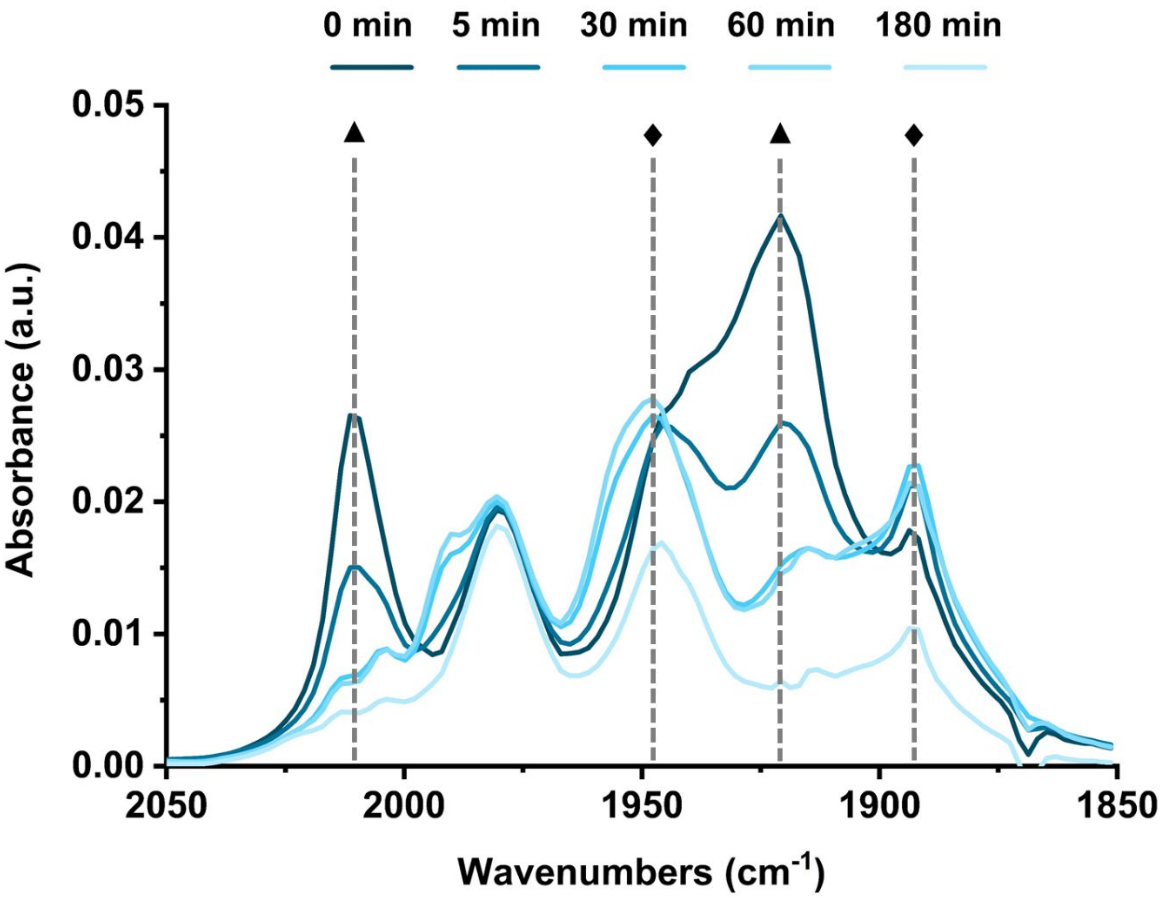

Figure S20. IR spectra of CO bands of CrH ▲ (2011 cm^{-1} and 1920 cm^{-1}) and dimeric Cr-complex ♦ (1948 cm^{-1} and 1894 cm^{-1}) at different times. The band at 1980 cm^{-1} is a solvent depended artefact.

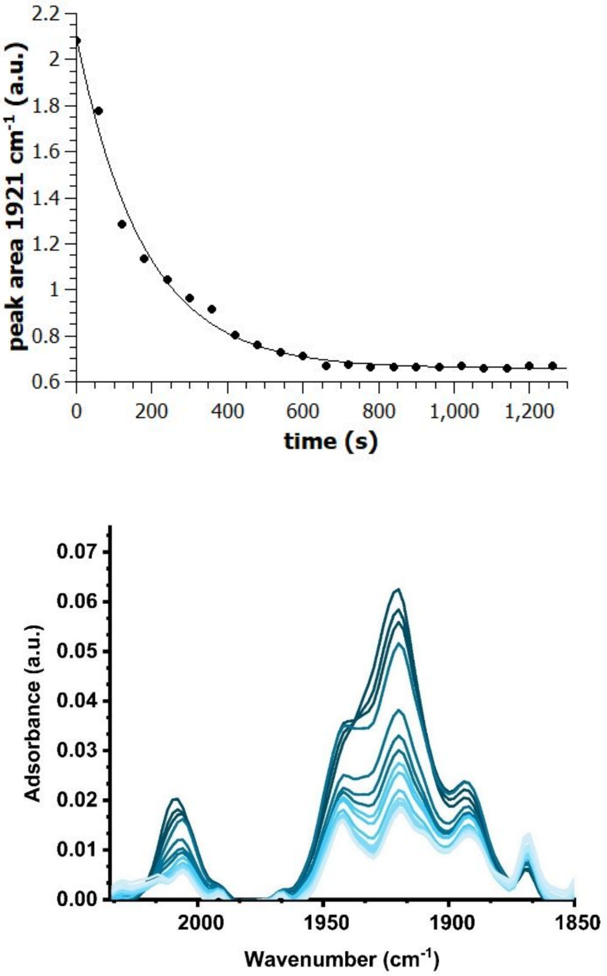

Figure S21. Absorption profiles of run 1 $CpCr(CO)_3H$ vs time (top) with selected IR spectra from 1850 cm^{-1} to 2040 cm^{-1} (bottom) during PICET to **V_2O_5-comm**.

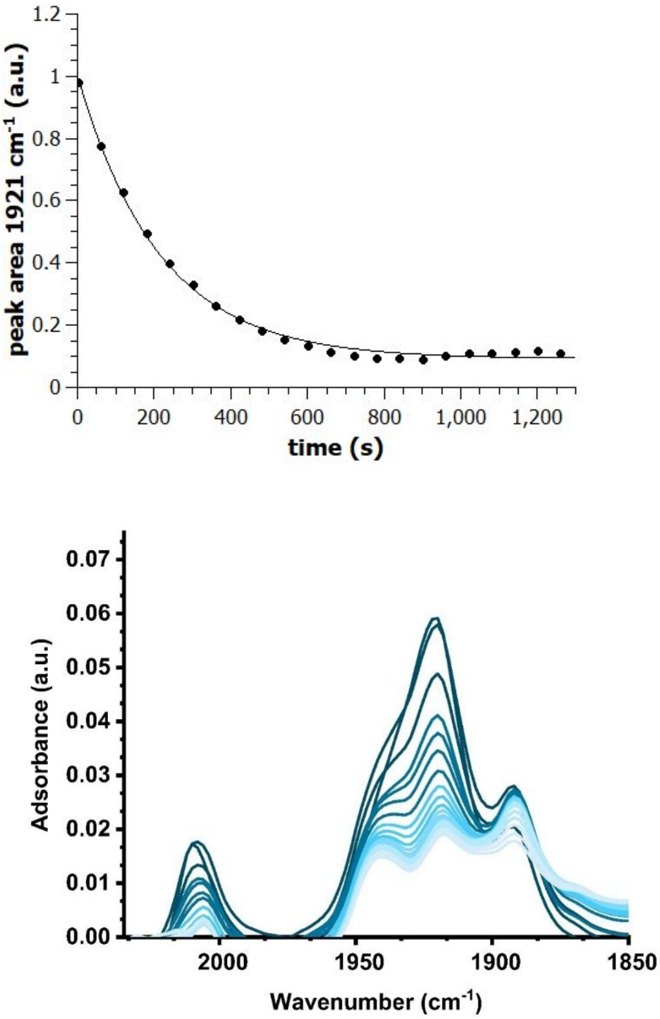

Figure S22. Absorption profiles of run 2 $CpCr(CO)_3H$ vs time (top) with selected IR spectra from 1850 cm^{-1} to 2040 cm^{-1} (bottom) during PICET to **V_2O_5-comm**.

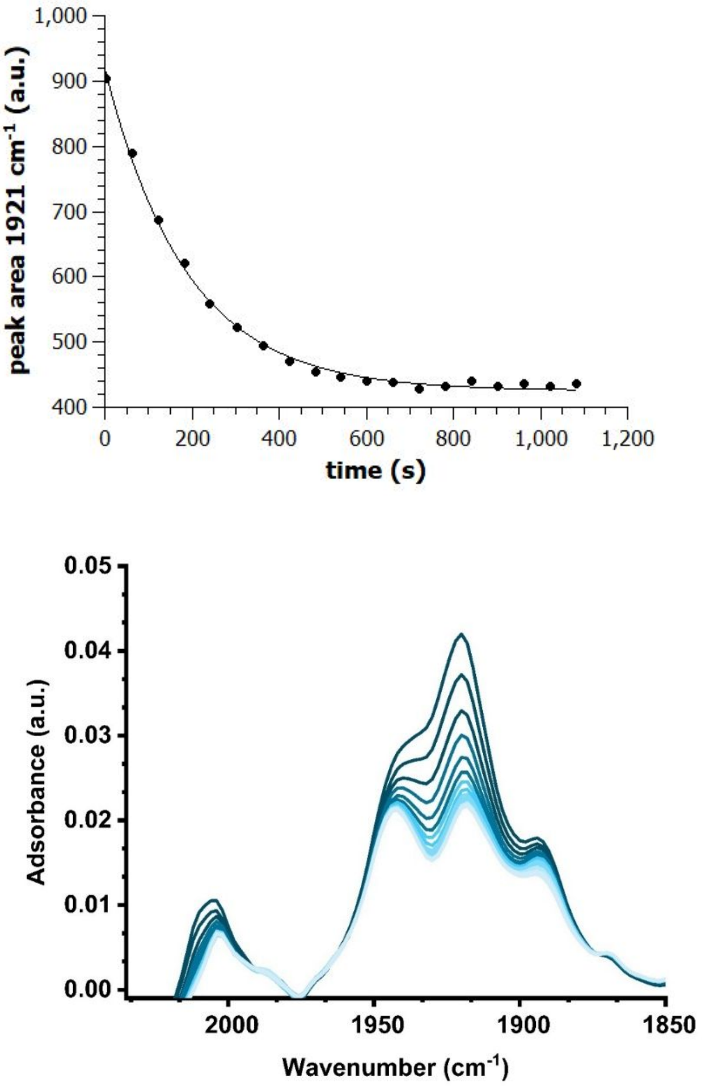

Figure S23. Absorption profiles of run 3 $CpCr(CO)_3H$ vs time (top) with selected IR spectra from 1850 cm^{-1} to 2040 cm^{-1} (bottom) during PICET to **V_2O_5-comm**.

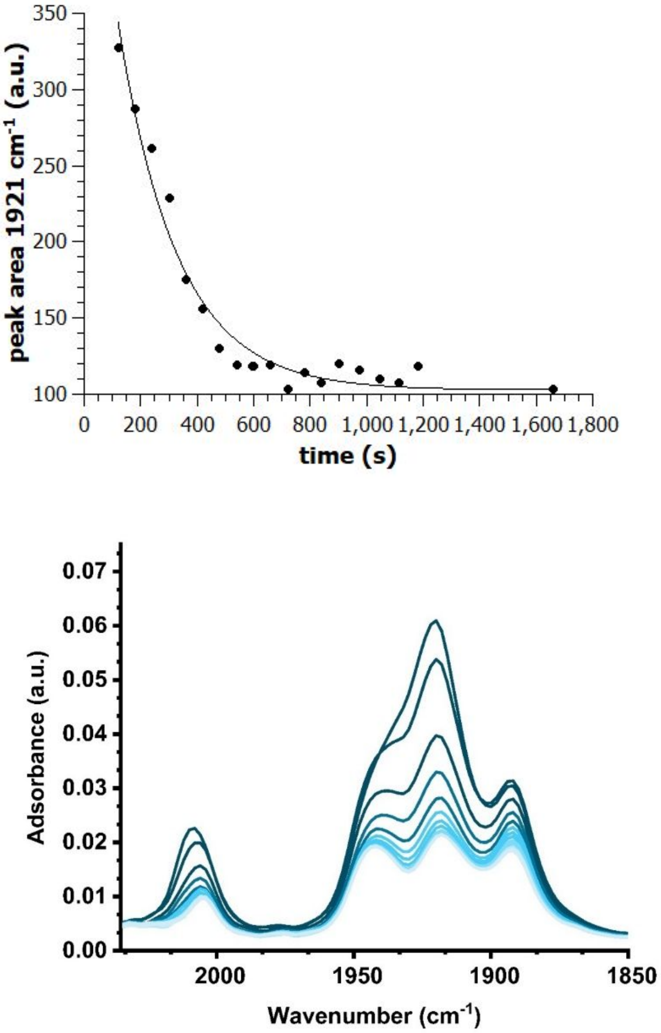

Figure S24. Absorption profiles of run 1 $CpCr(CO)_3H$ vs time (top) with selected IR spectra from 1850 cm^{-1} to 2040 cm^{-1} (bottom) during PICET to **V_2O_5-coll**.

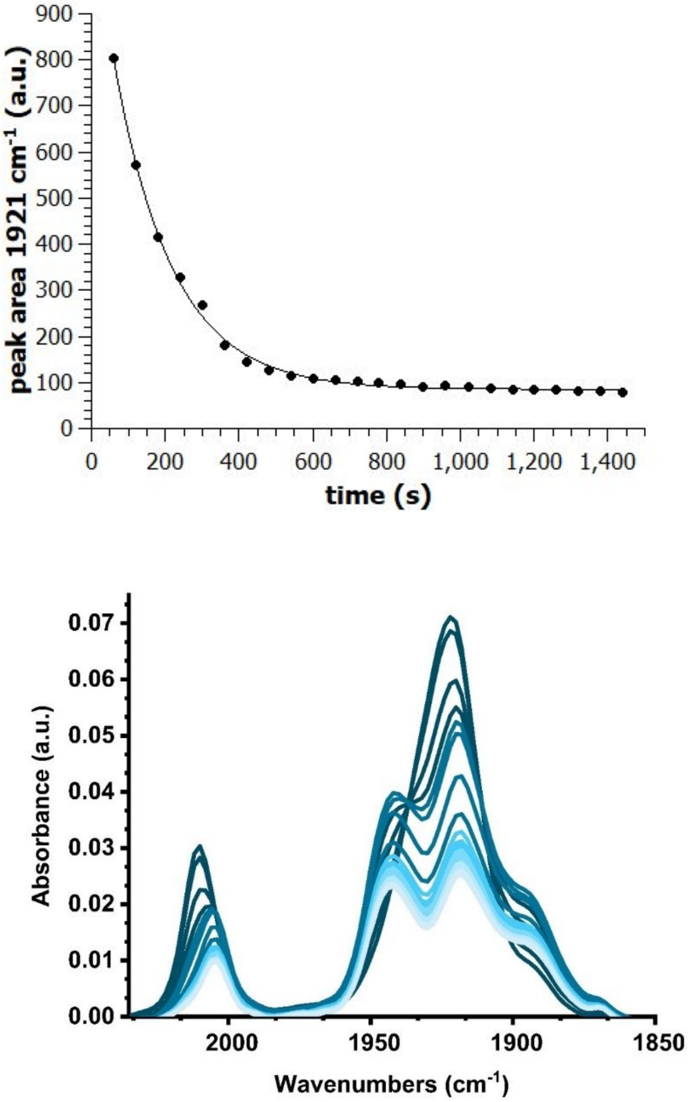

Figure S25. Absorption profiles of run 2 CpCr(CO)$_3$H vs time (top) with selected IR spectra from 1850 cm^{-1} to 2040 cm^{-1} (bottom) during PICET to **V_2O_5-coll**.

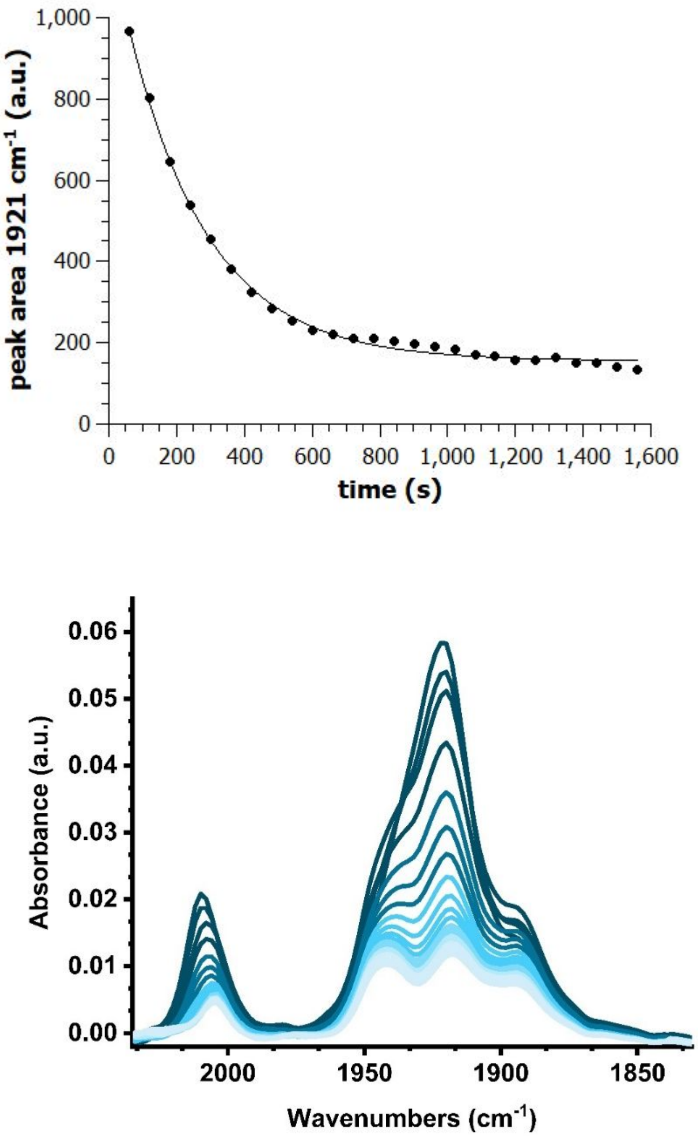

Figure S26. Absorption profiles of run 3 $CpCr(CO)_3H$ vs time (top) with selected IR spectra from 1850 cm^{-1} to 2040 cm^{-1} (bottom) during PICET to **V_2O_5-coll**.

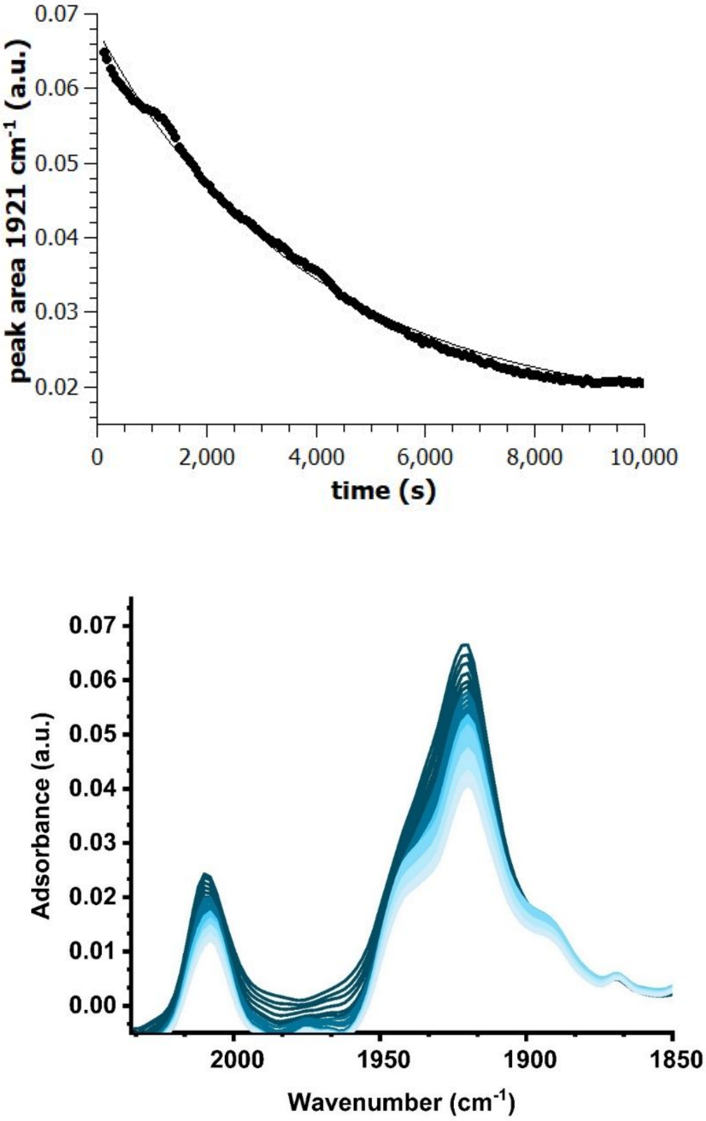

Figure S27. Absorptions profiles of run 1 $CpCr(CO)_3H$ vs time (top) with selected IR spectra from 1850 cm^{-1} to 2040 cm^{-1} (bottom) during PICET to **V_2O_5-sheets**.

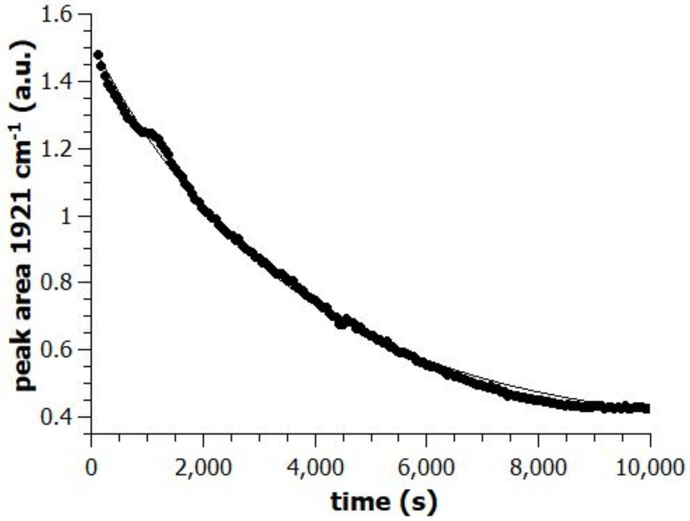

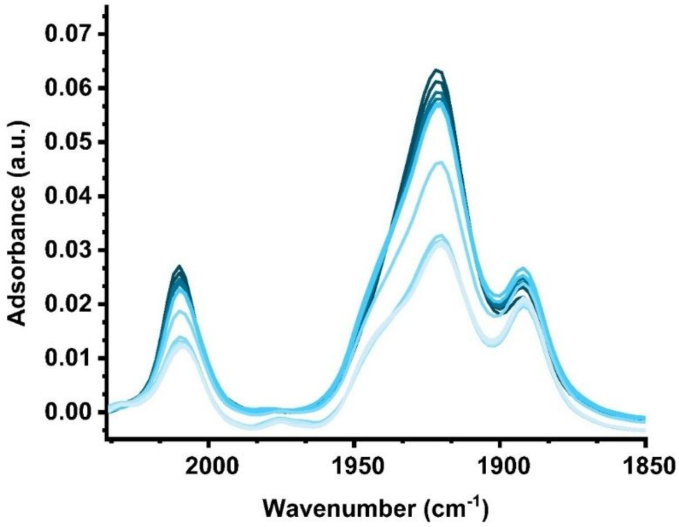

Figure S28. Absorption profiles of run 2 $CpCr(CO)_3H$ vs time (top) with selected IR spectra from 1850 cm^{-1} to 2040 cm^{-1} (bottom) during PICET to **V_2O_5-sheets**.

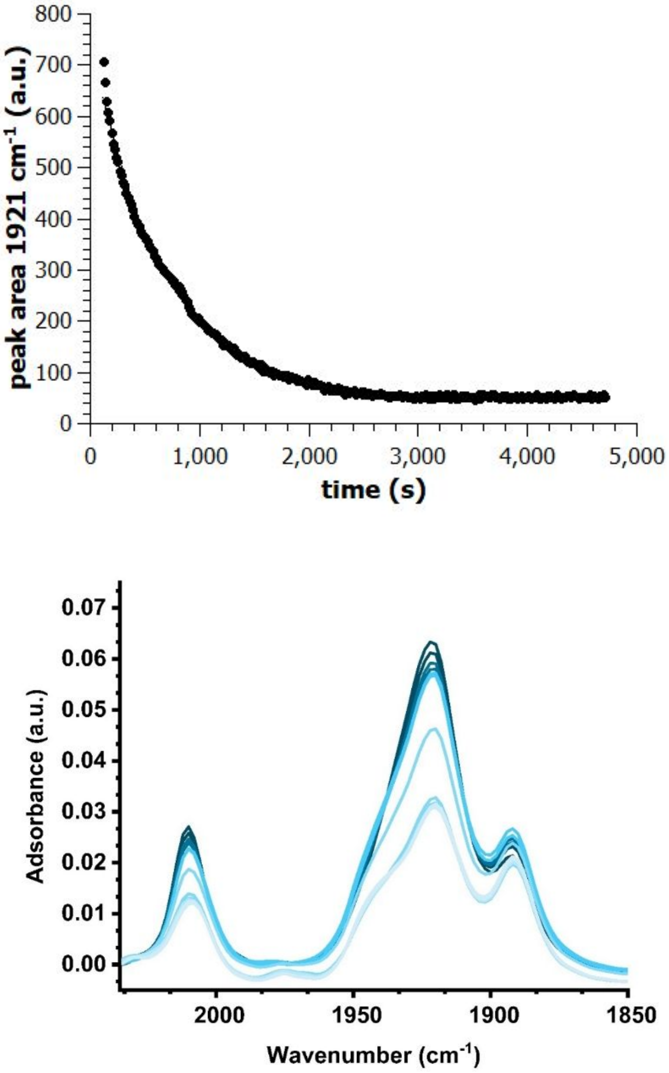

Figure S29. Absorption profiles of run 3 $CpCr(CO)_3H$ vs time (top) with selected IR spectra from 1850 cm^{-1} to 2040 cm^{-1} (bottom) during PICET to **V_2O_5-sheets**. (this sample was done with 182 mg of V_2O_5 instead of 100 mg to see the effect on the rate.

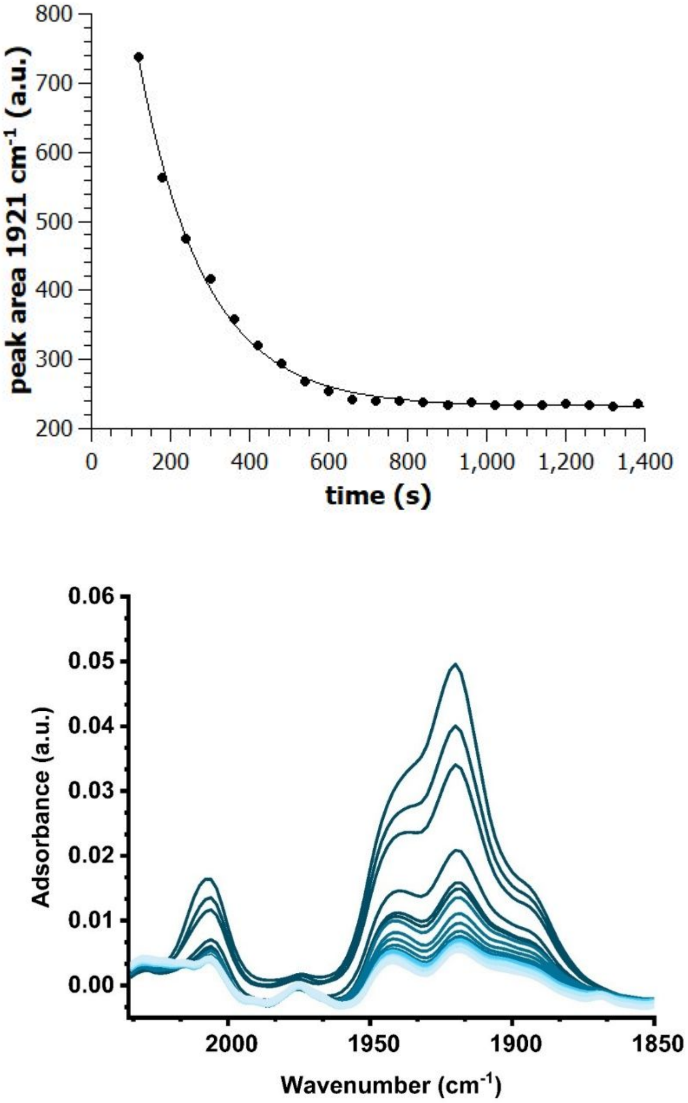

Figure S30. Absorption profiles of run 1 $CpCr(CO)_3H$ vs time (top) with selected IR spectra from 1850 cm^{-1} to 2040 cm^{-1} (bottom) during PICET to **V_2O_5-wires**.

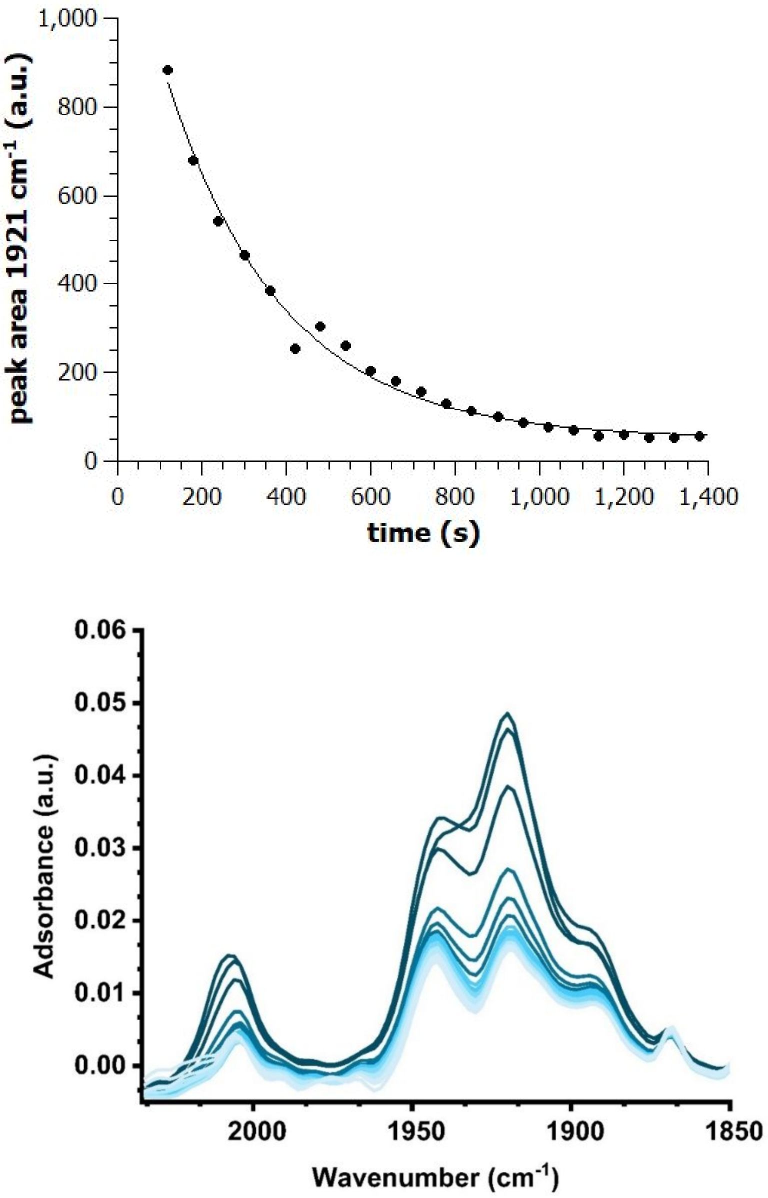

Figure S31. Absorption profiles of run 2 $CpCr(CO)_3H$ vs time (top) with selected IR spectra from 1850 cm^{-1} to 2040 cm^{-1} (bottom) during PICET to **V_2O_5-wires**.

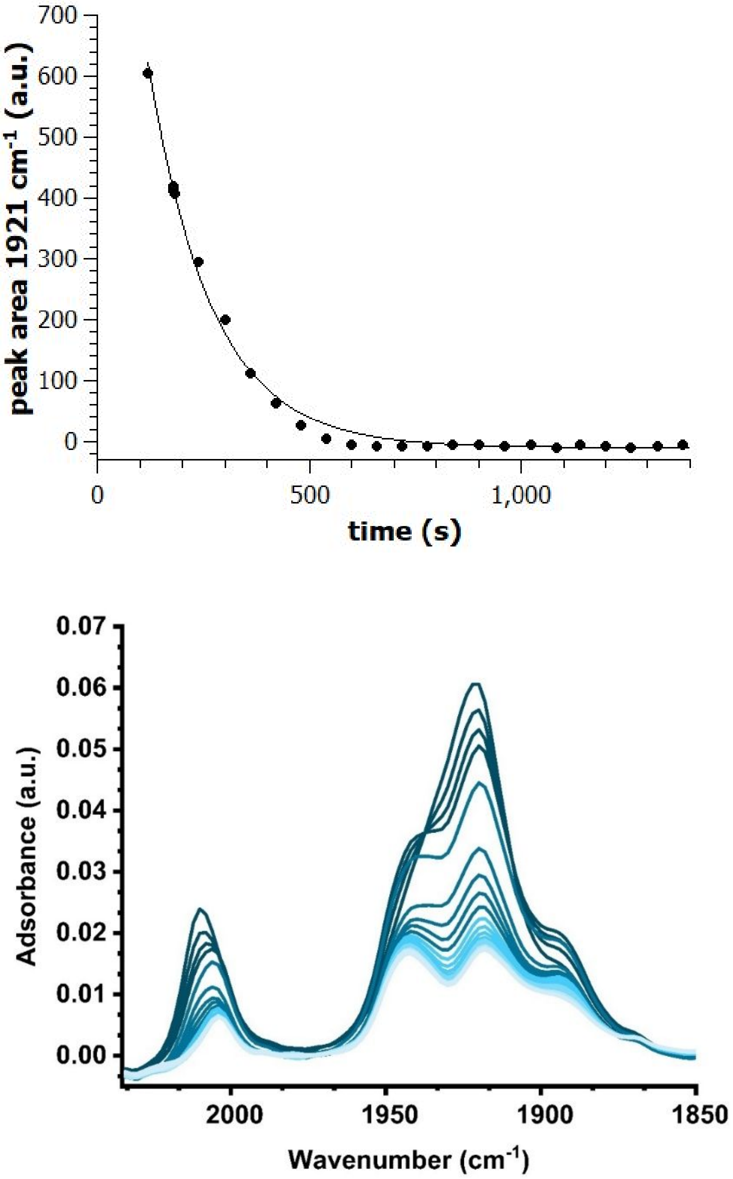

Figure 32. Absorption profiles of run 3 $CpCr(CO)_3H$ vs time (top) with selected IR spectra from 1850 cm^{-1} to 2040 cm^{-1} (bottom) during PICET to **V_2O_5-wires**.

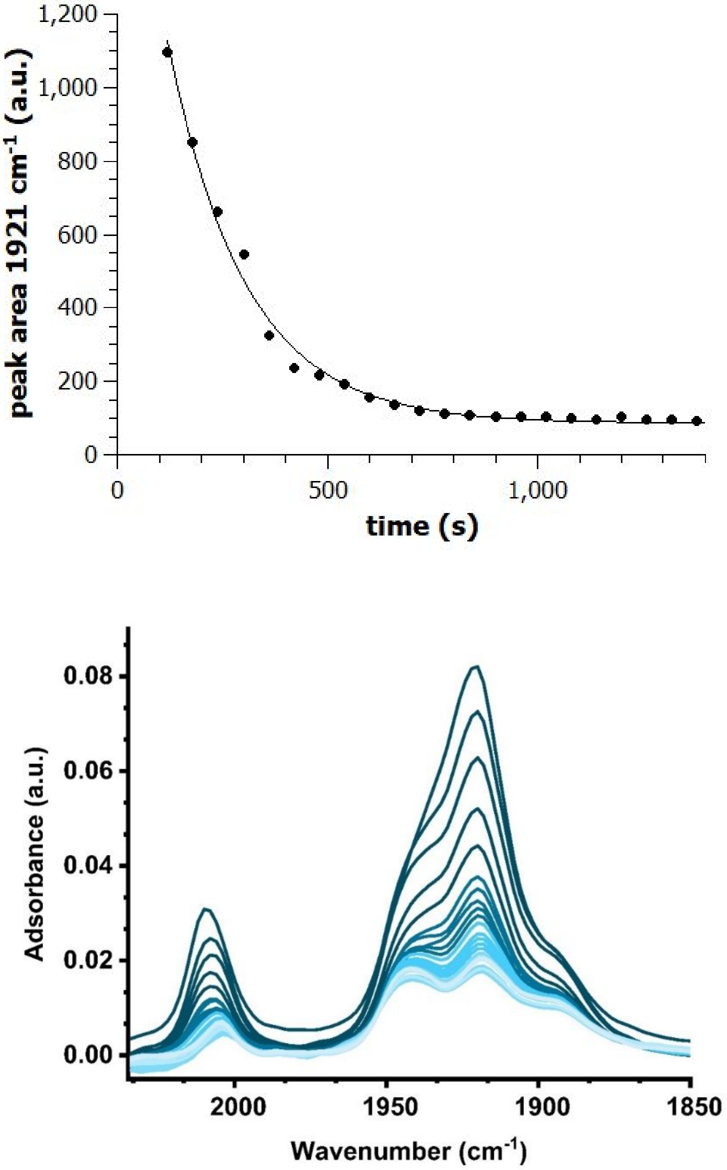

Figure S33. Absorption profiles of run 4 $CpCr(CO)_3H$ vs time (top) with selected IR spectra from 1850 cm^{-1} to 2040 cm^{-1} (bottom) during PICET to **V_2O_5-wires**.

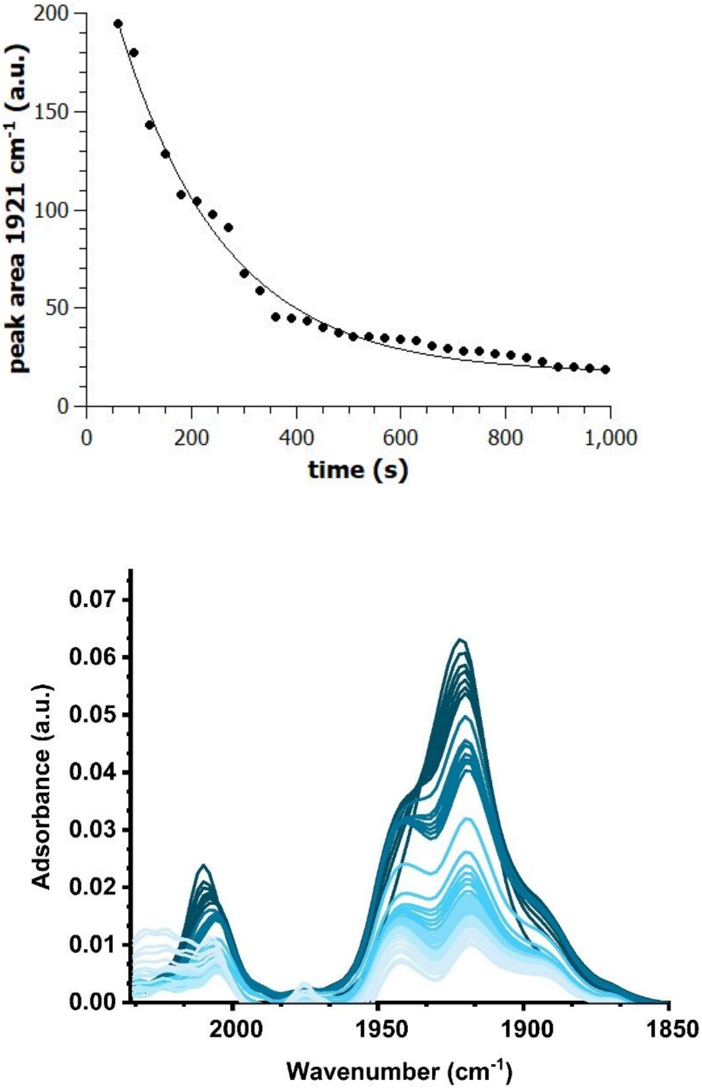

Figure S34. Absorption profiles of run 1 $CpCr(CO)_3H$ vs time (top) with selected IR spectra from 1850 cm^{-1} to 2040 cm^{-1} (bottom) during PICET to **V_2O_5-BM**.

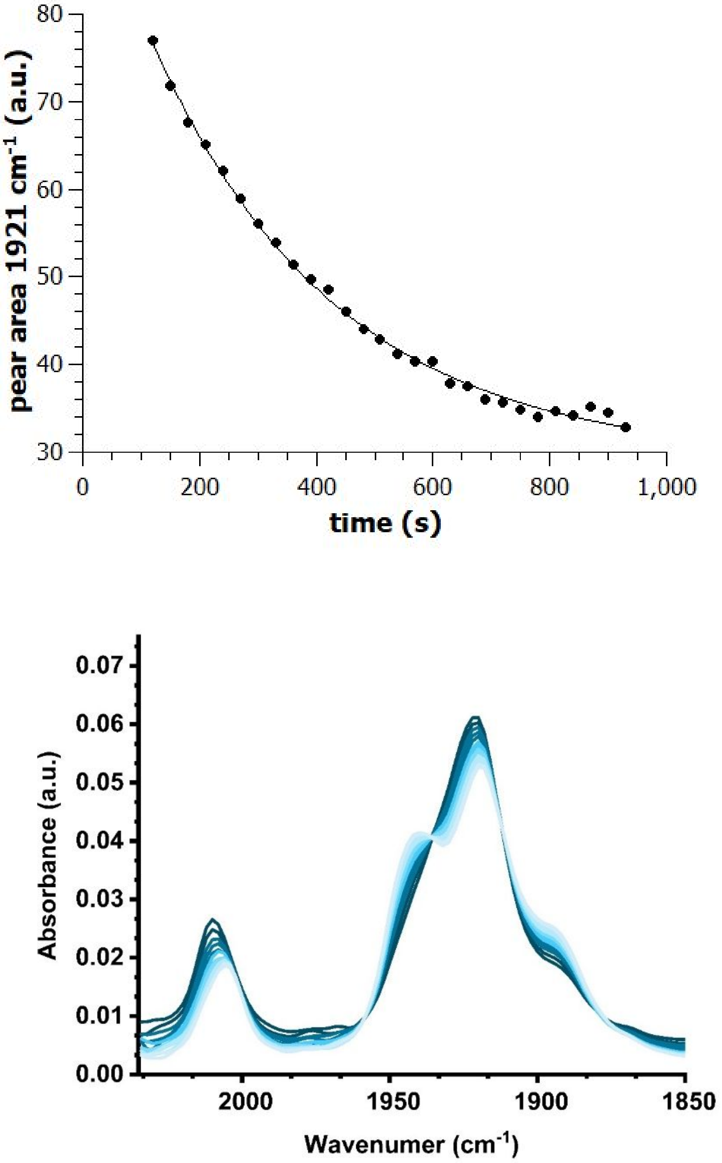

Figure S35. Absorption profiles of run 2 $CpCr(CO)_3H$ vs time (top) with selected IR spectra from 1850 cm^{-1} to 2040 cm^{-1} (bottom) during PICET to **V_2O_5-BM**.

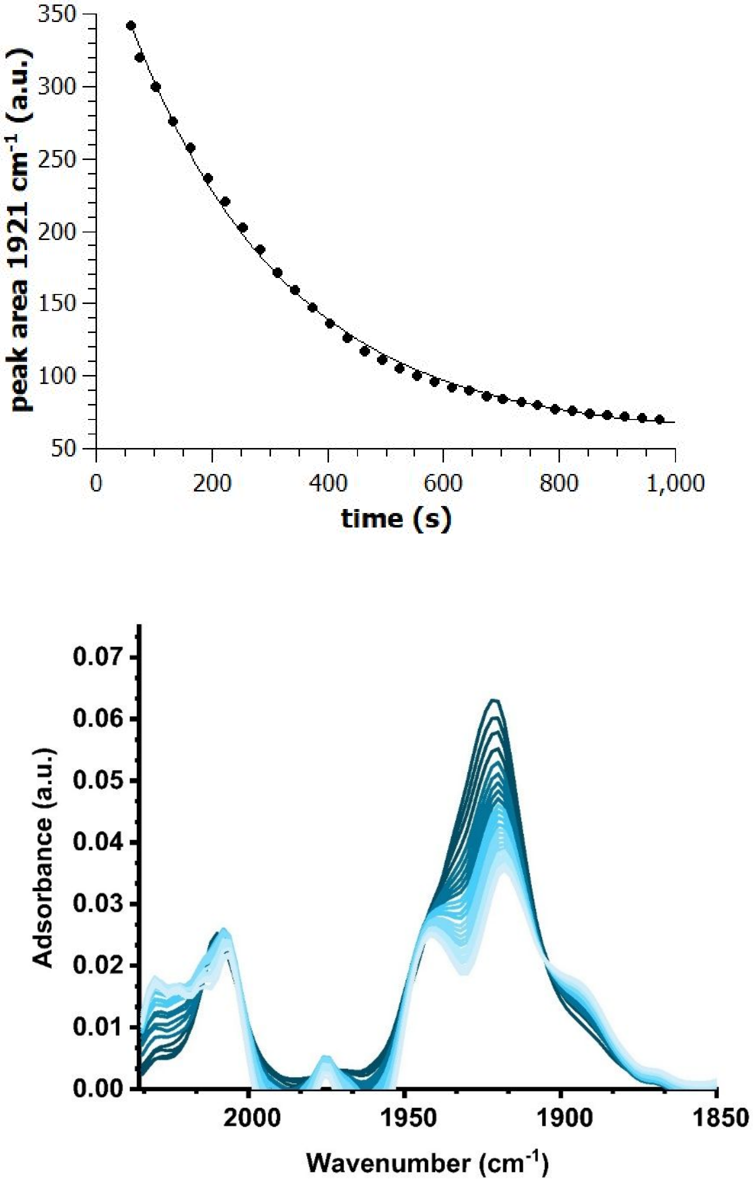

Figure S36. Absorption profiles of run 3 $CpCr(CO)_3H$ vs time (top) with selected IR spectra from 1850 cm^{-1} to 2040 cm^{-1} (bottom) during PICET to **V_2O_5-BM**.

Derivation of Rate Law for PICET from $CpCr(CO)_3H$ to V_2O_5

The rate of formation of reduced V_2O_5 most likely occurs via the following mechanism:

$$\text{Cr-H} + V_2O_5 \underset{k_{-1}}{\overset{k_1}{\rightleftharpoons}} \text{H*}/V_2O_5 + 1/_2\, Cr_2$$

$$\text{H*}/V_2O_5 \xrightarrow{k_2} V_2O_{5-2x}(OH)_{2x}$$

If we assume that the H* adsorbed on the surface of V_2O_5 is most likely in a steady state, we arrive at the following derivation:

$$\frac{dx}{dt} = \frac{1}{2} k_2 \theta_H \qquad (S1)$$

$$\theta_{H,ss} = \frac{k_1 [CrH] A_{BET}}{k_2 + k_{-1}\sqrt{[Cr_2]}\, A_{BET}} \qquad (S2)$$

$$iff\ k_2 \ll k_{-1}\sqrt{[Cr_2]}\, A_{BET} \quad then \quad \frac{dx}{dt} = \frac{k_1 k_2 [CrH]}{2\, k_{-1}\sqrt{[Cr_2]}} \qquad (S3)$$

One comes to a similar rate law by assuming that the reversible first step is in a rapidly maintained equilibrium compared to the transportation of the proton-electron pair into the bulk solid. This can be done by using the binding isotherm for PICET to solid surfaces developed by us in our previous work.[1] These rate laws agree with the fact that at low to medium conversions the reaction is 1st order in Cr-H and also that the kinetics are independent of the surface area of the material, provided that the transfer of H atoms from the V_2O_5 surface back to Cr is faster than their transfer into the bulk solid.

Estimation of the H atom loading dependence of the BDFE(V-OH) of V_2O_5 as measured by Dickens and coworkers[2]

In the work from Chippindale and Dickens, they measured the heats of reaction for the following reaction calorimetrically as a function of x_{max}.

$$x\,H_2 + V_2O_5 \xrightarrow[\text{3M KOH, r.t.}]{\text{Pt (cat)}} V_2O_{5-2x}(OH)_{2x}$$

They estimated the free energy of reaction by accounting for the entropy of gaseous H_2 that was lost upon reaction with the solid, but ignored the relatively small entropy changes between the solids. They do observe that this free energy of hydrogenation of V_2O_5 goes down as expected for higher and higher loadings of H. We have taken their data and reworked it in order to estimate the O-H BDFE as a function of hydrogen loading. We did this for each given x_{max} by subtracting the free energy obtained from the previous x_{max} and dividing that by the difference in x_{max} between the two samples. From this we subtracted 0.5*BDFE(H-H) in order to change from an H_2 reference to an H▪ reference system. An example is shown below.

$$0.23\,H_2 + V_2O_5 \xrightarrow[\text{3M KOH, r.t.}]{\text{Pt (cat)}} V_2O_{4.54}(OH)_{0.46} \qquad \Delta G_{rxn} = -7.1(5)\text{ kcal mol}^{-1}$$

$$-\quad 0.11\,H_2 + V_2O_5 \xrightarrow[\text{3M KOH, r.t.}]{\text{Pt (cat)}} V_2O_{4.78}(OH)_{0.22} \qquad \Delta G_{rxn} = +3.6(5)\text{ kcal mol}^{-1}$$

$$1/0.24\left(0.12\,H_2 + V_2O_{4.78}(OH)_{0.22} \xrightarrow[\text{3M KOH, r.t.}]{\text{Pt (cat)}} V_2O_{4.54}(OH)_{0.46} \qquad \Delta G_{rxn} = -3.6(5)\text{ kcal mol}^{-1}\right)$$

$$+\quad H^\bullet \longrightarrow 1/2\,H_2 \qquad \Delta G_{rxn} = -52\text{ kcal mol}^{-1}$$

$$H + 4.17\,V_2O_{4.78}(OH)_{0.22} \xrightarrow[\text{3M KOH, r.t.}]{\text{Pt (cat)}} 4.17\,V_2O_{4.54}(OH)_{0.46} \qquad \Delta G_{rxn} = -67(2)\text{ kcal mol}^{-1}$$

This value is the average BDFE(OH) for hydrogen atoms between these two x_{max} values. Therefore, it is more accurate to say that this is the BDFE(OH) for the average H▪ of this region and not of either of the edges of the region (for the example above, the free energy of the hydrogenation of V_2O_5 between x_{max} = 0.11 and 0.23 can be estimated as -67 kcal mol^{-1}. This value most closely represents the BDFE(OH) of neither 0.11 nor 0.23 itself but is rather an estimate of the BDFE(OH) at x_{max} = 0.17). The values thus derived are shown in table S5 below.

Table S5. Estimation of loading dependent BDFE(OH) of V_2O_5 from Dickens *et al.*.

x(average)	x_{max}	BDFE(V-OH)
0.055	0.11	68(3)
0.17	0.23	67(2)
0.475	0.72	67(1)
0.825	0.93	64(2)
1.16	1.40	59(2)
1.59	1.77	57(4)
1.82	1.88	56(4)

Catalytic Oxidation of Methanol to Formaldehyde

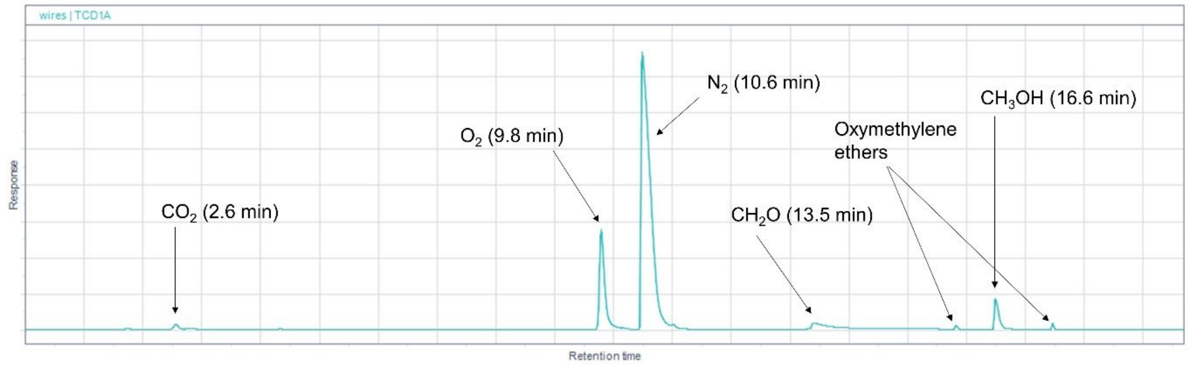

Figure S37. Example GC-TCD trace from the oxidation of methanol to formaldehyde at 250 °C and 20 mL min^{-1} flow rate using **V_2O_5-wires** as a catalyst showing the quantifiable products and reactants versus N_2 internal standard.

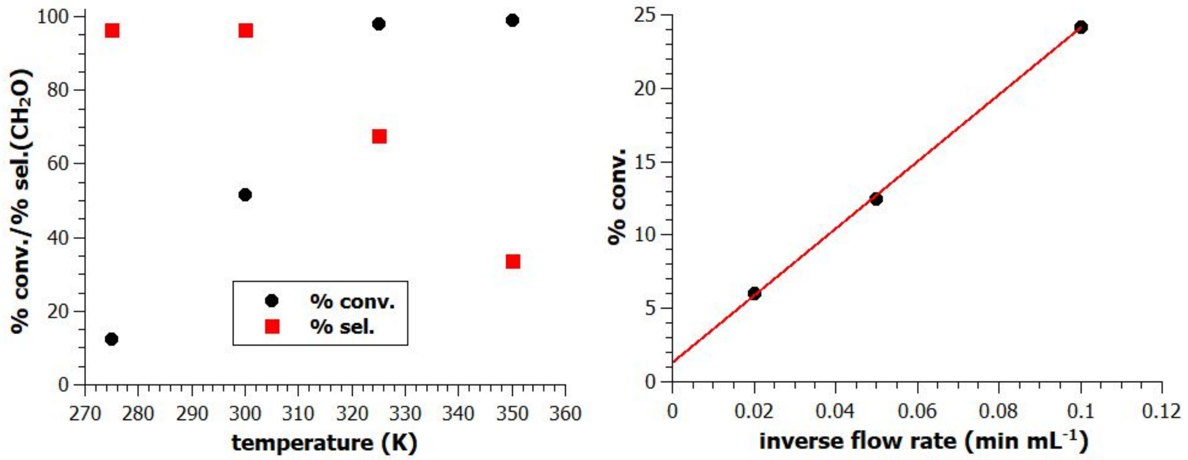

Figure S38. Relationship between both temperature (left) and inverse flow rate (right) and conversion/selectivity for **V_2O_5-comm** during the oxidation of methanol to formaldehyde demonstrating the stability of the catalyst over the course of the experiment.

References

[1] O. Bunjaku, J. S. Florenski, J. Wischnat, E. Klemm, O. V. Safonova, J. Van Slageren, D. P. Estes, *Inorg. Chem.* **2024**, *63*, 7512.

[2] A. M. Chippindale, P. G. Dickens, *Solid State Ionics* **1987**, *23*, 183-188.
